# Bioactive Diarylpentanoids: Insights into the Biological Effects beyond Antitumor Activity and Structure–Activity Relationships

**DOI:** 10.3390/molecules27196340

**Published:** 2022-09-26

**Authors:** Joana Moreira, Lucilia Saraiva, Madalena M. Pinto, Honorina Cidade

**Affiliations:** 1 Laboratory of Organic and Pharmaceutical Chemistry, Department of Chemical Sciences, Faculty of Pharmacy, University of Porto, Rua de Jorge Viterbo Ferreira 228, 4050-313 Porto, Portugal; 2 Interdisciplinary Centre of Marine and Environmental Research (CIIMAR), University of Porto, Edifício do Terminal de Cruzeiros do Porto de Leixões, Avenida General Norton de Matos, S/N, 4450-208 Matosinhos, Portugal; 3 LAQV/REQUIMTE, Laboratory of Microbiology, Department of Biological Sciences, Faculty of Pharmacy, University of Porto, Rua Jorge Viterbo Ferreira, 228, 4050-313 Porto, Portugal

**Keywords:** diarylpentanoids, C5-curcuminoids, biological activities, biochemical targets, structure–activity relationship

## Abstract

Diarylpentanoids, a class of natural products and their synthetic analogs which are structurally related to chalcones, have gained increasing attention due to their wide array of biological activities, including antitumor, anti-infective, antioxidant, anti-inflammatory, antidiabetic, anti-hyperuricemic, and neuroprotective properties. Previously, we reviewed diarylpentanoids with promising antitumor activity. However, in view of the wide range of biological activities described for this class of compounds, the purpose of this review is to provide a more detailed overview of the synthetic bioactive diarylpentanoids that have been described over the last two decades, beyond simply their antitumor effects. A total of 745 compounds were found, highlighting the main synthetic methodologies used in their synthesis as well as the structure–activity relationship studies and structural features for all activities reported. Collectively, this review highlights the diarylpentanoid scaffold as a promising starting point for the development of new therapeutic agents.

## 1. Introduction

**Figure 1:**
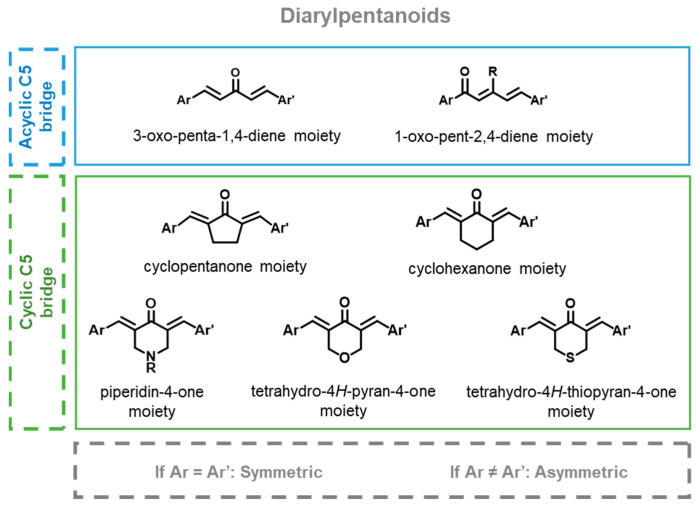
General structure of diarylpentanoids.

Diarylpentanoids comprise a class of natural products and their synthetic analogs possessing two aromatic rings, which may or may not have the same substitution pattern, which are linked by a five-carbon bridge. Most of these compounds possess an acyclic or cyclic C5 bridge with a dienone moiety linking the two aromatic rings, with the presence of symmetric or asymmetric linkers also being reported, as highlighted in Figure 1. These compounds show a wide array of biological activities, including antioxidant, anti-inflammatory, antitumor, and antibacterial properties among others. 

**Figure 2:**
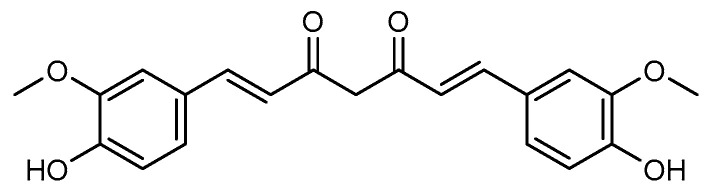
Curcumin (**1**).

The presence of an enone moiety between the two aromatic rings in the majority of these compounds makes them structurally related to chalcones, a well-known class of natural products with a wide range of biological activities [1,2,3,4]. Diarylpentanoids are also known as C5-curcuminoids since these are structurally related to curcumin (1*E*,6*E* -17-bis(4-hydroxy-3-methoxyphenyl)hepta-16-diene-35-dione (**1**: Figure 2), a C7-curcuminoid found in the traditional Indian herb *Curcuma Longa* [5]. This natural product (**1**) has shown diverse biological activities, such as antioxidant, antidiabetic, antimicrobial, antimalarial, antiviral, thrombosuppressive, antitumor, cardio- and neuro-protective properties, as well as chemopreventive activity [6,7,8,9,10] associated with different targets, which supports the idea that this compound influences a great variety of biochemical and molecular cascades [11]. However, curcumin (**1**: Figure 2) displays some limitations, such as chemical instability, poor aqueous solubility, low bioavailability, and fast metabolism under physiological conditions. These limitations have led to the development of several studies into synthetic curcumin analogs. Among these studies, the synthesis of novel curcumin analogs by molecular modifications, namely of the diketo group, was previously described [11]. The 1,3-dicarbonyl moiety is affected by in vitro and in vivo degradation through pathways including oxidation and hydrolysis. Suppression of the delocalization of electrons on the structure has been shown to improve curcuminoid stability and subsequent bioavailability [12]. On the other hand, the double bonds present on the linker bridge, although recognized as important for activity, are considered as part of the linker between the two essential structural elements and have not been widely modified [13]. In this scope, diarylpentanoids arose as an alternative.

Following our interest in this class of compounds [14,15,16,17], we previously reviewed diarylpentanoids with promising antitumor activity [18]. Nevertheless, considering the wide range of biological activities described for this class of compounds, and considering the inexistence of exhaustive review articles focusing on other biological activities of diarylpentanoids, this study is intended to review these diverse biological effects beyond the reported antitumor activity. Moreover, some insights about the synthetic methods used to obtain these compounds, as well as the mechanisms involved in these activities and the putative structural features relevant for the respective biological activities, are provided. Data concerning synthesis and structure–activity relationship studies (SAR) of diarylpentanoids, organized by biological activity and by chronological order of the year in which they were first reported, are presented in the next sections and in Table S1 (Supplementary Materials).

## 2. Synthesis of the Diarylpentanoids

**Scheme 1:**
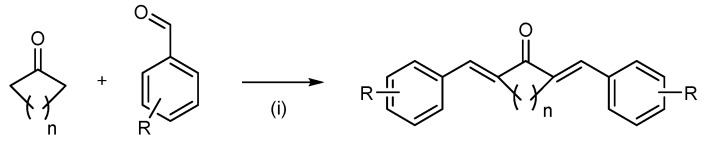
General synthesis of symmetric diarylpentanoids. The ketone could be acetone (*n* = 0), cyclopentanone (*n* = 2), or cyclohexanone (*n* = 3). Reaction conditions (i): NaOH or HCl.

The most employed method for the synthesis of diarylpentanoids is Claisen–Schmidt condensation between a suitable ketone and a substituted benzaldehyde in the presence of a polar solvent while using a strong base or acid (Scheme 1). For example, Du et al. (2006) synthesized symmetric diarylpentanoids by coupling the substituted aromatic aldehyde with ketone under acidic conditions and used a ratio of ketone:aldehyde of 1:2 at 25–30 °C [19]. Lee et al. (2009) synthesized a series of symmetric diarylpentanoids in the presence of NaOH in ethanol [20].

**Scheme 2:**
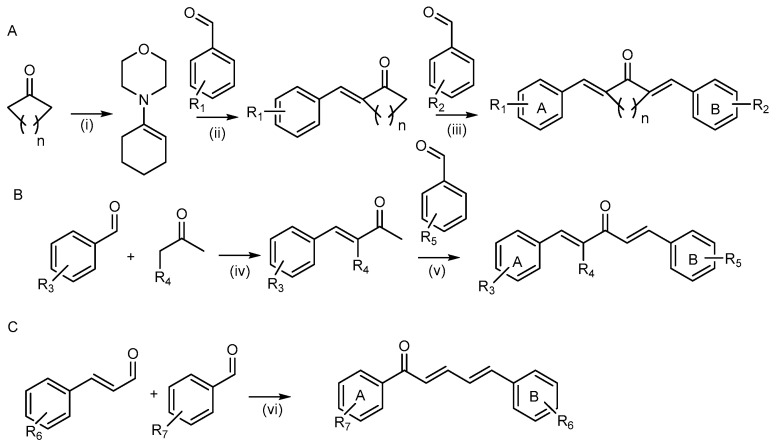
General synthesis of asymmetric diarylpentanoids. (**A**) The ketone could be acetone (*n* = 0), cyclopentanone (*n* = 2), or cyclohexanone (*n* = 3). Reaction conditions: (i) morpholine; (ii) HCl; (iii) NaOH or HCl. (**B**) Reaction conditions: (iv) HCl gas, DMC, rt; (v) NaOH in EtOH, rt. (**C**) Reaction conditions: (vi) NaOH, ethanol, rt (overnight).

However, to obtain asymmetric compounds with different aryl rings, a monoaryl intermediate should be firstly prepared by Stork reaction and subsequently condensed with the appropriately substituted aldehyde to give the target compounds (Scheme 2A). When asymmetry is present in the C5 bridge, different reaction conditions are used depending on the C5 bridge. In the case of diarylpentanoids with an α-substituted enone moiety, the intermediates are first prepared by condensation using HCl as a catalyst, and the intermediate is then treated with substituted benzaldehyde in the presence of bases (Scheme 2B) [21]. Faudzi et al. (2015) performed the synthesis of diarylpentanoids, with the 1-oxo-pent-2,4-diene moiety as an acyclic C5 bridge, through the reaction of an appropriately substituted cinnamaldehyde and a ketone under alkaline/ethanol conditions (Scheme 2C) [22].

## 3. Biological Activity of Diarylpentanoids

### 3.1. Anti-Infective Activity

Infectious diseases caused by pathogens, such as bacteria, fungi, and viruses, are major challenges for humankind as multidrug resistance to antibiotics, antivirals, and antiparasitic agents is one of the major causes of the alarming level of infectious diseases worldwide [23]. The discovery of new drugs with potent anti-infective activity, particularly against the resistant strains, is therefore highly desirable. Diarylpentanoids have been reported as a promising class of compounds with anti-infective activity, namely antibacterial and antiparasitic activity. 

#### 3.1.1. Antibacterial Activity

**Figure 3:**
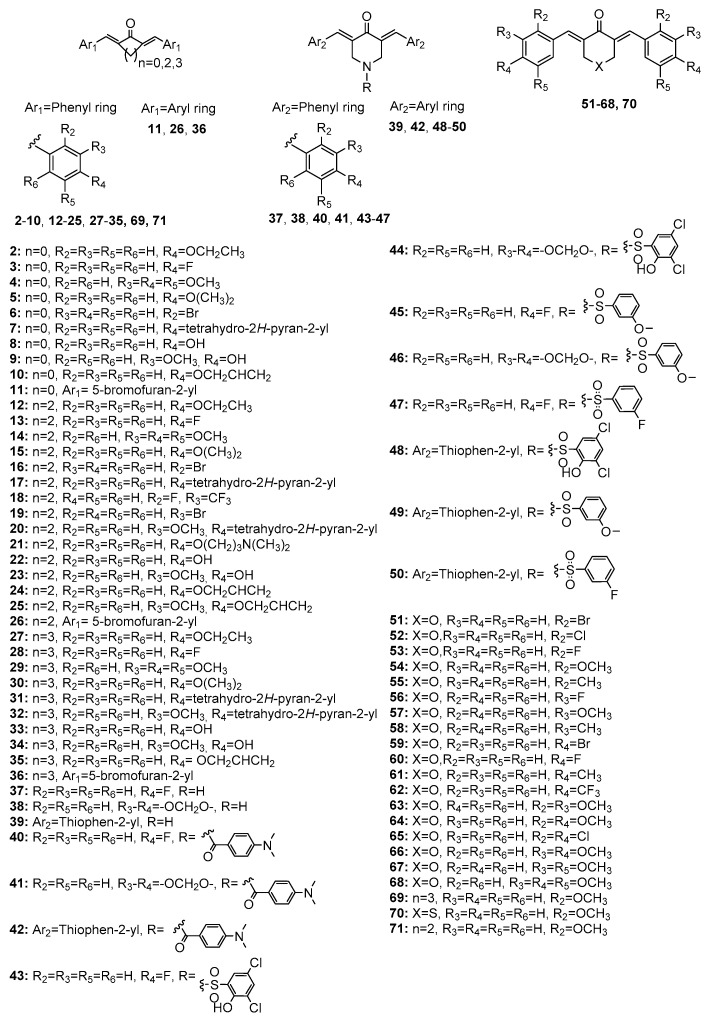
Diarylpentanoids exhibiting antibacterial activity.

**Figure 4:**
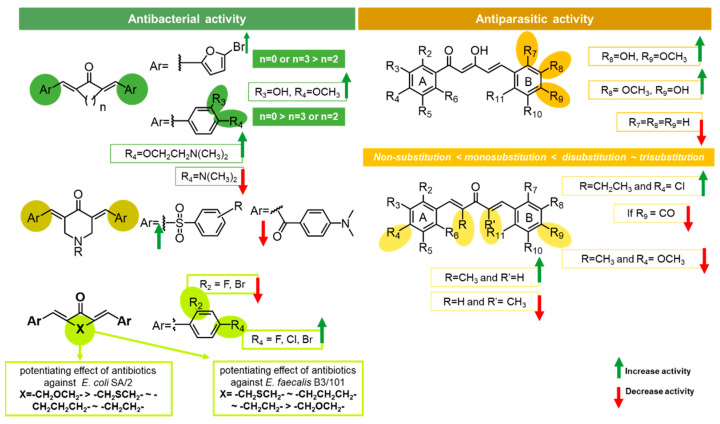
Putative SAR for the anti-infectious activity of diarylpentanoids.

In 2008, Liang et al. synthesized three series of symmetric diarylpentanoids, including compounds with an acyclic C5 bridge (**2**–**11**: Figure 3) and with a C5 bridge embodied in a cyclic moiety, namely cyclopentanone (**12**–**26**: Figure 3 A origem da referência não foi encontrada.) and cyclohexanone (**27**–**36**: Figure 3). Their in vitro antibacterial activity against seven Gram-positive and Gram-negative bacteria were tested by determining the zone of inhibition. Compounds **8**, **9**, **11**, **21**, and **36** (inhibition zone (IZ) = 9–20 mm) showed remarkable in vitro antibacterial activity. It was observed that some of the derivatives displayed significant activity when compared to curcumin (**1**), and most of them exhibited activity against ampicillin-resistant *E. cloacae*. These results allow for some structure–activity relationship (SAR) considerations, as highlighted in Figure 4. It was shown that the presence of heterocyclic aryl rings (**11**, **26**, and **36**) was associated with an enhancement of activity, with diarylpentanoids with a C5 acyclic bridge (**11**) and with a cyclohexanone moiety (**36**) being more potent than those with a cyclopentanone moiety (**26**) (Figure 4). The comparison of biological data obtained for diarylpentanoids with phenyl rings reinforces that the C5 bridge can have some influence on antibacterial activity (Figure 4). Additionally, a long-chain amino substituent in compound 21 may play an important role in bioactivity (Figure 4) [24]. 

Some symmetric diarylidene piperidone derivatives (**37**–**50**: Figure 3) were synthesized by Selvekumar et al. (2010) and screened for their antibacterial activity using the agar diffusion method. Most of the synthesized compounds showed moderate activity against *S. typhi* and *V. cholera* and were poorly active against the other bacteria. In particular, all derivatives of 2-hydroxy-3,5-dichloro phenylsulfonamides (**43**, **44**, and **48**) showed promising activity against *S. typhi* and *V. cholera* (IZ = 10–20 mm) (Figure 4). On the other hand, the carboxamide derivatives **40**–**42** were not active compared to sulfonamide derivatives (**43**–**50**) (Figure 4) [25]. 

In 2022, our group reported the synthesis of a library of symmetric diarylpentanoids (**51**–**71**: Figure 3) and investigated their potential as antimicrobial agents and as reversers of antimicrobial resistance [17]. The compounds were investigated for their antimicrobial activity in susceptible and resistant bacterial and fungal strains. Although none of the tested compounds showed activity against the used bacterial and fungal strains (MIC > 100 µM or MIC > 64 µg/mL for bacteria and MIC > 128 µg/mL for fungi), most of the compounds displayed promising results in combination with antibacterial drugs, with diarylpentanoid **63** being the most promising. Considering the results obtained, some fundamental SAR guidelines were established (Figure 4). For *E. coli* SA/2, diarylpentanoids with a single *para*-substituent with an electron-withdrawing effect in the B phenyl group (**59**, **60**, and **62**) were more potent than the *ortho*-substituted analogs (**51**–**53**). For compounds with di- or trisubstituted phenyl rings, substitution at the *ortho*- or *para*-position appeared to be crucial for activity (**63**–**66** and **68**) since, when none of these positions was substituted, non-active compounds were obtained (**67**). Comparing the effects of diarylpentanoids with the same aryl rings and different C5 bridges, it seems that the presence of a linker with a tetrahydro-4*H*-pyran-4-one moiety (**52**–**54**, **58**–**66**, and **68**) was much more favorable for a positive effect in terms of cefotaxime activity than cyclohexanone (**69**), tetrahydro-4*H*-thiopyran-4-one (**70**), and cyclopentanone (**71**) moieties. Curiously, for *E. faecalis*, the presence of a linker with a cyclohexanone (**69**), tetrahydro-4*H*-thiopyran-4-one (**70**), or cyclopentanone (**71**) moiety was more favorable than a linker with a tetrahydro-4*H*-pyran-4-one (**52**–**54**, **59**, **60**, **62**, **64**–**66**, and **68**) moiety [17]. 

The compounds that displayed promising results were also evaluated for their activity in efflux pump-related virulence mechanisms, such as biofilm formation and quorum-sensing. The set of compounds **59**, **63**, **66**, and **69** (Figure 3) was considered to be active toward the inhibition of efflux pumps, mainly in *Staphylococcus aureus* 272123. Compound **63** inhibited biofilm formation by *S. aureus* 272123 and by Sphingomonas paucimobilis Ezf 10–17 and Chromobacterium violaceum CV026, while compound **69** only inhibited biofilm formation by *S. aureus* 272123 [17]. 

#### 3.1.2. Antiparasitic Activity

**Figure 5:**
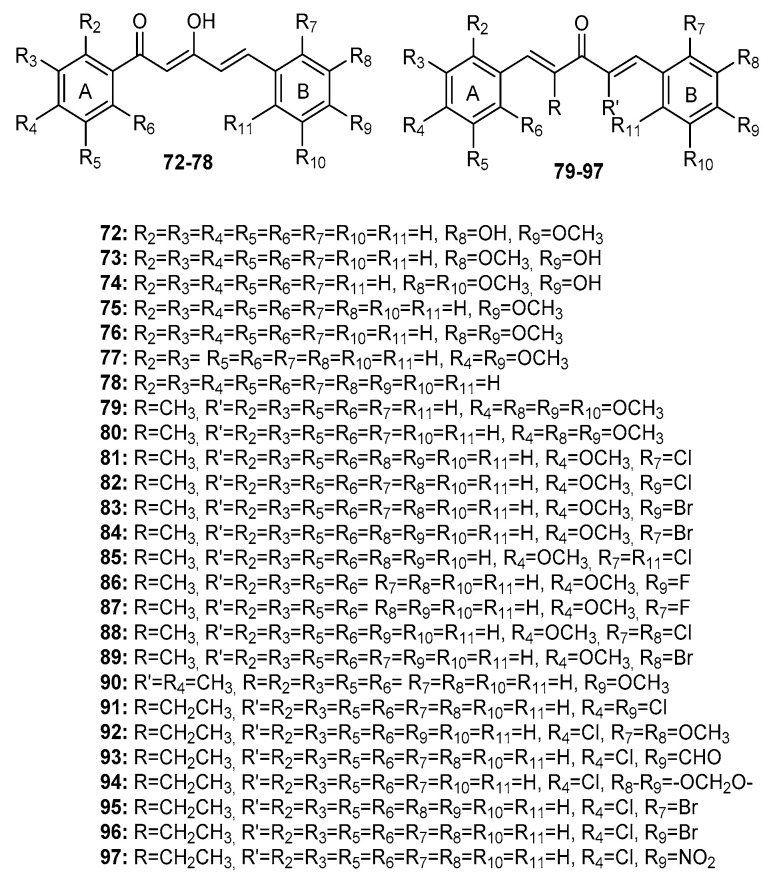
Diarylpentanoids with promising antiparasitic activity.

Leishmaniasis is a group of prevalent diseases caused by protozoan parasites of the genus Leishmania. Alves et al. (2003) reported the in vitro antileishmanial activity of a new series of asymmetric diarylpentanoids (**72**–**78**: Figure 5) against *L. amazonensis*, *L. braziliensis*, and *L. chagasi* promastigotes. It was observed that diarylpentanoids were more effective against *L. braziliensis* promastigotes (IC_50_ values between 23 and 78 µM) than against *L. amazonensis* and *L. chagasi* (IC_50_ values ranging from 3 to 373 µM and 6 to 310 µM, respectively). Interestingly, the most active diarylpentanoid against *L. braziliensis* contained *p*-hydroxyl and *m*-dimethoxy substituents in one of the aromatic rings (B ring) (**74**). On the other hand, against *L. amazonensis* and *L. chagasi* promastigotes, the most effective derivatives have *m*-hydroxyl and *p*-methoxy substituents (**72**) in the B ring or *p*-hydroxyl and *m*-methoxy groups (**73**) in the B ring [26] (Figure 4). Generally, when comparing the overall results, we can infer that the presence of unsubstituted (**78**) or monosubstituted (**75**) B ring is associated with a decrease in antileishmanial activity. 

The synthesis and evaluation of the antiproliferative activity of asymmetric diarylpentanoids (**79**–**97**: Figure 5) for promastigotes of *L. amazonensis* and epimastigotes and trypomastigotes of *T. cruzi* has been reported [21]. It was inferred that these curcuminoids have potent antiproliferative activity against studied parasites, with compounds containing an ethyl substituent at the α-position to the carbonyl group and a *p*-chloro substituent in the A ring (**70**–**76**: IC_50_ = 0.5–74.2 µM) being more potent than those with a methyl group at the α-position to the carbonyl group and a *p*-methoxy substituent in the A ring (**79**–**90**: IC_50_ = 3.7–>100 µM) (Figure 4). Considering the results reported for the most potent derivatives (**91**–**97**), different substitutions in the B ring were accepted, except for the presence of a *p*-carbonyl group, which was associated with a decrease in activity (Figure 4). For derivatives **79**–**90**, it was observed that di- or tri-methoxy substitution on the aromatic B ring was more favorable for activity than *ortho-* or *para*-halogenated substitution on the same ring (Figure 4). Interestingly, the derivative with a *p*-methyl group in the A ring and a methyl group at the α-position to the carbonyl group (**90**) presented a reduction in activity when compared to the other tested compounds (Figure 4). The binding interactions of potent compounds were predicted through molecular docking computational studies, and strong interactions with the enzyme trypanothione reductase (pdb code 1BZL) were found [21]. 

### 3.2. Antioxidant Activity

Free radicals and other reactive oxygen species, such as superoxide radical anions, hydroxyl radicals, and hydrogen peroxide, are constantly generated through many biological processes. When the generation of these species are not balanced with natural antioxidants they can lead to oxidative alterations at the cellular and molecular levels, which are associated with various pathologies, such as cancer, cardiovascular diseases, atherosclerosis, hypertension, ischemia/reperfusion injury, diabetes mellitus, neurodegenerative diseases, rheumatoid arthritis, and aging [27,28,29,30]. Thus, the removal of free radicals from biological systems is very important for the sustainability of cellular machinery. A series of diarylpentanoids were prepared, and antioxidant activity was evaluated and compared to curcumin (**1**) by the scavenging of the diphenylpicrylhydrazyl (DPPH) free radical assay and other assays.

**Figure 6:**
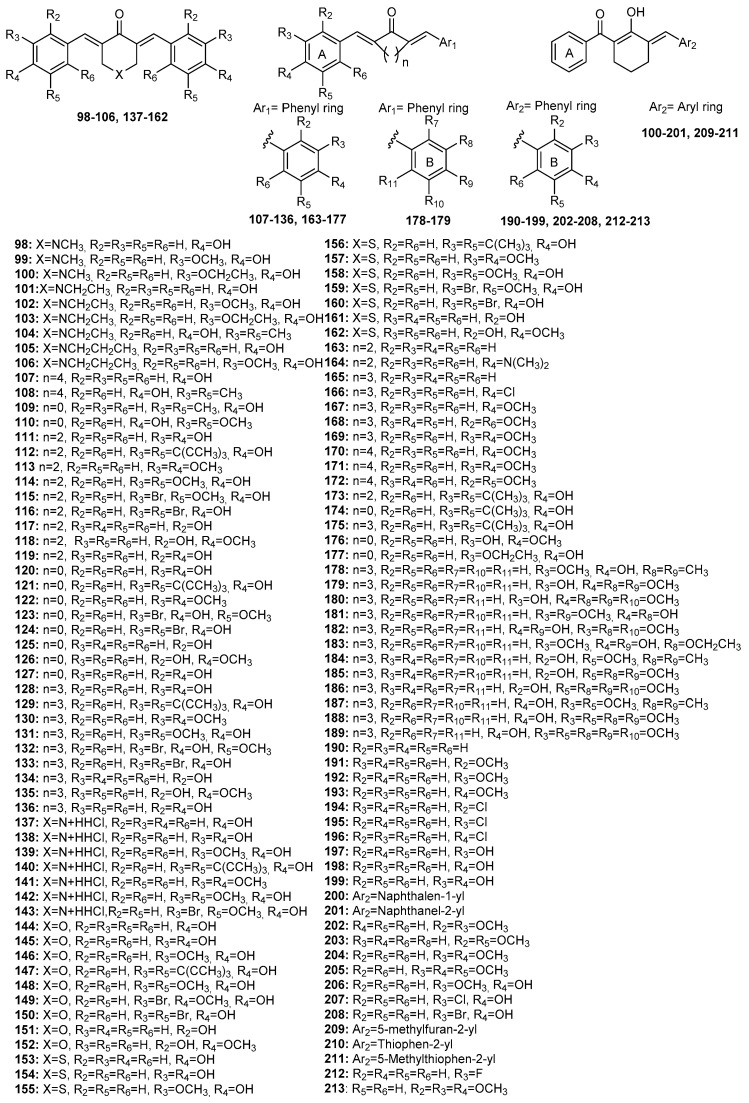
Diarylpentanoids as promising antioxidant agents.

**Figure 7:**
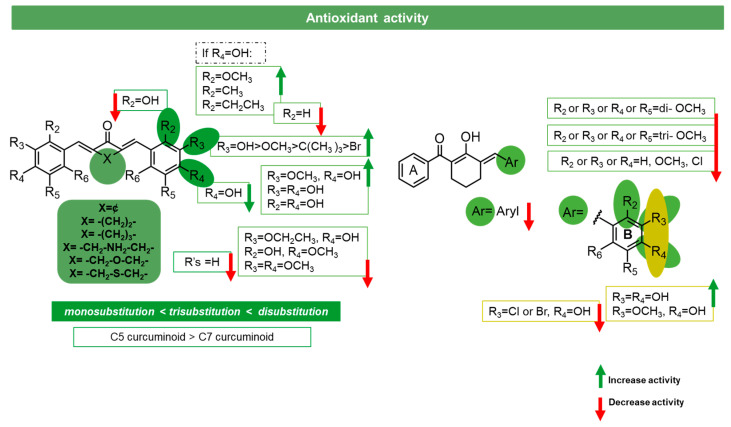
Putative SAR for the antioxidant activity of diarylpentanoids.

Youssef et al. (2004) reported a new series of diarylpentanoids (**8**: Figure 3; **98**–**110**: Figure 6) for their in vitro DPPH scavenger activity. The free radical scavenging activity was also measured by chemiluminescence using polymorphonuclear leukocytes (PMNs). All compounds showed high or moderate free radical scavenger activity compared to curcumin (**1**), with compound **103** showing the highest percentage of inhibition. In addition, the results demonstrated that the presence of electron-donating substituents on the *meta-* and *para*-position of aromatic rings (**99**, **100**, **102**–**104**, **106**: K = 7.30–16.32 min^−1^ × 10^−2^) is associated with enhanced antioxidant activity when comparing to compounds with only p-hydroxy substituents (**98**: K = 2.15 min^−1^ × 10^−2^; **101**: K = 3.06 min^−1^ × 10^−2^; **105**: K = 14.80 min^−1^ × 10^−2^). Additionally, the activity is potentiated by increasing the lipophilicity of the piperidone ring through substitution of methyl (**98**) with either ethyl (**101**) or propyl moieties (**105**) (Figure 7). Comparing the effects of diarylpentanoids with the same aryl rings and different C5 bridges, it seems that the presence of a piperidone (**98**–**106**) or cycloheptanone (**107** and **108**) moiety is much more favorable than the presence of a 3-oxo-penta-1,4-diene moiety (**8**, **109**, and **110**) (Figure 7) [31]. 

A series of diarylpentanoids were synthesized by Du et al. by coupling aromatic aldehyde with cyclopentanone (**8**, **22**, **23**, and **34**: Figure 3; **111**–**119**: Figure 6), 3-oxo-penta-1,4-diene (**109**, **110**, and **120**–**127**: Figure 6), cyclohexanone (**33**: Figure 3; **128**–**136**: Figure 6), tetrahydrothiopyran-4-one (**137**–**143**: Figure 6), tetrahydropyran-4-one (**66**, **135**–**152**: Figure 6), or tetrahydrothiopyran-4-one (**153**–**162**: Figure 6). Compounds with catechol groups (**102**, **120**, **128**, **138**, **145**, and **154**) exhibited the best DPPH scavenging effect (IC_50_ = 5.79–29.26 µM) amongst the compounds investigated (Figure 7). The activities of these compounds decreased when R2, R3, or R4 were replaced by bromine, methoxy, *t*-butyl, or hydrogen, with diarylpentanoids with 2-phenol groups being much weaker than diarylpentanoids with 4-phenol groups (Figure 7) [32].

In another study, three cyclopentanone derivatives (**22**: Figure 3; **163** and **164**: Figure 6) were synthesized and assessed for antioxidant activity using a DPPH free radical scavenging method. Symmetric compounds with *p*-hydroxyl (**22**) or *p*-dimethylamine (**164**) groups in aromatic rings showed good antioxidant activity with IC_50_ values of 49.1 µg/mL and 64.6 µg/mL, respectively, which is comparable to the control (ascorbic acid: IC_50_ = 51.5 µg/mL) and much better than the unsubstituted diarylpentanoid (**163**: IC_50_ > 200 µg/mL) [33]. 

A series of diarylpentanoids containing a cyclohexanone (**33** and **34**: Figure 3; **165**–**169:** A origem da referência não foi encontrada.) or cycloheptanone (**170**–**172**: Figure 6) moiety were prepared by Bayomi et al. (2013) and tested for their antioxidant activity using a 2,2’-azinobis(3-ethylbenzothiazoline-6-sulfonicacid) radical cation (ABTS+) scavenging assay. All compounds exhibited weak inhibition (0–9.49%), except compound **34** (74.22%). This compound bearing *p*-hydroxyl and *m*-methoxy groups at the aromatic rings was the best antioxidant among the compounds investigated (Figure 7) and presented similar results to curcumin (**1**) and ascorbic acid [34]. 

Chen et al. (2014) designed and synthesized 27 diarylpentanoids with different central moieties, including cyclopentanone (**22**, **23**, **33**, and **34**: Figure 3; **111**, **113**–**115**, and **173**: A origem da referência não foi encontrada: Figure 6), 3-oxo-penta-1,4-diene (**8** and **9**: Figure 3; **110**, **120**, **122**, **123**, and **174**: Figure 6), cyclohexanone (**33** and **34**: Figure 3; **128**, **130**–**132**, and **175**: Figure 6), and piperidone (**137**–**140**, **133**, and **143**: Figure 6) moieties, and evaluated their DPPH scavenging effect. It was observed that compounds **111**, **120**, **128**, and **138** were clearly the most active (IC_50_ < 3 µM) comparing to the positive control’s vitamin C and curcumin (**1**). All results indicated that, when the *para*-position has a hydroxyl group, DPPH scavenging activity depends significantly on the introduction of electron-donating groups (hydroxyl and methoxy) in the *meta*-position (Figure 7). It is obvious that the replacement of hydroxyl with methoxy in the *para*-position (**113**, **122** and **130**) significantly decreases activity (IC_50_ > 800 µM) (Figure 7). Moreover, the results showed that the *meta*-substitution of the aromatic rings by hydroxyl groups is more favorable for activity than substitution by methoxy groups. However, the presence of methoxy groups is associated with a higher effect than the presence of *t*-butyl groups, which are also more favorable for activity than bromine (Figure 7). Interestingly, when comparing compounds with different central moieties but the same substitution pattern regarding aromatic rings, no significant differences are observed in scavenging activity [35]. 

In 2015, Li et al. prepared five symmetric (**4** and **9**: Figure 3; **122**, **176**, and **177**: A origem da referência não foi encontrada.) and twelve asymmetric (**178**–**189**: Figure 6) acyclic diarylpentanoids. All the compounds were tested for their ability to scavenge free radicals using four assays (DPPH, ABTS, ROO• radical scavenging (TRAP), and O^2-•^ radical scavenging (NET) assay). According to the data, all compounds showed stronger antioxidant activities than vitamin C (IC_50_ = 45.41–167.31 µM), except for symmetric diarylpentanoids **34** and **122** (IC_50_ > 4819 µM and IC_50_ > 4819 µM, respectively) (Figure 7). Compounds **9** and **188** showed the most significant antioxidant activity (IC_50_ = 9.24–72.62 µM and IC_50_ = 13.51–80.93 µM, respectively), even compared to curcumin (**1**: IC_50_ = 13.53–89.82 µM). Interestingly, compound **9**, which has the same substituent pattern at the aromatic rings as curcumin (**1**) but which has a different linker, exhibited stronger activity than curcumin (**1**) (Figure 7) [36].

In the same year, newly synthesized 2-benzoyl-6-benzylidene cyclohexanone analogs (**190**–**213**: Figure 6) were investigated for their antioxidant activity in both DPPH radical scavenging and ferric reducing antioxidant power (FRAP) assays [37]. The results revealed that diarylpentanoid **199** with a catechol group displayed significant activity in both DPPH (IC_50_ = 11.5 µM) and FRAP (0.756 mmol AAE/L) assays, with the DPPH scavenging effect being double that of curcumin (**1**: IC_50_ = 23.6 µM), while it was equipotent to butylated hydroxytoluene in the FRAP scavenging assay (0.803 mmol AAE/L). Compound **206** showed promising results but was worse than curcumin (**1**) [37]. From these data, we can infer some SAR considerations as follows. Compounds with a single *ortho-*, *meta-*, or *para*-substituent have similar potency independent of the nature of the substituent, namely chloro, methoxy, or hydroxyl groups (Figure 7). For compounds with di-substituted phenyl rings, the *meta*- and *para*-positions appear to be the most effective. Moreover, the presence of catechol or *m*-methoxy and *p*-hydroxyl groups were important for potency. Nevertheless, the presence of a dimethoxy group in the *meta*- and *para*-positions leads to loss of activity (Figure 7). 

### 3.3. Anti-Inflammatory Activity

The persistence of inflammation in the body leads to numerous inflammatory diseases, including pancreatitis, arthritis, inflammatory bowel disease, colitis, gastritis, allergies, autoimmune diseases, cardiovascular problems, atherosclerosis diabetes, arthritis, neurodegenerative diseases, and cancer [38]. The use of anti-inflammatory drugs is associated with several side effects, including gastrointestinal and renal toxicity. Therefore, the development of more efficient and less toxic drugs is progressing worldwide. Anti-inflammatory effects through the diverse mechanisms of diarylpentanoids are well documented. Among these mechanisms, the regulation of short-lived bioactive free radical nitric oxide (NO), as well as pro-inflammatory and inflammatory cytokines and transcription factors, and regulation of enzymatic activity such as that related to cyclooxygenase (COX) and lipoxygenase (LOX) has been highlighted.

#### 3.3.1. Effect of Diarylpentanoids on NO Production 

NO, a potent pro-inflammatory mediator, is synthesized by the catalytic reaction of nitric oxide synthase (iNOS) in response of inflammatory stimuli [39]. Abnormal expression of iNOS and excessive generation of NO is expressed in response to various stimuli (lipopolysaccharide (LPS), interferon-gamma (IFN-γ), or tumor necrosis factor-alpha (TNF-α)) [40], which enhances the production of diverse inflammatory mediators and leads to alterations in physiological and pathophysiological mechanisms, resulting in inflammatory disorders [41,42,43,44,45]. 

**Figure 8:**
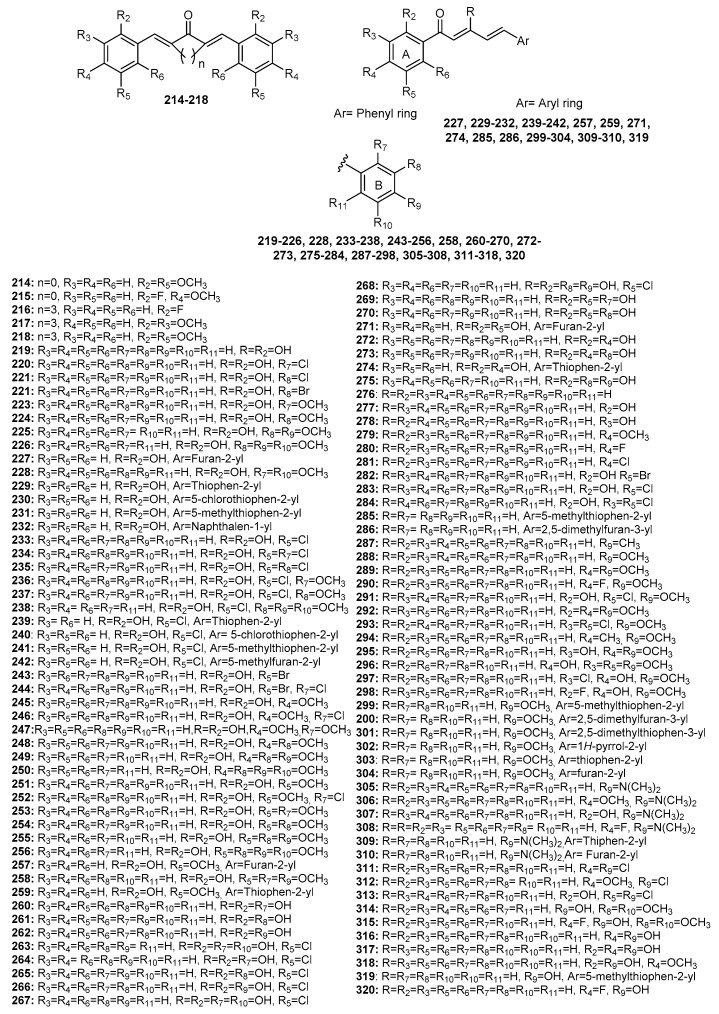
Synthetic diarylpentanoids with promising inhibition of NO production.

**Figure 9:**
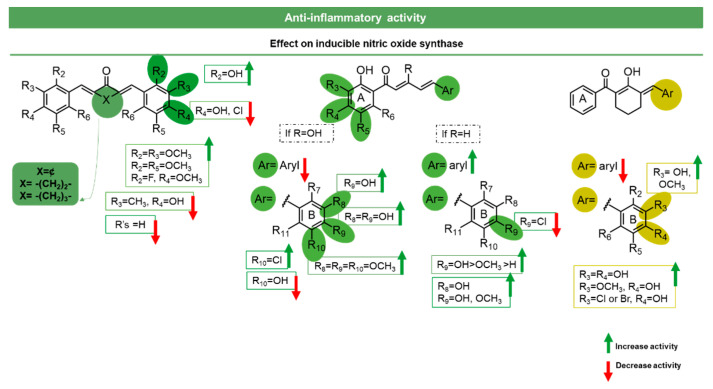
Putative SAR for the effects of diarylpentanoids with promising inhibition of NO production.

In a study conducted by Lee et al. (2009), a new series of diarylpentanoids with an acyclic C5 bridge (**125**, **214**, and **215**: Figure 8) and a C5 bridge embodied in a cyclohexanone moiety (**28**: Figure 3; **217**–**209**: Figure 8) were synthesized and evaluated for their effect on the NO production assay [20]. Amongst the tested compounds, diarylpentanoids **125**, **214**, and **218** showed a potent NO inhibitory effect in RAW macrophages 264.7 (IC_50_ value of 10.24 ± 1.05 µM, 13.64 ± 0.41 µM, and 13.66 ± 0.61 µM, respectively) as compared to positive control Nω- nitro-L-arginine methyl ester hydrochloride (L-NAME), IC_50_ = 27.13 ± 5.58 µM) and curcumin (**1**: IC_50_ = 20.38 ± 0.28 µM). The presence of 2,5-dimethoxylated phenyl groups (compounds **214** and **218**) and 2-hydroxylated phenyl groups (compound **125**) were thought to contribute toward their potent anti-inflammatory action (Figure 9). Interestingly, compounds **125** and **214** showed NO inhibitory effect in a dose-dependent manner, and compound **198** demonstrated potent inhibitory action toward NO production, without causing any cytotoxicity effect, at all concentrations tested [20].

In addition to antioxidant activity, compound **22** (Figure 3), as well as compounds **163** and **164** (Figure 6), was also tested for its anti-inflammatory activity using the murine monocytic macrophage cell line RAW 264.7. Compound **163**, with no additional substituent on both aromatic rings, showed weak activity (32.62% inhibition of NO production), while compounds **22** and **164** showed moderate activity (57.77% and 58.62% inhibition of NO production, respectively) compared to the positive control (L-NAME, 82.84% inhibition of NO production) [33].

In 2014, Leong et al. synthesized a series of 97 asymmetric diarylpentanoids (**219**–**275**: Figure 8). The IFN-γ/LPS-stimulated macrophages cells were used to assess anti-inflammatory activity through the NO suppression assay. Among all the series, 12 compounds (**226**, **238**, **240**, **244**, **255**, **256**, **262**–**264**, **266**, **268**, and **275**: IC_50_ = 13.6–19.8 µM) exhibited greater or similar NO inhibitory activity when compared to curcumin (**1**: IC_50_ = 14.7 ± 0.2 µM), with compounds **268** and **275** being the most potent with IC_50_ values of 4.9 ± 0.3 µM and 9.6 ± 0.5 µM, respectively. An SAR study revealed findings in accordance with a previous study, where the presence of a hydroxyl group in both aromatic rings was critical for bioactivity (Figure 9). Moreover, the presence of a catechol moiety on the B ring appeared to be an important contributing factor since it resulted in a two- to four-fold improvement in bioactivity (Figure 9). Further structure–activity comparisons showed that the presence of an electron-withdrawing group on the A ring was important for enhancing the NO inhibitory activity. In contrast, replacing the B phenyl ring with a heterocyclic aromatic ring was found to be undesirable (Figure 9) [46]. 

In continuation with previous work, Leong et al. [37] investigated 2-benzoyl-6-benzylidene cyclohexanone analogs (**190**–**213**: Figure 6) for their in vitro NO inhibitory effects in an LPS/IFN-induced RAW 264.7 macrophages model. The results revealed that compounds **192**, **197**, **206**, and **207** moderately suppress NO production (IC_50_ = 22.7–35.3 µM), and compounds **199** and **208** exhibited the highest activity with an IC_50_ value of 4.2 ± 0.2 and 15.2 ± 0.8 µM, respectively. Based on these results, it appears that the presence of hydroxyl and methoxy groups in the B ring was imperative for the inhibition of NO production, with the *meta*-hydroxylated (**197**) and *meta*-methoxylated (**192**) positions being preferable over *para*-hydroxylated (**198**) and *para*-methoxylated (**191** and **193**) positions (Figure 9) [37].

Another study in 2015 reported a newly synthesized series of diarylpentanoids (**276**–**320**: Figure 8) with anti-inflammatory activity by measuring their NO inhibition activity in IFN-γ/LPS-activated RAW 264.7 cells [22]. Among the tested compounds, **295**, **316**, **319**, and **320** showed similar or greater activity (IC_50_ = 10.2–16.4 µM) than curcumin (**1**: IC_50_ = 14.7 ± 0.2 µM), with the most significant inhibition of NO production being observed for compound **319** (IC_50_ = 10.24 ± 0.62 µM), a 5-methylthiophenyl-bearing analog. Additionally, compounds **278**, **296**–**298**, **304**, **317**, and **318** significantly inhibited NO production, with IC_50_ values ranging between 20.0–30.0 µM. Generally, it was observed that the chemical nature of both rings (A and B) plays a significant role in enhancing anti-inflammatory activity in the diarylpentadien-1-one compounds (Figure 9). Apparently, the presence of a *para*-hydroxyl group on the B ring is the feature that contributes to NO inhibitory activity. In turn, the presence of *meta*- or *para*-hydroxyl groups on the A ring is the feature that contributes to NO inhibitory activity, whereas electron-withdrawing groups disfavor the activity (Figure 9). On the other hand, the replacement of the phenyl A ring with a heterocyclic ring, such as thiophenyl or furanyl, resulted in mild inhibition of NO production (Figure 9). The biological implication of structural features along with 2D and 3D quantitative structure–activity relationship (QSAR) analyses were performed. It was concluded that a hydroxyl group on the *para*-position of the B ring and an α,β-unsaturated ketone moiety on the linker were important features in the enhanced inhibition of NO production. Additionally, ADMET analysis was performed using Discovery Studio 3.1, and results indicated that compounds presented adequate drug-like properties [22]. 

#### 3.3.2. Modulation of Pro-Inflammatory Cytokines and Transcription Factors by Diarylpentanoids

Pro-inflammatory cytokines, such as TNF-α, and interleukins, particularly IL-6, are critically involved in inflammation and related disorders, including ulcerative colitis, diabetes, multiple sclerosis, atherosclerosis, and septic shock. The pro-inflammatory effects of TNF are primarily due to its ability to activate the transcription factors (nuclear factor kappa-light-chain-enhancer of activated B cells (NF-κB) and activator protein 1 (AP-1)) responsible for regulating the expression of pro-inflammatory genes involved in controlling cellular proliferation/growth, inflammatory responses, and cell adhesion. Therefore, anti-inflammatory agents that inhibit the overexpression of pro-inflammatory cytokines are of great interest for the clinical treatment of many inflammatory diseases.

**Figure 10:**
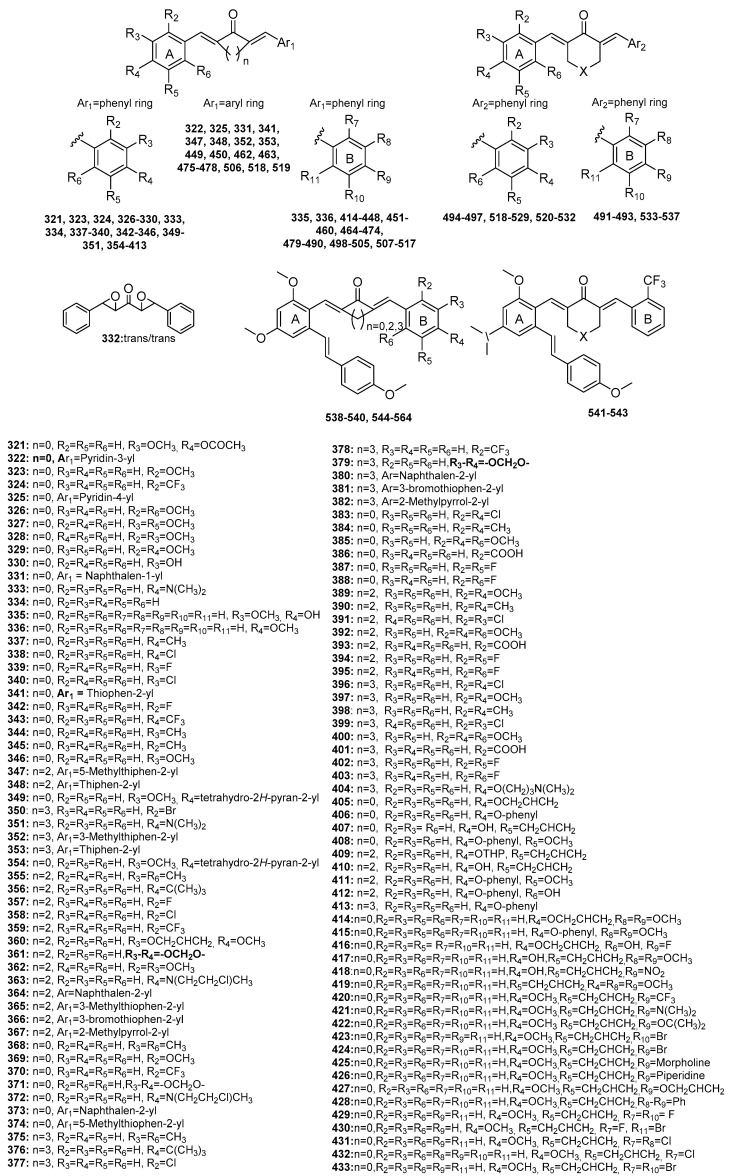

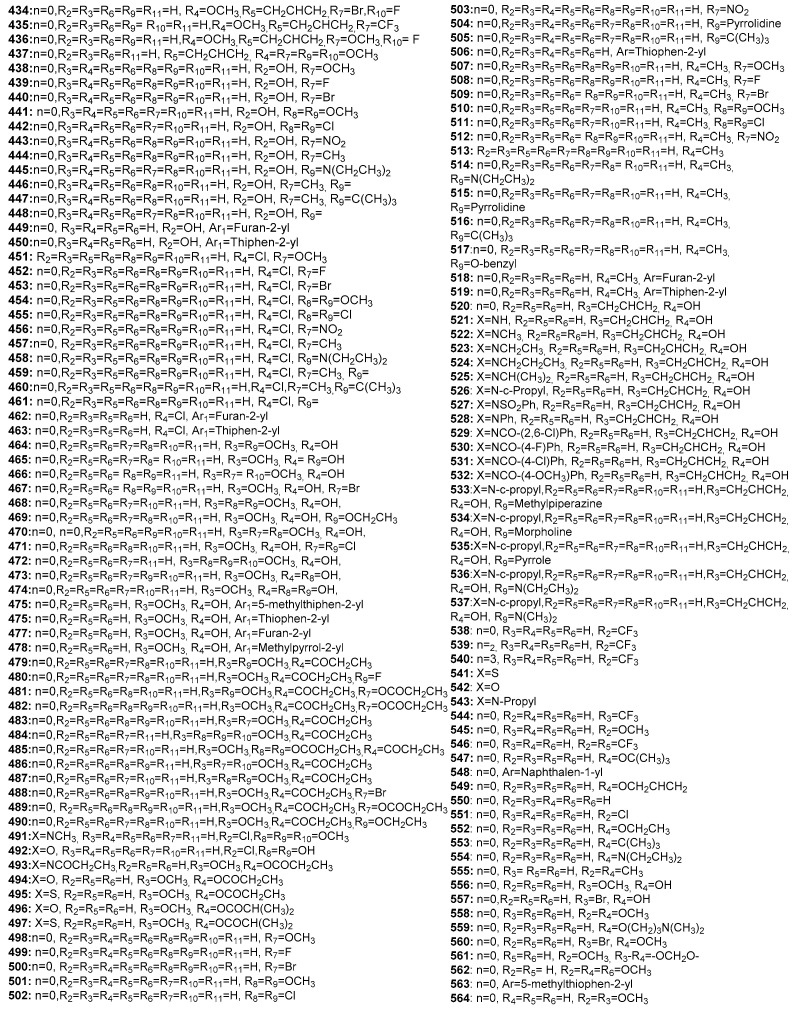
Diarylpentanoids with a promising inhibitory effect on pro-inflammatory cytokines.

In 2006, Weber et al. synthesized and evaluated the anti-inflammatory activity of a series of diarylpentanoids (**3**, **8**, **9**, and **34**: Figure 3; **120**, **122**, **125**, and **165**: Figure 6; **214**, Figure 8; and **321**–**346**: Figure 10) against the TPA-induced activation of AP-1 using the panomics AP-1 Reporter 293 stable cell line. Analogs **34**, **122**, **125**, **215**, and **321**–**333** inhibited the activation of AP-1 and showed activities comparable to (**122** and **329**–**333**) or better (**34**, **125**, **214**, and **321**–**328**: IC_50_ = 1.4–11.4 µM) than curcumin (**1**: IC_50_ value of IC_50_ = 12.8 ± 0.5 µM). In contrast, analogs **3**, **8**, **9**, **120**, **165**, and **334**–**346** were enhancers of the TPA-induced activation of AP-1 [47]. 

**Figure 11:**
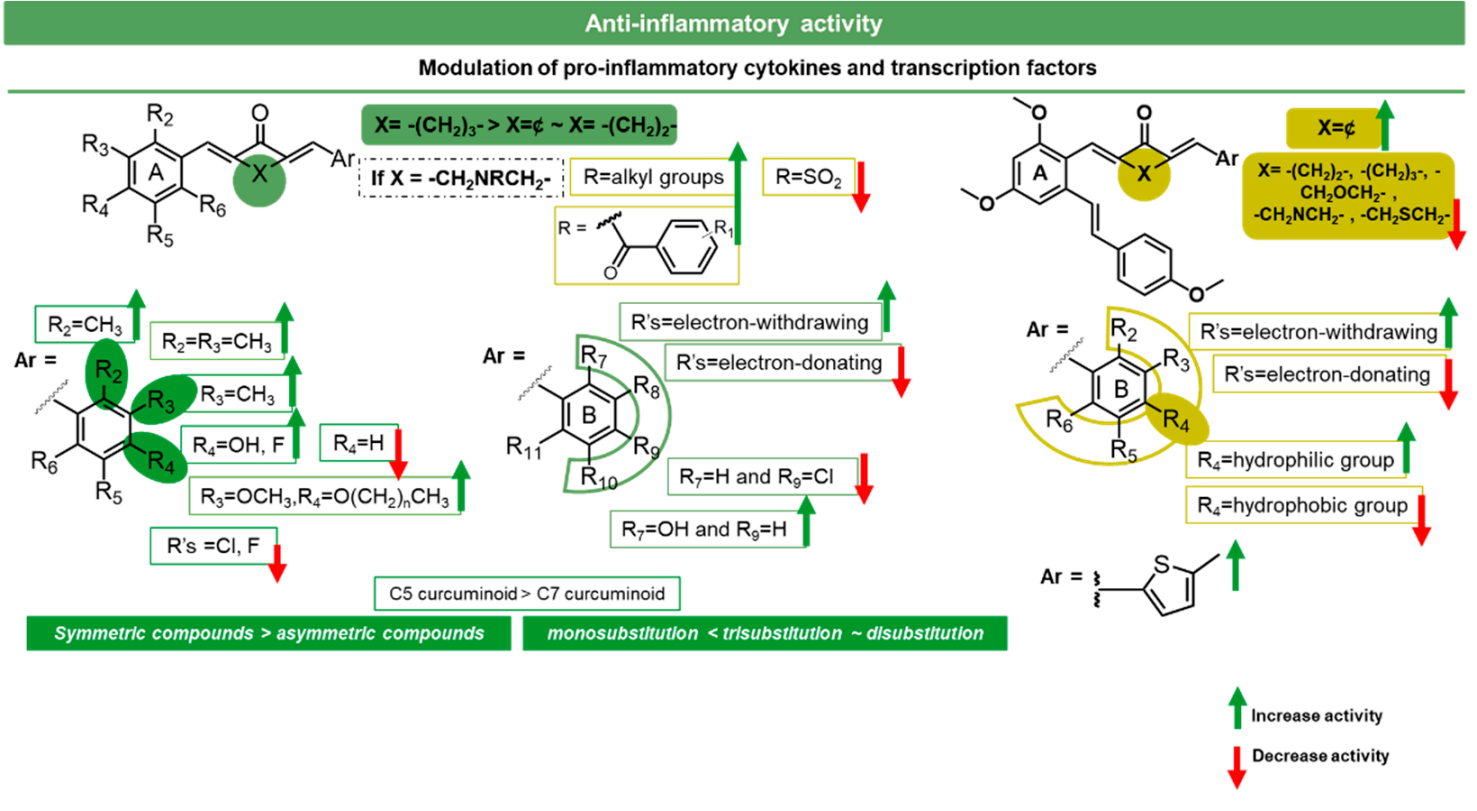
Putative SAR for the effects of diarylpentanoids on the modulation of pro-inflammatory cytokines and transcription factors.

In the attempt to develop potent anti-inflammatory agents, three series of diarylpentanoids with a cyclopentanone (**12**–**14**, **17**, **20**–**23**, and **26**: Figure 3; and **164**, **347**, and **348**: Figure 10), 3-oxo-penta-1,4-diene (**2**, **3**, **6**–**11**: Figure 3; **340** and **349**: Figure 10), or cyclohexanone (**27**–**29**, **31**–**36**: Figure 3; and **350**–**353**: Figure 10) moiety were synthesized and tested for in vitro anti-inflammatory activity by using enzyme-linked immunosorbent assays (ELISA), which measured the ability to inhibit LPS-induced TNF-αand IL-6 expression in J774A.1 mice [48]. The results indicated that curcumin (**1**) and its analogs (10 µM) inhibited LPS (0.5 µg/ mL)-induced TNF-α and IL-6 expression. However, diarylpentanoids with a cyclohexanone moiety (**27**–**29**, **31**–**36**, and **350**–**353**: Figure 10) were more effective than those with an acyclic C5 bridge (**2**, **3**, **6**–**11**, **340**, and **349**: Figure 10) and those with a cyclopentanone moiety (**12**–**14**, **17**, **20**, **21**–**23**, **26**, **164**, **347**, and **348**). SAR studies revealed the importance of different substituents on the aromatic ring. Compound **21**, with dimethylamino propoxyl, has a more potent inhibitory effect on LPS-induced IL-6 expression than curcumin (**1**) but showed a similar inhibitory effect on LPS-induced TNF-α expression as curcumin (**1**). However, other compounds with similar substituents (**164** and **351**) showed less inhibitory effects than curcumin (**1**), indicating that the anti-inflammatory effects did not increase with the presence of amino groups by themselves. On the other hand, the length and flexibility of the substituent groups may be favorable to the inhibitory effect on LPS-induced TNF-α (**10** and **35**), and the presence of heterocyclic-substituted compounds (**36**, **347**, and **348**) may be accepted. Interestingly, compounds **9**, **23**, and **34**, which have the same substituents as curcumin (**1**) but have a C5 bridge, exhibited stronger activity, while compounds **8**, **22**, and **33**, with only a *para*-hydroxyl group in phenyl rings, had less or opposite activities, suggesting that the presence of a *meta*-methoxy group is critical to activity (Figure 11) [48].

Later, the same group obtained and tested diarylpentanoids **3**, **7**, **13**, **17**, **20**, **28**, **31**, and **32** (Figure 3), as well as **354** (Figure 10), for their anti-inflammatory activity against LPS-induced TNF-α and IL-6 secretions using J774.1 mice macrophages [49]. Compounds *meta*-methoxylated (**20**) and *para*-halogenated (**28**) in the phenyl rings exhibited the maximum anti-inflammatory activity at a concentration of 10 µM with 40% TNF-α inhibition and 50% IL-6 inhibition, respectively. The remaining compounds showed moderate activity at the same concentration. The results showed that the presence of *meta*-methoxy groups played an important role in bioactivity (**20**, **32**, and **354**) compared to unsubstituted compounds (**7**, **17**, and **31**), and compounds with a cyclohexanone moiety exhibited stronger inflammatory inhibition than compounds with a cyclopentanone moiety or an acyclic C5 bridge (Figure 11), which conforms with a previous study [48]. Single-crystal XRD was then performed, and it was concluded that the presence of a cyclohexanone moiety may play an important role in bioactivity [49].

In 2019, Liang et al. also evaluated diarylpentanoids **8**, **9**, **21**–**23**, **33**, **34**, and **71** (Figure 3); **216** and **217** (Figure 8); and **341**, **347**, **348**, **352**, **353**, and **355**–**382** (Figure 10) for their inhibitory activities against LPS-induced TNF-α and IL-6 release in macrophages. Most of the tested compounds proved to be more potent than curcumin (**1**) in inhibiting LPS-induced TNF-α or IL-6 expression, with compounds **9**, **21**, **23**, **33**, **34**, **217**, **325**, **358**, **365**, **368**–**372**, **374**, and **379** being the stronger inhibitors against both TNF-α and IL-6. Compound **368**, possessing *ortho*- and *meta*-dimethyl-substituted phenyl rings, showed the strongest inhibitory effect on LPS-induced TNF- α and IL-6 release (34.3% and 15.9%, respectively) (Figure 11). From the active analogs above, four compounds, **21**, **23**, **355**, and **374**, were selected for further evaluation of their inhibitory effects against LPS-induced TNF-α and IL-6 release. Compounds **21**, **23**, and **374** demonstrated comparable inhibitory effects to curcumin (**1**). The results of this study were in accordance with the data obtained previously by Liang et al. (2009) [49].

Synthesis and anti-inflammatory evaluation of a series of diarylpentanoids with different C5 bridges (acyclic bridge: **329**, **383**–**388**, Figure 10; cyclopentanone moiety: **389**–**395**, Figure 10; and cyclohexanone moiety: **396**–**404**, Figure 10) was reported by Zhao et al. (2010) [50]. Among these compounds, **329**, **384**, **385**, **388**, **390**, **400**, and **404** exhibited the highest inhibitory effect on LPS-induced TNF-α production, and compounds **329**, **384**, **385**, **389**, **388**, **393**, **400**, and **404** showed inhibitory effects on LPS-induced IL-6 production, with **388** and **404** being more potent than curcumin (**1**) in the production of both cytokines in a dose-dependent manner. From these results, some SAR considerations may be undertaken (Figure 11). Contradicting the study conducted by Liang et al. (2009), it was suggested that compounds with a C5 acyclic bridge were found to be more effective than compounds with cyclopentanone or cyclohexanone moieties. Additionally, the electron-donating ability of the *para*-substituent on the aromatic rings might be responsible for enhancing the anti-inflammatory abilities of diarylpentanoids **329**, **384**, **385**, **389**, **390**, **392**, **397**, **398**, and **400**, whereas compounds without substituents at the *para*-position (**386**, **387**, **391**, **393**, **394**, **399**, **401**, and **402**) and an electron-withdrawing moiety (**384**, **329**, **396**) may reduce bioactivity. Additionally, aminated analogs (**404**) may be considered as promising compounds for developing anti-inflammatory candidates (Figure 11) [50]. 

Another novel series of allylated or prenylated diarylpentanoids (**405**–**437**: Figure 10) were designed, synthesized, and investigated against TNF-α and IL-6 expression induced by LPS using mouse RAW 264.7 macrophages. The majority of the tested compounds inhibited the LPS-induced expression of TNF- α and IL-6, particularly IL-6 expression, at a dosage of 10 µM. Their inhibitory abilities were comparable to or more pronounced than curcumin (**1**) at the same concentration. It was seen that compounds **407**, **433**, **434**, and **437** exhibited stronger inhibition of both TNF-α and IL-6 than curcumin (**1**), with **433** being the most potent on LPS-induced TNF-α and IL-6 production with its inhibitory rates reaching 67.5% and 91.1%, respectively. Compound **433** also exhibited significant protection against LPS-induced death in septic mice. SAR studies suggested that the electronic properties of the substituents on the aromatic rings seem to have no influence on bioactivity, and the di- or tri-substituted compounds are much more active than mono-substituted compounds (Figure 11) [51].

Zhang et al. (2014) synthesized 26 asymmetric diarylpentanoids (**438**–**465**: Figure 10) and evaluated their effect on the inhibition of LPS-induced TNF-α and IL-6 release in a model of mouse RAW 264.7 macrophages. The reported data revealed that all tested compounds presented stronger anti-inflammatory activity than curcumin (**1**). However, compounds **438**, **440**, **441**, **447**, and **449** manifested highly significant and dose-dependent anti-inflammatory activity. Additionally, the inhibition of cytokine production caused by compounds **438** and **440** was associated with the inhibition of ERK phosphorylation and the NF-κB pathway. Furthermore, compound **440** exhibited significant protection against LPS-induced septic death in vivo. The overall results suggest that the existence of a methoxy group in the B ring, and the presence of heterocyclic rings (**449** and **450**), is associated with increased anti-inflammatory activity (Figure 11). However, the inhibitory effect on IL-6 production is enhanced when an electron-donating hydroxyl group is positioned in the A phenyl ring (**438**–**450**), while an electron-withdrawing chlorine in the A phenyl ring has little effect (**451**–**465**) (Figure 11) [52].

In 2014, Zhang et al. also reported asymmetric (**464**–**492**: Figure 10) and symmetric (**493**–**497**: Figure 10) diarylpentanoids as potent anti-inflammatory agents. Some of the synthetic analogs exhibited similar or more potent inhibitory ability than curcumin (**1**), with symmetric compounds **493**–**497** as well as an asymmetric compound **493** with a tetrahydro-4*H*-thiopyran-4-one moiety being the most effective as inhibitors of LPS-induced TNF-α and IL-6 secretion (with a range of 59.5%–83.4%) in mouse macrophages. A compound with a thiopyranone moiety (**497**) showed the most potent activity, with an inhibition of 98.7%. Mechanistically, compound **493** significantly inhibited the LPS-induced phosphorylation of ERK. Furthermore, diarylpentanoids **493** and **496** displayed a significant protective effect on LPS-induced septic death in mouse models, with 40% and 50% survival rates, respectively. The results showed that molecular symmetry and electronegativity might play a crucial role in the anti-inflammatory activity of these diarylpentanoids (Figure 11) [53]. 

A series of symmetric 1,5-diphenyl-3-oxo-penta-1,4-dienes (**498**–**519**: Figure 10) were synthesized and evaluated for their anti-inflammatory activity using in vitro assays and in vivo models [54]. Most of the tested compounds significantly reduced the production of TNF-α and IL-6 in LPS-stimulated macrophages when compared to curcumin (**1**). Compounds **503**, **506**, **512**, and **519** exhibited a high degree of inhibitory effects on TNF-α expression, and compounds **501**, **503**, **507**, **508**, **510**, **517**, and **519** showed remarkable downregulation of IL-6 expression stimulated by LPS, with **503**, having an *ortho*-nitro-substituted phenyl ring, possessing the most potent inhibition of LPS-induced IL-6 and TNF-α production (an inhibition rate of 54.53% and 91.20%, respectively). The mechanism of action for compound **503** might be associated with inhibition against LPS-induced NF-κB and ERK pathway activation. Further, treatment with **503** effectively prolonged survival in a mouse model of LPS-induced sepsis. SAR analysis of synthetic analogs revealed that the presence of an electron-withdrawing substituent on the B ring (**502**, **503**, and **512**) seems to contribute to the highest inhibition of TNF-α production (Figure 11). Further investigation into the possible mechanism revealed that the anti-inflammatory activity of the analog **512** might be correlated with its inhibition on NF-κB and ERK pathway activation [54].

A range of 3,5-bis(arylmethylene)piperidin-4-ones (**520**–**532**: Figure 10) and related *N*-cyclopropyl analogs (**533**–**537**: Figure 10) were prepared and evaluated for their ability to interfere with LPS-induced TNF-α and IL-6 expression using both in vitro and in vivo models. Most of these compounds exhibited significant inhibition of IL-6 expression but almost no activity toward TNF-α levels, except compound **526** (% inhibition = 39.4%). It was observed that the incorporation of various alkyl groups in the piperidone motif (**522**–**526**) improved IL-6 expression inhibition compared to the unsubstituted compound (**521**) (Figure 11). Moreover, if the substituents in the nitrogen atom were substituted by benzyloxy groups (**528**–**532**), more potent IL-6 inhibition was detected (Figure 11). Compound **543** significantly inhibited both cytokines at a concentration of 10 µM. This compound (**533**) was further evaluated for in vivo inhibitory activity and results revealed that it caused a significant and dose-dependent inhibition of cytokine production [55].

In a model of LPS-induced IL-6 and TNF-α release, the inhibitory effects of a series of resveratrol and diarylpentanoid hybrids (**538**–**564**: Figure 10) was evaluated in vitro (using the MTT assay on the normal human hepatic cell line HL-7702) and in vivo [56]. Overall, the curcuminoids with an acyclic C5 bridge were more effective in preventing the expression of IL-6 and TNF-α compared to the control compound, resveratrol, or other curcuminoids with a cyclic C5 bridge (**539**–**543**). Particularly, compounds **459**, **473**, **547**, **550**, and **558** showed significant anti-inflammatory activity in a dose-dependent manner with low toxicity in vitro. Diarylpentanoid **547** decreased LPS-induced TNF-α, IL-6, IL12, and IL-33 mRNA expression. Additionally, **547** significantly protected against LPS-induced acute lung injury in an in vivo mouse model. SAR analysis indicates the influence of different C5 bridges and the relation of the position and number of substituent groups on the aromatic rings with anti-inflammatory activity (Figure 11). Firstly, it was observed that the penta-1,4-dien-3-one linker was more effective in preventing the expression of IL-6 or TNF-α (Figure 11). As for the mono-substituted compounds, the presence of electron-withdrawing substituents (**538** and **544**) was more favorable for activity than the presence of electron-donating groups (**553**), with *meta*-position (**544**) being better than *ortho*-position (**538**) in terms of the phenyl ring. On the other hand, the presence of a hydrophilic group at the *para*-position of the phenyl ring (**554** and **559**) was significantly associated with higher inhibitory activity than the presence of a hydrophobic group (**546**, **549**, and **552**). Additionally, for di- or tri-substituted compounds in the aromatic rings (**547** and **555**–**562**), the introduction of an electron-withdrawing group at the *ortho*-position and an electron-donating group at the *meta*-position resulted in enhanced anti-inflammatory activity, except for compound **561**. The presence of a heterocyclic ring (**565**) also contributed to the anti-inflammatory effect [56].

#### 3.3.3. Regulation of COX and LOX Pathways by Diarylpentanoids

Regulation of the activity of cyclooxygenase (COX) and 5-lipoxygenase (5-LOX), involved in the biosynthesis of prostaglandins (PGs), leukotrienes (LTs), and thromboxanes (Tx), plays an important role in the process of inflammation and associated diseases. Thus, inhibition of the biosynthesis of these inflammatory mediators has been proposed as a potential therapy for different inflammatory disorders.

**Figure 12:**
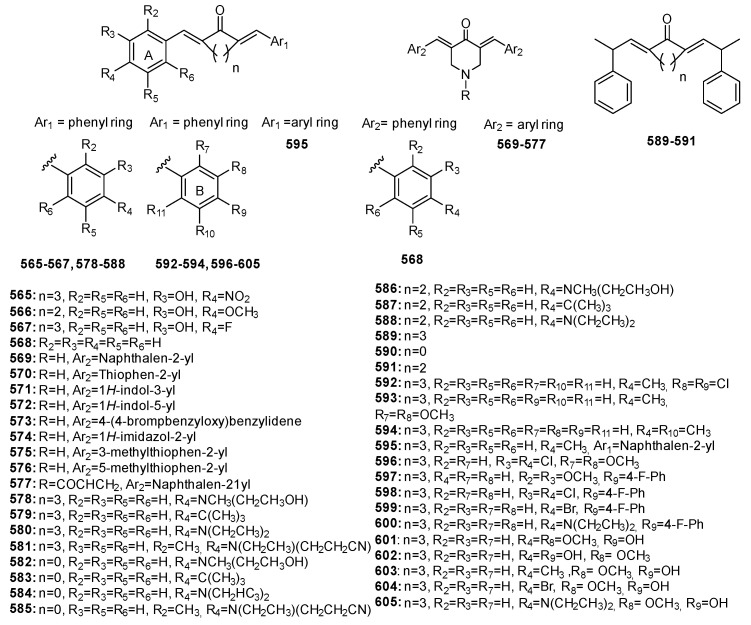
Diarylpentanoids with promising inhibitory effects in COX and LOX pathways.

**Figure 13:**
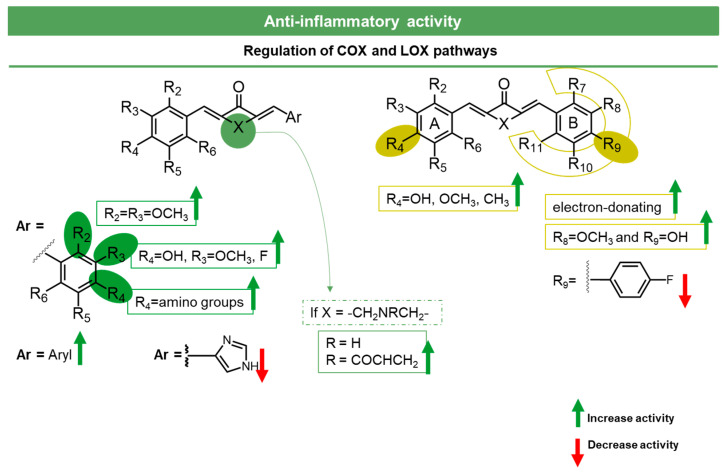
Putative SAR for the effects of diarylpentanoids on the regulation of COX and LOX pathways.

A series of diarylpentanoids with cyclohexanone (**29**, **33**, and **34**: Figure 3; **129**, **131**, and **165**: Figure 6; **379**, **565**, and **567**: Figure 12) or cyclopentanone (**23**: Figure 3; **114**: Erro! A origem da referência não foi encontrada.; and **566**: Figure 12) moieties were tested for their ability to inhibit COX-1 and -2 using an O_2_-consumption assay and the HPLC method, respectively [57]. Generally, compounds inhibited COX-2, except compound **21** that was found to be inactive. In contrast, inhibition of COX-1 was only observed for three compounds with a cyclohexanone moiety (**34**: 27%; **566**: 20%; and **567**: 56%) [57]. Additionally, compound **567**, the most active compound for both isoenzymes, presented IC_50_ values of 39.8 and 23.7 µM (COX-1 and COX-2, respectively), demonstrating it is a less potent inhibitor than curcumin (**1**, COX-1: IC_50_ = 18.8 µM; COX-2: IC_50_ = 15.9 µM) [57]. In general, there is no evidence that modifications to the C5 bridge enhance the activity of the compounds. However, the substitution pattern of the aromatic moiety is of importance. Compounds with *m*-methoxy and *p*-hydroxy groups in phenyl rings (**23** and **34**) and *m*-hydroxy-*p*-fluorophenyl rings (**567**) were the most active (Figure 13) [57].

Diarylpentanoids with phenyl (**568**: Figure 12), naphthyl (**569**: Figure 12, and heterocyclic rings (**570**–**577**: Figure 12) were examined for their anti-inflammatory activity in both in vitro and in vivo assays. Among these compounds, **569** was the most potent LOX inhibitor (IC_50_ = 37 µM), followed by **570**–**572** (IC_50_ = 280-410 µM). From these results, some SAR considerations may be undertaken (Figure 13). The presence of heterocyclic rings is associated with high anti-inflammatory activity (**570**–**572**), except for imidazole rings (**574**). Contrarily, diarylpentanoids with phenyl rings (**568** and **573**) showed low activity. Nevertheless, the presence of methyl groups in compounds with heterocyclic rings (**575** and **576**) appears to be unfavorable for activity. Lastly, the acryloyl group in piperidone (**577**) is tolerated. Docking studies were performed with compound **569** on LOX, and it was observed that allosteric interactions may govern LOX-inhibitor binding [58].

Symmetric cyclic and acyclic diarylpentanoids (**217**, **328**, **351**, **362**, **578**–**591**: Figure 12) were evaluated for their effects on the enzymes of the arachidonic acid (AA) cascade, namely LOX, COX, and microsomal prostaglandin E synthase-1 (mPGES-1), using a standard colorimetric assay, COX inhibitor screening assay kit, and a photometric assay, respectively [59]. The results showed that diarylpentanoids have affinity toward COX-1, with compounds **217**, **578**, **581**, **582**, and **585** being similar or better inhibitors than curcumin (**1**: IC_50_ = 31.31 ± 0.45 µM). In the LOX assay, compounds **217**, **328**, **581**, and **585** (IC_50_ = 30.02–62.86 µM) had similar or better activity than curcumin (**1**: IC_50_ = 57.7 µM). In the study of mPGES-1’s activity, **578**, **580**–**582**, **586**, and **587** showed strong inhibitory effects (IC_50_= 2.4–5.4 µM; curcumin (**1**): IC_50_ = 4.88 µM). The SAR studies revealed that the diethylamine group, at the *para*-position of both phenyl rings, leads to selectivity for COX-1 (**580**, **584**, and **588**), inhibition of LOX (**588**), and strong inhibition of mPGES-1 activity (**580** and **588**) (Figure 13). The presence of *N*-methyl-*N*-(2-hydroxyethyl)-4-amino (**578**, **582**, **584**, and **586**) or 2-methyl-*N*-ethyl-*N*-(2-cyanoethyl)-4-amino (**581** and **585**) groups at the *para*-position of both phenyl rings was associated with strong COX-2, LOX, and mPEGS-1 inhibitory activity (Figure 13). On the other hand, inhibitory activity on COX-1/2 and LOX increased with a methoxy group at the *ortho*- and *meta*-positions (**217**, **362**, and **328**) of the aromatic rings, except for compound **362** which has a similar substitution pattern but contains a cyclopentanone moiety. However, substitution of the phenyl rings with a *para*-*tert*-butyl group produced compounds with lower inhibitory activity toward LOX [59].

Aluwi et al. (2016) synthesized a series of asymmetrical diarylpentanoids (**592**–**605**, Figure 12) and evaluated their effects on PGE2 production in human and murine macrophages stimulated by LPS. Compounds **593**, **594**, **601**, and **602** strongly inhibited the secretion of prostaglandin E2 (PGE2) in RAW 264.7 (IC_50_ = 0.78–12.01 µM) and U937 cells (IC_50_ = 0.92–3.44 µM), with these results being more promising than those obtained for curcumin (**1**). Moreover, **593**, **594**, **601**, and **602** selectively inhibited COX-2 (% inhibition = 22–30%) over COX-1. These results suggested that the presence of electron-donating groups at the *para*-position on the A aromatic ring, as well as in different positions on the B ring, was favorable for COX inhibition (Figure 13). Finally, docking simulations on COX-2 were performed [60]. Results suggested that **601** could preferably target p38a or IKK-b, subsequently down-regulating COX-2 expression and eventually reducing PGE2 production.

### 3.4. Antidiabetic Activity

In 2006, Du et al. evaluated the effect of a series of diarylpentanoids on α-glucosidase inhibition using a spectrophotometric assay [19]. In addition to the previously mentioned activities, compounds **8**, **9**, **22**, **23**, **33**, and **34** (Figure 3), as well as compounds **110**, **111**, **112**–**115**, **120**, **121**, **122**, **128**, **129**–**131**, and **137**–**143** (Figure 6), have also interfered with α-glucosidase inhibition. It was observed that compounds that were tetrahydroxyl-substituted (**111**, **120**, **128**, and **138**: IC_50_ = 1.6–8.2 µM) exhibited much higher inhibitory activity than the positive controls, curcumin (**1**), and acarbose. SAR analysis of all diarylpentanoids (Figure 5) revealed that compounds with 3,5-dimethoxy-4-hydroxy phenyl groups (**110**, **115**, **131**, and **142**: IC_50_ > 100 µM) had lower activity when compared to diarylpentanoids with *p*-hydroxyphenyl rings (**22**, **33**, and **137**: IC_50_ = 32.5–61.3 µM), with compounds with catechol groups (**111**, **120**, **128**, and **138:** IC_50_ = 1.6–8.2 µM) being the most active (Figure 5). Further, activity decreases in the presence of bulky groups, such as *ortho*-*tert*-butyl groups (**9**, **112**, **129**, and **139**: IC_50_ > 100 µM). Kinetic studies showed that **120** was a non-competitive inhibitor of α-glucosidase [19].

Symmetric diarylpentanoid **23** (Figure 3), (2*E*,5*E*)-2,5bis(4-hydroxy-3-methoxybenzylidene), was synthesized and its effect on the blood glucose level and lipid profile of normoglycemic and streptozotocin (STZ)-induced diabetic rats was investigated [61]. In an oral glucose and sucrose tolerance test, treatment with **23** (25 mg/kg) and the standard drug glidenclamide (10 mg/kg) significantly improved glucose tolerance and significantly reduced AUC glucose compared to the NC group. In turn, treatment with **23** (25 mg/kg) and the α-glucosidase inhibitor acarbose (7 mg/kg) exhibited a significant reduction in SG level over the period of 120 min compared to the NC group. Furthermore, treatment with **23** significantly improved sucrose tolerance in normal rats, suggesting that **23** inhibited α-glucosidase. Lipid profile studies also support the antidiabetic efficacy of **23**. These results suggested that *meta*-methoxylated and *para*-hydroxylated positions were important for the potential antidiabetic effect of diarylpentanoids. These data suggested that the potency of the compound is comparable to the standard drug glidenclamide [61].

**Figure 14:**
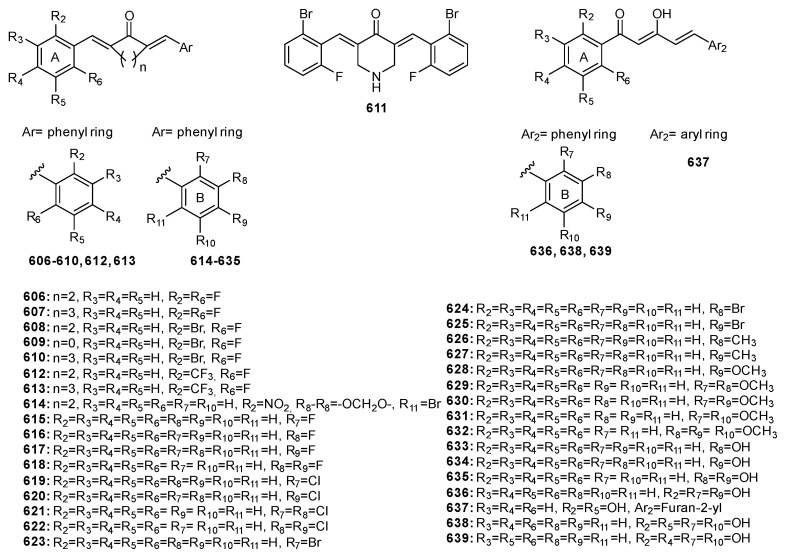
Diarylpentanoids with promising antidiabetic properties.

**Figure 15:**
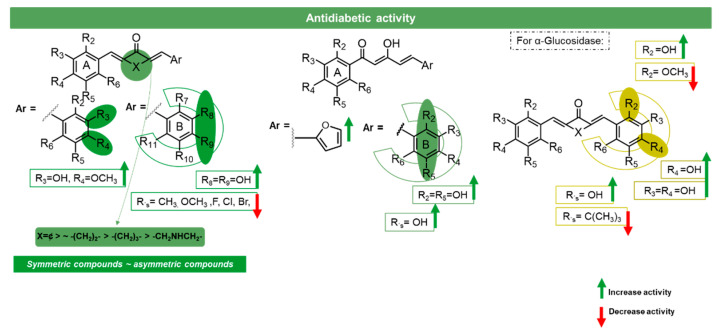
Putative SAR for the antidiabetic activity of diarylpentanoids.

A small library of symmetric diarylpentanoids (**6**: Figure 3; **606**–**613**: Figure 14) with diverse moieties, including 3-oxo-penta-1,4-diene (**6** and **609**), cyclopentanone (**606**, **608**, and **612**), cyclohexanone (**607**, **610**, and **613**), and piperidone (**611**), were synthesized and evaluated for their ability to inhibit 11β-hydroxysteroid dehydrogenase (11β-HSD) with anti-diabetic properties [62]. All tested compounds possessed much higher activity than curcumin (1), exhibiting higher inhibition of 11β-HSD1 in rat testis microsomes (IC_50_ = 407.4–4767 nM) than in human liver microsomes (IC_50_ = 650.5–> 100,000 nM). However, diarylpentanoids weakly inhibited 11β-HSD2. It was verified that compounds **606**, **608**, **609**, and **612** were selective inhibitors of human and rodent 11β-HSD1. These results indicated that the presence of 3-oxo-penta-1,4-diene and cyclopentanone moieties is much more favorable than the presence of cyclohexanone (**107** and **108**) and piperidone moieties (Figure 15). Additionally, compound **612** displayed antidiabetic properties in diabetic mice that were induced through the use of streptozocin and a high-fat diet (STZHFD) [62].

Chen et al. (2017) prepared a new asymmetric diarylpentanoid (**614**: Figure 14) and investigated its effects on hyperglycemia-induced inflammation and fibrosis in NRK-52E and H9C2 cells through a streptozotocin-induced diabetic mouse model. It was observed that compound **514** exerted significant inhibitory effects on hyperglycemia-induced inflammation and fibrosis in NRK-52E and H9C2 cells, with these effects being associated with the inhibition of the P38 and AKT signaling pathway, respectively. Diabetes was induced in mice by a single intraperitoneal injection of freshly prepared STZ (100 mg/kg in citrate buffer, pH = 4.5). The group treated with **614** experienced a slight reduction in blood glucose compared to the STZ-treated group. The **614**-treated mice presented indicators of decreased heart function but the indicators of kidney function did not change [63]. These results demonstrated that symmetry is not a requirement for the antidiabetic potential of diarylpentanoids (Figure 15).

In 2018, other series of asymmetric diarylpentanoids with different C5 bridges (**197**–**199**: Figure 6; **261**, **262**, **265**, **266**, **268**, and **275**: Figure 8; and **615**–**639**: Figure 14) were synthesized by Leong et al. (2018) and were evaluated for their α-glucosidase inhibitory activity. Eleven compounds (**199**, **265**, **268**, **275**, and **633**–**639**) were found to significantly inhibit α -glucosidase, with IC_50_ values between 14.1 and 42.2 µM. Among the compounds with the cyclohexanone moiety, the results suggested that the presence of hydroxyl groups on the B ring is more favorable for activity (**633**–**635**: IC_50_ = 20.3–42.3 µM) than halogen, methyl, and methoxy groups (**615**–**632**) (Figure 15). For the remaining compounds, multiple substitutions with hydroxyl groups on the phenyl ring (**268**, **175**, **638**, and **639**) maintained their importance in α-glucosidase inhibition. Among polyhydroxylated compounds, **638** and **639** were the most potent. Interestingly, the presence of a furanyl moiety (**637**) tends to enhance α-glucosidase inhibitory activity (Figure 15). Additionally, molecular docking analysis was performed, confirming the critical role of both *meta*- and *para*-dihydroxyphenyl moieties, as well as furanyl moieties, as they bound to α-glucosidase active sites in different modes [64]. 

### 3.5. Anti-Hyperuricemic Activity

**Figure 16:**
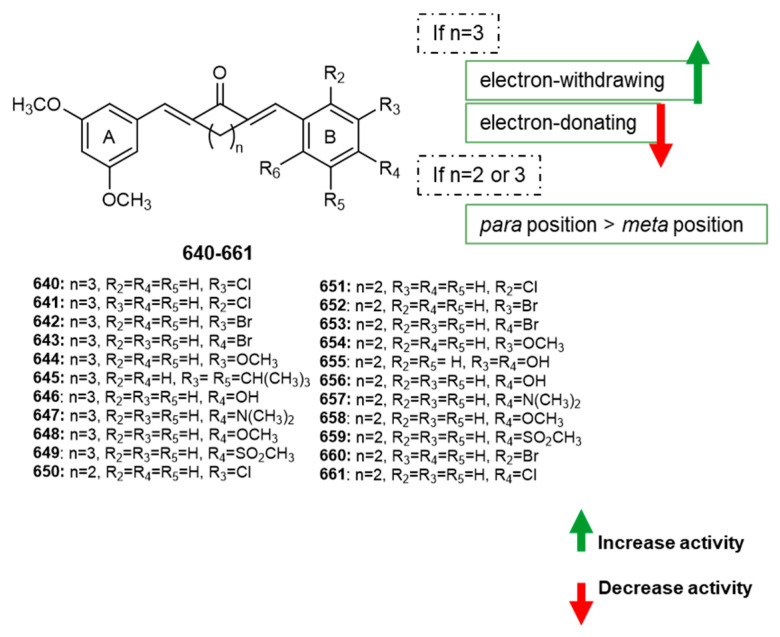
Diarylpentanoids as promising anti-hyperuricemic agents.

A set of asymmetric diarylpentanoids with cyclohexanone and cyclopentanone moieties (**640**–**661**: Figure 16) was synthesized and tested for its effectiveness as an anti-hyperuricemic agent by using hyperuricemic mice models induced with oxonate treatment [65]. Generally, compounds exhibited remarkable anti-hyperuricemia activity, especially **643** which showed the most potent inhibitory activity (uric acid lowering activity of 92.4%), comparable to allopurinol (148.32%) and benzbromarone (99.7%), used as positive controls. Compounds **642**, **643**, and **649** displayed the most potent uricosuric activity. For the compounds with a cyclohexanone moiety (**640**–**649**), the electron-withdrawing substituents on the phenyl ring (**640**–**644**) input substantial effects compared to other compounds with electron-donating substituents (**645**–**648**) (Figure 16). Analyzing all compounds, those with substitution at the *para*-position (**643**, **648**, **653**, and **661**) showed better activity than those with substitution at the *meta*-position (**642**, **644**, **650**, and **652**) (Figure 16). Xanthine oxidase (XOD) and urate transporter 1 (URAT1) inhibitory activities were studied for the most promising compounds (**640**–**643**, **649**–**662**, and **659**). Compounds **643** and **649** showed the most potent (IC_50_ = 5.7 nM and 10.8 nM) XOD inhibitory activity and **643** displayed the most potent URAT1 inhibitory activity (38.2%). A docking study was performed to elucidate the potent XOD inhibition exhibited by **643**. Interestingly, compound **643** may serve as a tool for the further design of dual anti-hyperuricemic drugs targeting both XOD and URAT1 [65].

### 3.6. Neuroprotector Activity

Among neurodegenerative disorders, Alzheimer’s disease (AD) is one of the most common, leading to progressive and irreversible neurodegeneration characterized by permanent memory loss, cognitive impairment, disorientation, confusion, and language deficits [66]. Acetylcholinesterase (AChE) and butyrylcholinesterase (BChE) are important enzymes that catalyze the degradation of acetylcholine, an important neurotransmitter involved in memory and cognition. Targeting AChE and BChE may be one of the most promising approaches for treating AD [67,68]. Interestingly, it was reported that BChE activity is dramatically increased when AChE is inhibited, which implies that dual inhibitors are a better choice for treating some diseases.

**Figure 17:**
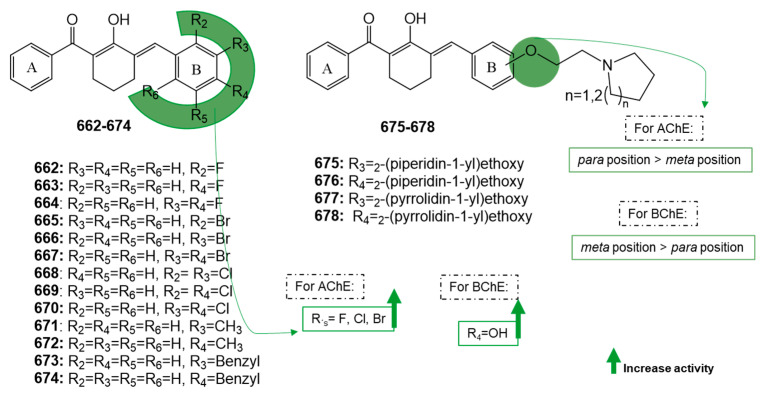
Diarylpentanoids as promising neuroprotector agents.

A series of 2-benzoyl-6-benzylidenecyclohexanone analogs (**192**, **194**, **196**, **199**–**201**, **207**–**208**, and **210**–**212**: Figure 6; **662**–**678**: Figure 17) were synthesized and tested for their anti-AChE and BChE activity [69]. Most of the compounds (**192**, **194**, **196**, **200**, **201**, **210**–**212**, **662**–**678**) were found to inhibit AChE activity (IC_50_ = 1.6–9.8 µM), and compounds **199**, **207**, **208**, and **675**–**678** showed reasonable inhibition of BChE activity (IC_50_ = 0.6–10.6 µM). Interestingly, compounds **675**–**678** have been identified as lead compounds due to their high inhibition of both AChE (IC_50_ = 1.6–3.1 µM) and BChE (IC_50_ = 0.6–2.7 µM). The Lineweaver–Burk plots and docking results suggest that compounds **676** and **677** could simultaneously bind to the PAS and CAS regions of the enzyme. ADMET analysis further confirmed the therapeutic potential of both compounds based on their high BBB-penetration [69]. Some SAR conclusions can be inferred (Figure 17). Particularly, the presence of heterocyclic amines (piperidine **675** and **676** or pyrrolidine **677** and **678**) as substituents in the B ring is particularly important for AChE and BChE inhibitory activity, with *para*-substitution being preferable (**676** and **678**) to *meta*-substitution (**675** and **677**) for AChE inhibitory activity, and *meta*-substitution being preferable to *para*-substitution for BChE inhibition. Another contributing factor to BChE inhibitory activity was the presence of *para*-hydroxyl groups at the B ring (**99**, **207**, and **208**: IC_50_ = 4.7–10.6 µM). For AChE inhibitory activity, other aryl (**190** and **191**) and halogenated phenyl groups (**194**, **196**, and **662**–**670**) are tolerated (Figure 17). 

### 3.7. Interference with Diverse Biochemical Targets

In addition, a total of 83 diarylpentanoids have also been described for their ability to interfere with several proteins, namely enzymes and channels. 

**Figure 18:**
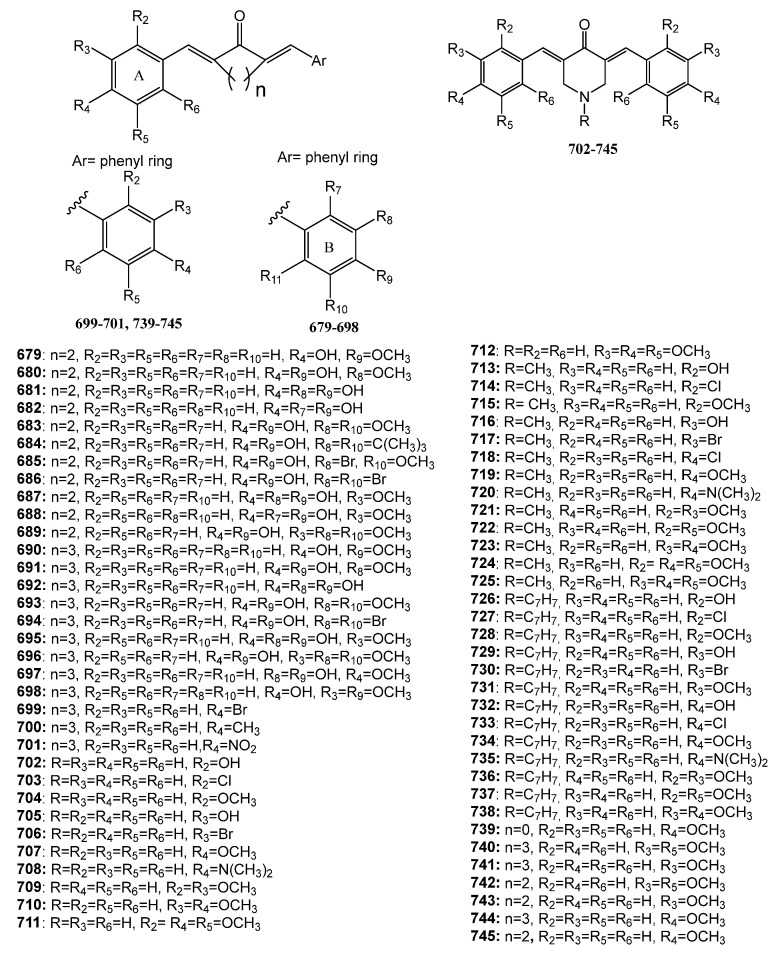
Diarylpentanoids described for their ability to interfere with several proteins.

**Figure 19:**
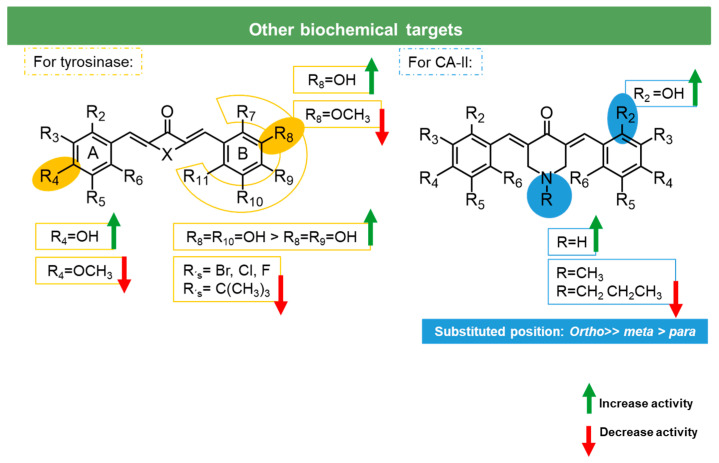
Putative SAR diarylpentanoids described for their ability to interfere with several proteins.

The interference of diarylpentanoids in relation to the tyrosinase enzyme has also been reported. Tyrosinase is an oxidoreductase enzyme (EC 1.14.18.1) involved in several biosynthetic processes. Jiang et al. (2013) synthesized asymmetric diarylpentanoids (**679**–**698**: Figure 18) and studied their ability to inhibit tyrosinase activity [70]. Almost all of the tested compounds displayed inhibitory activities against mushroom tyrosinase, with compounds with dihydroxyl groups on the B ring (**681**, **682**, **687**, **688**, **692**, and **695**) being the most promising inhibitors (IC_50_ = 1.74~16.74 µM). Among these, **686** was the strongest inhibitor, with an IC_50_ value of 1.74 µM which was about sixteen-fold and six-fold lower than that of the controls kojic acid (IC_50_ = 28.59 µM) and 4-butylresorcinol (IC_50_ = 11.27 µM), respectively [70]. From these results, some SAR considerations may be undertaken (Figure 19). Compounds with a *para*-hydroxyl group (**692**: IC_50_ = 4.64 µM) on the A ring were more potent than the analogs with *para*-methoxy groups in the same ring (**697**: IC_50_ = 86.92 µM). Similar results were observed for compounds with a *meta*-hydroxyl group (**681**: IC_50_ = 6.78 µM) on the B ring compared to *meta*-methoxy groups in the same ring (**680**: IC_50_ = 56.64 µM). Additionally, the presence of halogenated groups (**686**, **685**, and **694**: IC_50_ > 170 µM) as well as bulky *tert*-butyl substituents (**684**: IC_50_ = 168.36 µM), significantly decreased tyrosinase activity (Figure 19). Compounds containing a resorcinol moiety (**441** and **688**) were more active than those containing a catechol group (**681**, **687**, and **695**). The preliminary evaluation of acute toxicity showed that **682** and **688** were non-toxic in mice at 1.200 mg/kg [70]. 

Alkaline and acid phosphatase are enzymes of hydrolase classes (EC 3.1.3.1 and EC 3.1.3.2, respectively) that are found in all living tissues and are involved with the removal of the phosphate from protein and other molecules. These enzymes are used as diagnostic tools in various pathological conditions [71,72]. In 2014, Singh et al. tested symmetric diarylpentanoids with a cyclic C5 bridge (**28**: Figure 3; **165**–**167**: Figure 6; **351**: Figure 10; and **699**–**701**: Figure 18) for their ability to interact with acid and alkaline phosphatases [73]. Although compounds have presented inhibitory activity (% of inhibition ~40–75%) with alkaline phosphatases, an activating effect was detected for acid phosphatases (% activation ~25–60%). Additionally, molecular modeling and docking of these compounds into alkaline phosphatase using iGemdock was performed. All compounds showed greater affinity to alkaline phosphatase than the control levamisole. All compounds inhibited enzyme activity in a competitive manner, except compound **700**, whose inhibitory effect was shown to be non-competitive [73]. 

Carbonic anhydrase II (CA-II) is one of sixteen forms of human α-carbonic anhydrases, an enzyme of the lyase class (E.C.4.2.1.1). The isozyme CA-II is known to be one of the most efficient in CO_2_ hydration. Several diseases are associated with CA deregulation, such as glaucoma, hypertension, edema, epilepsy, and cancer; thus, the development of CA inhibitors has been a strategy for the treatment of CA-related disorders [74]. Aditama et al. (2017) described in vitro and in silico studies of diarylpentanoids (**98**: Figure 6; **702**–**738**: Figure 18) as inhibitors of CA-II. The authors reported that all compounds exhibited low or moderate inhibition, except for **702** that showed high inhibitory activity (IC_50_ = 7.92 µM). The presence of hydroxyl groups at the *ortho*-position (**702**) of both aromatic rings is associated with high inhibition against CA-II compared to the presence of hydroxyl groups at the *meta*- (**705**, **716**, and **729**) or *para*-positions (**98** and **732**), as well as compared to the presence of methoxy groups (Figure 19). Additionally, the substitutions on the 4-piperidone core seemed to interfere with inhibitory activity, since compounds **713** and **726** with the *N*-methyl-4-piperidone and *N*-propyl-4-piperidone moiety, respectively, exhibited contrasting results to **702**, which possesses an unsubstituted 4-piperidone core (Figure 19). Lastly, molecular docking analysis revealed that compound **702** formed hydrogen interactions with Thr199, Thr200, and Gln92 at the active site of CA-II [75].

The transient receptor potential (TRP) channels of ankyrin type-1 (TRPA1) and vanilloid type-1 (TRPV1), which belong respectively to the TRPA and TRPV subfamilies of the large TRP cation channel family, play numerous roles in many physiological and pathophysiological processes and are involved in the perception of nociceptive and inflammatory pain via sensory nerve activation. In 2017, the ability to interact with channels TRPA1, TRPM8, and TRPV1 for a series of symmetric diarylpentanoids (**3**, **9**, **13**, **23**, and **28**: Figure 3; **163**, **165**, and **167**: Figure 6; **327**, **334**, and **346**: Figure 10; and **739**–**745**, Figure 18) was described by Nalli et al. All tested compounds acted as good modulators of TRPA1 channels (IC_50_ = 0.27–38.3 µM), except compound **742** (IC_50_ > 50 µM). However, only few diarylpentanoids were able to significantly activate TRPM8 (**334**: IC_50_ = 3.3 µM) and TRPV1 (**3**: IC_50_ = 4.1 µM; **334**: IC_50_ = 5.2 µM) channels [76].

## 4. Conclusions

Diarylpentanoids are important scaffolds in medicinal chemistry which are known for their great variety of biological activities. In this review, a total of 745 diarylpentanoids with a wide range of biological activities, beyond just antitumor activity, have been described. The most commonly employed method for the synthesis of symmetric diarylpentanoids is Claisen–Schmidt condensation. However, other methods have been reported to obtain asymmetric diarylpentanoids.

**Figure 20:**
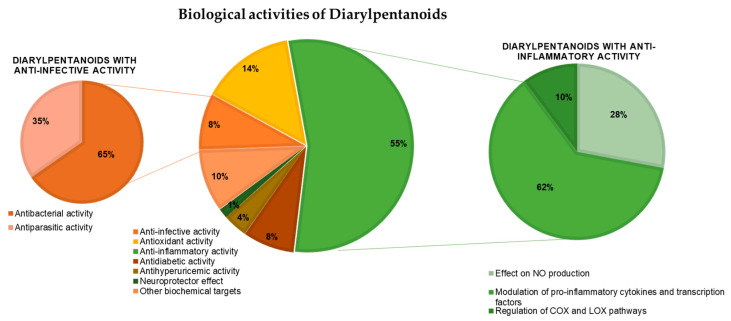
Distribution of the diarylpentanoids according to biological activity.

Among biological activities, anti-inflammatory activity has been the most studied, with the modulation of pro-inflammatory cytokines and transcription factors being the most reported (Figure 20). Considering the diarylpentanoids with anti-infective activity, it was observed that only symmetric diarylpentanoids exhibited antibacterial activity, and only asymmetric acyclic compounds were found to be antiparasitic agents. Anti-hyperuricemic and neuroprotector activities were the least reported, and only asymmetric diarylpentanoids could be found in the literature. On the other hand, for antioxidant, anti-inflammatory, and antidiabetic activities, both symmetric and asymmetric diarylpentanoids have been reported.

This review shows that the diarylpentanoid scaffold still gathers a lot of attention, not only from a structural variety point of view, but also because of pharmacological and biological properties. Information about the most relevant molecular features of the biological activities provided in this manuscript can be useful for designing new bioactive diarylpentanoids with improved therapeutic activity against diverse diseases.

## Data Availability

Not applicable.

## Supplementary Materials

The following supporting information can be downloaded at: https://www.mdpi.com/article/10.3390/molecules27196340/s1, Table S1: Diarylpentanoids with biological effects beyond antitumor activity; References citation of [77].

## Abbreviations

AA	Arachidonic acid
ABTS^+^	2,2’-azinobis(3-ethylbenzothiazoline-6-sulfonicacid) radical cation assay
AChE	Acetylcholinesterase
AD	Alzheimer’s disease
ADMET	Absorption, distribution, metabolism, excretion, toxicity
AP-1	Activator protein 1
BChE	Butyrylcholinesterase
CA-II	Carbonic anhydrase II
COX	Cyclooxygenase
DPPH	Diphenylpicrylhydrazyl assay
FRAP	Antioxidant power assay
IC_50_	Half-maximal inhibitory concentration
IFN-γ	Interferon-gamma
iNOS	Nitric oxide synthase
IZ	Inhibition zone
LOX	Lipoxygenase
LPS	Lipopolysaccharide
LTs	Leukotrienes
NET	O^2−^• radical scavenging assay
NF-κB	Nuclear factor kappa-light-chain-enhancer of activated B cells
pdb	Protein data bank
PMNs	Polymorphonuclear leukocytes
PGs	Prostaglandins
QSAR	Quantitative structure–activity relationship
SAR	Structure–activity relationship
STZHFD	Streptozocin and high-fat diet
TNF-α	Tumor necrosis factor-alpha
TRAP	ROO• radical scavenging assay
TRP	Transient receptor potential
TRPA1	Transient receptor potential channels of ankyrin type-1
TRPV1	Transient receptor potential channels of vanilloid type-1
Tx	Thromboxanes
URAT1	Urate transporter 1
XOD	Xanthine oxidase
11β-HSD	11β-hydroxysteroid dehydrogenase
