# Supplementary material for: Bioactive Diarylpentanoids: Insights into the Biological Effects beyond Antitumor Activity and Structure–Activity Relationships
Source: Molecules. 2022 Sep 26;27(19):6340. doi: 10.3390/molecules27196340 (PMC9572019; doi:10.3390/molecules27196340)
Supplement: Supplementary file 1 [file molecules-27-06340-s001.zip › molecules-1917328-supplementary.pdf]

Supporting Information

Bioactive diarylpentanoids: insights into the biological effects beyond antitumor activity and structure-activity relationships

Joana Moreira[1,2], Lucilia Saraiva[3], Madalena M. Pinto[1,2,*], Honorina Cidade[1,2,*]

1 Laboratory of Organic and Pharmaceutical Chemistry, Department of Chemical Sciences, Faculty of Pharmacy, University of Porto, Rua de Jorge Viterbo Ferreira 228, 4050-313 Porto, Portugal

2 Interdisciplinary Centre of Marine and Environmental Research (CIIMAR), University of Porto, Edifício do Terminal de Cruzeiros do Porto de Leixões, Avenida General Norton de Matos, S/N, 4450-208 Matosinhos, Portugal

3 LAQV/REQUIMTE, Laboratory of Microbiology, Department of Biological Sciences, Faculty of Pharmacy, University of Porto, Rua Jorge Viterbo Ferreira, 228, 4050-313 Porto, Portugal

* Correspondence: madalena@ff.up.pt (M.M.P.); hcidade@ff.up.pt (H.C.);

Tel.: +351-220428692 (M.M.P.); +351-220428688 (H.C.)

Table S1. Diarylpentanoids with biological effects beyond antitumor activity.

ANTI-INFECTIVE ACTIVITY			
**Antibacterial activity**			
**Compounds**		**Reference Drug**	**Ref.**
**2**	IZ= 9, 10 mm (*E. cloacae, E. coli*)	CURCUMIN<br>IZ= 11, 10 mm (*E. cloacae, E. coli)*<br>AMPICILIN<br>IZ= 31 mm (*E. coli)*	[1]
**3**	IZ= 10, 11, 10, 11, 13, 10 mm (*S. aureus, Micrococus, S. saprophyticus, S. epidermidis, E. cloacae, E. coli*)	CURCUMIN<br>IZ= 11, 9, 10, 11, 11, 10 mm (*S. aureus, Micrococus, S. saprophyticus, S. epidermidis, E. cloacae, E. coli)*<br>AMPICILIN<br>IZ= 30, 23, 18, 18, 31 mm (*S. aureus, Micrococus, S. saprophyticus, S. epidermidis, E. coli)*	[1]
**4**	IZ= 14, 10, 11 mm (*S. aureus, Micrococus, E. cloacae*)	CURCUMIN<br>IZ= 11, 9, 11 mm (*S. aureus, Micrococus, S. saprophyticus, E. cloacae)*<br>AMPICILIN<br>IZ= 30, 23 mm (*S. aureus, Micrococus)*	[1]
**5**	IZ= 10 mm (*E. cloacae*)	CURCUMIN<br>IZ= 11 mm (*E. cloacae*)	[1]
**6**	IZ= 10, 15, 12, 11 mm (*Micrococus, S. epidermidis, E. cloacae, E. coli*)	CURCUMIN<br>IZ= 9, 11, 11, 10 mm (*Micrococus, S. epidermidis, E. cloacae, E. coli)*<br>AMPICILIN<br>IZ= 23, 18, 31 mm (*Micrococus, S. epidermidis, E. coli)*	[1]
**7**	IZ= 11, 10 mm (*Micrococus, E. cloacae*)	CURCUMIN<br>IZ= 9, 11 mm (*Micrococus, E. cloacae*)<br>AMPICILIN<br>IZ= 23 mm (*Micrococus)*	[1]
**8**	IZ= 17, 14, 13, 16, 11, 12, 9 mm (*S. aureus, Micrococus, S. saprophyticus, S. epidermidis, E. cloacae, Enterococus, E. coli*)	CURCUMIN<br>IZ= 11, 9, 10, 11, 11, 10, 10 mm (*S. aureus, Micrococus, S. saprophyticus, S. epidermidis, E. cloacae, Enterococus, E. coli)*<br>AMPICILIN<br>IZ= 30, 23, 18, 18, 23, 31 mm (*S. aureus, Micrococus, S. saprophyticus, S. epidermidis, Enterococus, E. coli)*	[1]
**9**	IZ= 15, 13, 16, 15, 12, 10, 10 mm (*S. aureus, Micrococos, S. saprophyticus, S. epidermidis, E. cloacae, Enterococus, E. coli*)	CURCUMIN<br>IZ= 11, 9, 10, 11, 11, 10, 10 mm (*S. aureus, Micrococus, S. saprophyticus, S. epidermidis, E. cloacae, Enterococus, E. coli)*<br>AMPICILIN<br>IZ= 30, 23, 18, 18, 23, 31 mm (*S. aureus, Micrococus, S. saprophyticus, S. epidermidis, Enterococus, E. coli)*	[1]

**10**	IZ=9, 9 mm (*E. cloacae, E. coli*)	CURCUMIN<br>IZ= 11, 10 mm (*E. cloacae, E. coli)*<br>AMPICILIN<br>IZ= 31 mm (*E. coli)*	[1]
**11**	IZ= 18, 13, 15, 13, 10, 9, 11 mm (*S. aureus, Micrococus, S.saprophyticus, S. epidermidis, E. cloacae,Enterococus, E. coli*)	CURCUMIN<br>IZ= 11, 9, 10, 11, 11, 10, 10 mm (*S. aureus, Micrococus, S. saprophyticus, S. epidermidis, E. cloacae, Enterococus, E. coli)*<br>AMPICILIN<br>IZ= 30, 23, 18, 18, 23, 31 mm (*S. aureus, Micrococus, S. saprophyticus, S. epidermidis, Enterococus, E. coli)*	[1]
**12**	IZ= 14, 10 mm (*Micrococus, E. cloacae*)	CURCUMIN<br>IZ= 9, 11 mm (*Micrococus, E. cloacae*)<br>AMPICILIN<br>IZ= 23 mm (*Micrococus)*	[1]
**13**	IZ= 10, 9 mm (*E. cloacae, E. coli*)	CURCUMIN<br>IZ= 11, 10 mm (*E. cloacae, E. coli)*<br>AMPICILIN<br>IZ= 31 mm (*E. coli)*	[1]
**14**	IZ= 10, 10, 9 mm (*Micrococus, E. cloacae, Enterococus*)	CURCUMIN<br>IZ= 9, 11, 10 mm (*Micrococus, E. cloacae, Enterococus)*<br>AMPICILIN<br>IZ= 23, 23 mm (*Micrococus, Enterococus)*	[1]
**15**	IZ= 12, 9 mm (*Micrococus, E. cloacae*)	CURCUMIN<br>IZ= 9, 11 mm (*Micrococus, E. cloacae*)<br>AMPICILIN<br>IZ= 23 mm (*Micrococus)*	[1]
**16**	IZ= 10 mm (*E. cloacae*)	CURCUMIN<br>IZ= 11 mm (*E. cloacae*)	[1]
**17**	IZ= 13 mm (*E. cloacae*)	CURCUMIN<br>IZ= 11 mm (*E. cloacae*)	[1]
**18**	IZ= 11 mm (*E. cloacae*)	CURCUMIN<br>IZ= 11 mm (*E. cloacae*)	[1]
**19**	IZ= 12, 10 mm (*Micrococus, E. cloacae*)	CURCUMIN<br>IZ= 9, 11 mm (*Micrococus, E. cloacae*)<br>AMPICILIN<br>IZ= 23 mm (*Micrococus)*	[1]
**20**	IZ= 11 mm (*E. cloacae*)	CURCUMIN<br>IZ= 11 mm (*E. cloacae*)	[1]
**21**	IZ= 20, 17, 17, 18, 13, 17, 14 mm (*S. aureus, Micrococus, S.saprophyticus, S. epidermidis, E. cloacae,Enterococus, E. coli*)	CURCUMIN<br>IZ= 11, 9, 10, 11, 11, 10, 10 mm (*S. aureus, Micrococus, S. saprophyticus, S. epidermidis, E. cloacae, Enterococus, E. coli)*<br>AMPICILIN<br>IZ= 30, 23, 18, 18, 23, 31 mm (*S. aureus, Micrococus, S. saprophyticus, S. epidermidis, Enterococus, E. coli)*	[1]
**22**	IZ= 12, 10 mm (*Micrococus, E. cloacae*)	CURCUMIN<br>IZ= 9, 11 mm (*Micrococus, E. cloacae*)<br>AMPICILIN	[1]

		IZ= 23 mm (*Micrococus)*	
**23**	IZ= 12, 12, 10, 10, 11 mm (*S. aureus, Micrococus, S. saprophyticus, S. epidermidis, E. cloacae*)	CURCUMIN<br>IZ= 11, 9, 10, 11, 11 mm (*S. aureus, Micrococus, S. saprophyticus, S. epidermidis, E. cloacae)*<br>AMPICILIN<br>IZ= 30, 23, 18, 18 mm (*S. aureus, Micrococus, S. saprophyticus, S. epidermidis)*	[1]
**24**	IZ= 10 mm (*E. cloacae*)	CURCUMIN<br>IZ= 11 mm (*E. cloacae*)	[1]
**25**	IZ= 11 mm (*E. cloacae*)	CURCUMIN<br>IZ= 11 mm (*E. cloacae*)	[1]
**26**	IZ= 12, 10, 10, 9, 10 mm (*S. aureus, Micrococus, S. saprophyticus, S. epidermidis, E. cloacae*)	CURCUMIN<br>IZ= 11, 9, 10, 11, 11, 10 mm (*S. aureus, Micrococus, S. saprophyticus, S. epidermidis, E. cloacae, Enterococus)*<br>AMPICILIN<br>IZ= 30, 23, 18, 18, 23 mm(*S. aureus, Micrococus, S. saprophyticus, S. epidermidis, Enterococus)*	[1]
**27**	IZ= 10, 10 mm (*Micrococus, E. cloacae*)	CURCUMIN<br>IZ= 9, 11 mm (*Micrococus, E. cloacae*)<br>AMPICILIN<br>IZ= 23 mm (*Micrococus)*	[1]
**28**	IZ= 8, 9 mm (*Micrococus, E. cloacae*)	CURCUMIN<br>IZ= 9, 11 mm (*Micrococus, E. cloacae*)<br>AMPICILIN<br>IZ= 23 mm (*Micrococus)*	[1]
**29**	IZ= 9, 10 mm (*Micrococus, E. cloacae*)	CURCUMIN<br>IZ= 11 mm (*E. cloacae*)	[1]
**31**	IZ= 12 mm, *S. epidermidis,*	CURCUMIN<br>IZ= 11 mm (*S. epidermidis)*<br>AMPICILIN<br>IZ= 18 mm (*S. epidermidis)*	[1]
**32**	IZ= 11 mm (*E. coli*)	CURCUMIN<br>IZ= 10 mm (*E. coli)*<br>AMPICILIN<br>IZ= 31 mm (*E. coli)*	[1]
**33**	IZ= 9 mm (*E. coli*)	CURCUMIN<br>IZ= 10 mm (*E. coli)*<br>AMPICILIN<br>IZ= 31 mm (*E. coli)*	[1]
**34**	IZ= 11, 13, 10, 13, 10 mm (*S. aureus, Micrococus S. saprophyticus, S. epidermidis, E. cloacae*)	CURCUMIN<br>IZ= 11, 9, 10, 11, 11, 10 mm (*S. aureus, Micrococus, S. saprophyticus, S. epidermidis, E. cloacae, Enterococus)*<br>AMPICILIN<br>IZ= 30, 23, 18, 18, 23 mm (*S. aureus, Micrococus, S. saprophyticus, S. epidermidis, Enterococus)*	[1]
**35**	IZ= 10 mm (*E. cloacae*)	CURCUMIN<br>IZ= 11 mm (*E. cloacae*)	[1]

**36**	IZ= 15, 14, 21, 13, 11, 10, 11 mm (*S. aureus, Micrococus, S. saprophyticus, S. epidermidis, E. cloacae, Enterococus, E. coli*)	CURCUMIN<br>IZ= 11, 9, 10, 11, 11, 10, 10 mm (*S. aureus, Micrococus, S. saprophyticus, S. epidermidis, E. cloacae, Enterococus, E. coli)*<br>AMPICILIN<br>IZ= 30, 23, 18, 18, 23, 31 mm (*S. aureus, Micrococus, S. saprophyticus, S. epidermidis, Enterococus, E. coli)*	[1]
**37**	IZ = 13, 14, 12 mm (*S. typhi, V. cholera, S. aureus*)	CURCUMIN<br>IZ = 9, 14, 12 mm (*S. typhi, V. cholera, S. aureus*)<br>PENCILLIN<br>IZ = 10, 14, 14 mm (*S. typhi, V. cholera, S. aureus*)	[2]
**38**	IZ = 10, 8, 7 mm (*S. typhi, V. cholera, S. aureus*)	CURCUMIN<br>IZ = 9, 14, 12 mm (*S. typhi, V. cholera, S. aureus*)<br>PENCILLIN<br>IZ = 10, 14, 14 mm (*S. typhi, V. cholera, S. aureus*)	[2]
**39**	IZ = 14, 9, 8 mm (*S. typhi, E. coli, S. aureus*)	CURCUMIN<br>IZ = 9, 9, 12 mm (*S. typhi, E. coli, S. aureus*)<br>PENCILLIN<br>IZ = 10, 16, 14 mm (*S. typhi, E. coli, S. aureus*)	[2]
**40**	IZ = 14, 12, 8, 10 mm (*S. typhi, V. cholera, E. coli, S. aureus*)	CURCUMIN<br>IZ = 9, 14, 9, 12 mm (*S. typhi, V. cholera, E. coli, S. aureus*)<br>PENCILLIN<br>IZ = 10, 14, 16, 14 mm (*S. typhi, V. cholera, E. coli, S. aureus*)	[2]
**41**	IZ = 8, 10 mm (*V. cholera, S. aureus*)	CURCUMIN<br>IZ =14, 12 mm (*V. cholera, S. aureus*)<br>PENCILLIN<br>IZ = 14, 14 mm (*V. cholera, S.aureus*)	[2]
**42**	IZ = 8mm (*V. cholera*)	CURCUMIN<br>IZ = 14 mm (*V. cholera*)<br>PENCILLIN<br>IZ = 14 mm (*V. cholera*)	[2]
**43**	IZ = 16, 15, 7, 12 mm (*S. typhi, V. cholera, E. coli, S. aureus*)	CURCUMIN<br>IZ = 9, 14, 9, 12 mm (*S. typhi, V. cholera, E. coli, S. aureus*)<br>PENCILLIN<br>IZ = 10, 14, 16, 14 mm (*S. typhi, V. cholera, E. coli, S. aureus*)	[2]
**44**	IZ = 16, 10, 7, 8 mm (*S. typhi, V. cholera, E. coli, S. aureus*)	CURCUMIN<br>IZ = 9, 14, 9, 12 mm (*S. typhi, V. cholera, E. coli, S. aureus*)<br>PENCILLIN<br>IZ = 10, 14, 16, 14 mm (*S. typhi, V. cholera, E. coli, S. aureus*)	[2]

**45**	IZ = 14, 8, 8, 8 mm (*S. typhi, V. cholera, E. coli, S. aureus*)	CURCUMIN<br>IZ = 9, 14, 9, 12 mm (*S. typhi, V. cholera, E. coli, S. aureus*)<br>PENCILLIN<br>IZ = 10, 14, 16, 14 mm (*S. typhi, V. cholera, E. coli, S. aureus*)	[2]
**46**	IZ = 8, 8 mm (*V. cholera, E. coli*)	CURCUMIN<br>IZ = 14, 9 mm (*V. cholera, E. coli*)<br>PENCILLIN<br>IZ = 14, 16 mm (*V. cholera, E. coli*)	[2]
**47**	IZ = 12, 10, 8, 8 mm (*S. typhi, V. cholera, E. coli, S. aureus*)	CURCUMIN<br>IZ = 9, 14, 9, 12 mm (*S. typhi, V. cholera, E. coli, S. aureus*)<br>PENCILLIN<br>IZ = 10, 14, 16, 14 mm (*S. typhi, V. cholera, E. coli, S. aureus*)	[2]
**48**	IZ = 20, 18, 9, 10 mm (*S. typhi, V. cholera, E. coli, S. aureus*)	CURCUMIN<br>IZ = 9, 14, 9, 12 mm (*S. typhi, V. cholera, E. coli, S. aureus*)<br>PENCILLIN<br>IZ = 10, 14, 16, 14 mm (*S. typhi, V. cholera, E. coli, S. aureus*)	[2]
**49**	IZ = 12, 14, 8, 9 mm (*S. typhi, V. cholera, E. coli, S. aureus*)	CURCUMIN<br>IZ = 9, 14, 9, 12 mm (*S. typhi, V. cholera, E. coli, S. aureus*)<br>PENCILLIN<br>IZ = 10, 14, 16, 14 mm (*S. typhi, V. cholera, E. coli, S. aureus*)	[2]
**50**	IZ = 12, 14, 8, 8 mm (*S. typhi, V. cholera, E. coli, S. aureus*)	CURCUMIN<br>IZ = 9, 14, 9, 12 mm (*S. typhi, V. cholera, E. coli, S. aureus*)<br>PENCILLIN<br>IZ = 10, 14, 16, 14 mm (*S. typhi, V. cholera, E. coli, S. aureus*)	[2]
**51**	MIC reduction = 2-fold (*E. faecalis* B3/101)	VANCOMYCIN<br>MIC = 707 μM / 1024 μg/mL (*E. faecalis* B3/101)	[3]
**52**	MIC reduction = 2-fold (*E. coli* SA/2)	CEFOTAXIME<br>MIC = 1134-562 μM / 512-256 μg/mL (*E. coli* SA/2)	[3]
**53**	MIC reduction = 2-fold (*E. coli* SA/2)	CEFOTAXIME<br>MIC = 1134-562 μM / 512-256 μg/mL (*E. coli* SA/2)	[3]
**54**	MIC reduction = 2-fold (*E. coli* SA/2)	CEFOTAXIME<br>MIC = 1134-562 μM / 512-256 μg/mL (*E. coli* SA/2)	[3]
**55**	MIC reduction = 2-fold (*E. faecalis* B3/101)	VANCOMYCIN<br>MIC = 707 μM / 1024 μg/mL (*E. faecalis* B3/101)	[3]
**56**	MIC reduction = 2-fold (*E. faecalis* B3/101)	VANCOMYCIN<br>MIC = 707 μM / 1024 μg/mL (*E. faecalis* B3/101)	[3]

**57**	MIC reduction = 4-fold (*E. faecalis* B3/101)	VANCOMYCIN<br>MIC = 707 μM / 1024 μg/mL (*E. faecalis* B3/101)	[3]
**58**	MIC reduction = 2-fold (*E. coli* SA/2)<br>MIC reduction = 2-fold (*E. faecalis* B3/101)	CEFOTAXIME<br>MIC = 1134-562 μM / 512-256 μg/mL (*E. coli* SA/2)<br>VANCOMYCIN<br>MIC = 707 μM / 1024 μg/mL (*E. faecalis* B3/101)	[3]
**59**	MIC reduction = 8-fold (*E. coli* SA/2)	CEFOTAXIME<br>MIC = 1134-562 μM / 512-256 μg/mL (*E. coli* SA/2)	[3]
**60**	MIC reduction = 8-fold (*E. coli* SA/2)	CEFOTAXIME<br>MIC = 1134-562 μM / 512-256 μg/mL (*E. coli* SA/2)	[3]
**61**	MIC reduction = 2-fold (*E. coli* SA/2)<br>MIC reduction = 2-fold (*E. faecalis* B3/101)	CEFOTAXIME<br>MIC = 1134-562 μM / 512-256 μg/mL (*E. coli* SA/2)<br>VANCOMYCIN<br>MIC = 707 μM / 1024 μg/mL (*E. faecalis* B3/101)	[3]
**62**	MIC reduction = 2-fold (*E. coli* SA/2)	CEFOTAXIME<br>MIC = 1134-562 μM / 512-256 μg/mL (*E. coli* SA/2)	[3]
**63**	MIC reduction = 16-fold (*E. coli* SA/2)<br>MIC reduction = 32-fold (*E. faecalis* B3/101)	CEFOTAXIME<br>MIC = 1134-562 μM / 512-256 μg/mL (*E. coli* SA/2)<br>VANCOMYCIN<br>MIC = 707 μM / 1024 μg/mL (*E. faecalis* B3/101)	[3]
**64**	MIC reduction = 8-fold (*E. coli* SA/2)	CEFOTAXIME<br>MIC = 1134-562 μM / 512-256 μg/mL (*E. coli* SA/2)	[3]
**65**	MIC reduction = 2-fold (*E. coli* SA/2)	CEFOTAXIME<br>MIC = 1134-562 μM / 512-256 μg/mL (*E. coli* SA/2)	[3]
**66**	MIC reduction = 2-fold (*E. coli* SA/2)	CEFOTAXIME<br>MIC = 1134-562 μM / 512-256 μg/mL (*E. coli* SA/2)	[3]
**67**	MIC reduction = 4-fold (*E. faecalis* B3/101)	VANCOMYCIN<br>MIC = 707 μM / 1024 μg/mL (*E. faecalis* B3/101)	[3]
**68**	MIC reduction = 2-fold (*E. coli* SA/2)	CEFOTAXIME<br>MIC = 1134-562 μM / 512-256 μg/mL (*E. coli* SA/2)	[3]
**69**	MIC reduction = 2-fold (*E. faecalis* B3/101)	VANCOMYCIN<br>MIC = 707 μM / 1024 μg/mL (*E. faecalis* B3/101)	[3]
**70**	MIC reduction = 4-fold (*E. faecalis* B3/101)	VANCOMYCIN<br>MIC = 707 μM / 1024 μg/mL (*E. faecalis* B3/101)	[3]

**71**	MIC reduction = 2-fold (*E. faecalis* B3/101)	VANCOMYCIN<br>MIC = 707 μM / 1024 μg/mL (*E. faecalis* B3/101)	[3]
**Antiparasitic activity**			
**Compounds**		**Reference Drug**	**Reference**
**72**	IC_{50}= 24±3.0 μM (*L. braziliensis*)<br>IC_{50}= 53±2.5 μM (*L. chagasi*)<br>IC_{50}= 3±1.2 μM (*L. amazonensis*)	PENTAMIDINE ISETHIONATE<br>IC_{50}= 14±1.5 μM (*L. braziliensis*)<br>IC_{50}= 3±1.2 μM (*L. chagasi*)<br>IC_{50}= 0.46±1.2 μM (*L. amazonensis*)	[4]
**73**	IC_{50}= 36±2.5 μM (*L. braziliensis*)<br>IC_{50}= 6±0.5 μM (*L. chagasi*)<br>IC_{50}= 11±2.0 μM (*L. amazonensis*)	PENTAMIDINE ISETHIONATE<br>IC_{50}= 14±1.5 μM (*L. braziliensis*)<br>IC_{50}= 3±1.2 μM (*L. chagasi*)<br>IC_{50}= 0.46±1.2 μM (*L. amazonensis*)	[4]
**74**	IC_{50}= 23±2.0 μM (*L. braziliensis*)<br>IC_{50}= 50±1.5 μM (*L. chagasi*)<br>IC_{50}= 16±1.5 μM (*L. amazonensis*)	PENTAMIDINE ISETHIONATE<br>IC_{50}= 14±1.5 μM (*L. braziliensis*)<br>IC_{50}= 3±1.2 μM (*L. chagasi*)<br>IC_{50}= 0.46±1.2 μM (*L. amazonensis*)	[4]
**75**	IC_{50}= 40±1.2 μM (*L. braziliensis*)<br>IC_{50}= 88±2.3 μM (*L. chagasi*)<br>IC_{50}= 35±2.0 μM (*L. amazonensis*)	PENTAMIDINE ISETHIONATE<br>IC_{50}= 14±1.5 μM (*L. braziliensis*)<br>IC_{50}= 3±1.2 μM (*L. chagasi*)<br>IC_{50}= 0.46±1.2 μM (*L. amazonensis*)	[4]
**76**	IC_{50}= 23±0.6 μM (*L. braziliensis*)<br>IC_{50}= 58±1.2 μM (*L. chagasi*)<br>IC_{50}= 43±1.5 μM (*L. amazonensis*)	PENTAMIDINE ISETHIONATE<br>IC_{50}= 14±1.5 μM (*L. braziliensis*)<br>IC_{50}= 3±1.2 μM (*L. chagasi*)<br>IC_{50}= 0.46±1.2 μM (*L. amazonensis*)	[4]
**77**	IC_{50}= 60±1.5 μM (*L. braziliensis*)<br>IC_{50}= 128±3.5 μM (*L. chagasi*)<br>IC_{50}= 190±1.2 μM (*L. amazonensis*)	PENTAMIDINE ISETHIONATE<br>IC_{50}= 14±1.5 μM (*L. braziliensis*)<br>IC_{50}= 3±1.2 μM (*L. chagasi*)<br>IC_{50}= 0.46±1.2 μM (*L. amazonensis*)	[4]
**78**	IC_{50}= 78±2.0 μM (*L. braziliensis*)<br>IC_{50}= 310±2.5 μM (*L. chagasi*)<br>IC_{50}= 352±3.5 μM (*L. amazonensis*)	PENTAMIDINE ISETHIONATE<br>IC_{50}= 14±1.5 μM (*L. braziliensis*)<br>IC_{50}= 3±1.2 μM (*L. chagasi*)<br>IC_{50}= 0.46±1.2 μM (*L. amazonensis*)	[4]
**79**	IC_{50} = 6.1±1.0 μM (Epimastigote of *T. Cruzi*)<br>EC_{50} = 69.2±0.9 μM (Trypomastigote of *T. Cruzi*)<br>IC_{50} = 3.7±0.4 μM (Promastigote of *L. amazonesis*)	BENZNIDAZOLE<br>IC_{50} = 6.5±0.7 μM (Epimastigote of *T. Cruzi*)<br>EC_{50} = 34.5±7.6 μM (Trypomastigote of *T. Cruzi*)<br>AMPHOTERICIN B<br>IC_{50} = 0.06±0.00 μM (Promastigote of *L. amazonesis*)	[5]
**80**	IC_{50} = 9.7±0.8 μM (Epimastigote of *T. Cruzi*)<br>EC_{50} = 66.7±1.7 μM (Trypomastigote of *T. Cruzi*)<br>IC_{50} = 9.8±2.4 μM (Promastigote of *L. amazonesis*)	BENZNIDAZOLE<br>IC_{50} = 6.5±0.7 μM (Epimastigote of *T. Cruzi*)<br>EC_{50} = 34.5±7.6 μM (Trypomastigote of *T. Cruzi*)<br>AMPHOTERICIN B<br>IC_{50} = 0.06±0.00 μM (Promastigote of *L. amazonesis*)	[5]
**81**	IC_{50} = 17.8±1.0 μM (Epimastigote of *T. Cruzi*)	BENZNIDAZOLE<br>IC_{50} = 6.5±0.7 μM (Epimastigote of *T. Cruzi*)	[5]

	EC_{50} = 21.8±0.1 μM (Trypomastigote of *T. Cruzi*)<br>IC_{50} = 19.5±2.1 μM (Promastigote of *L. amazonesis*)	EC_{50} = 34.5±7.6 μM (Trypomastigote of *T. Cruzi*)<br>AMPHOTERICIN B<br>IC_{50} = 0.06±0.00 μM (Promastigote of *L. amazonesis*)	
**82**	IC_{50} = 18.9±0.0 μM (Epimastigote of *T. Cruzi*)<br>EC_{50} = 23.0±0.6 μM (Trypomastigote of *T. Cruzi*)<br>IC_{50} = 13.8±1.1 μM (Promastigote of *L. amazonesis*)	BENZNIDAZOLE<br>IC_{50} = 6.5±0.7 μM (Epimastigote of *T. Cruzi*)<br>EC_{50} = 34.5±7.6 μM (Trypomastigote of *T. Cruzi*)<br>AMPHOTERICIN B<br>IC_{50} = 0.06±0.00 μM (Promastigote of *L. amazonesis*)	[5]
**83**	IC_{50} = 20.0±2.6 μM (Epimastigote of *T. Cruzi*)<br>EC_{50} = 21.1±1.8 μM (Trypomastigote of *T. Cruzi*)<br>IC_{50} = 17.3±1.1 μM (Promastigote of *L. amazonesis*)	BENZNIDAZOLE<br>IC_{50} = 6.5±0.7 μM (Epimastigote of *T. Cruzi*)<br>EC_{50} = 34.5±7.6 μM (Trypomastigote of *T. Cruzi*)<br>AMPHOTERICIN B<br>IC_{50} = 0.06±0.00 μM (Promastigote of *L. amazonesis*)	[5]
**84**	IC_{50} = 21.7±3.4 μM (Epimastigote of *T. Cruzi*)<br>EC_{50} = 25.0±2.6 μM (Trypomastigote of *T. Cruzi*)<br>IC_{50} = 21.7±3.6 μM (Promastigote of *L. amazonesis*)	BENZNIDAZOLE<br>IC_{50} = 6.5±0.7 μM (Epimastigote of *T. Cruzi*)<br>EC_{50} = 34.5±7.6 μM (Trypomastigote of *T. Cruzi*)<br>AMPHOTERICIN B<br>IC_{50} = 0.06±0.00 μM (Promastigote of *L. amazonesis*)	[5]
**85**	IC_{50} = 19.6±0.5 μM (Epimastigote of *T. Cruzi*)<br>EC_{50}= 27.9±3.8 μM (Trypomastigote of *T. Cruzi*)<br>IC_{50}= 8.2±1.1 μM (Promastigote of *L. amazonesis*)	BENZNIDAZOLE<br>IC_{50} = 6.5±0.7 μM (Epimastigote of *T. Cruzi*)<br>EC_{50} = 34.5±7.6 μM (Trypomastigote of *T. Cruzi*)<br>AMPHOTERICIN B<br>IC_{50} = 0.06±0.00 μM (Promastigote of *L. amazonesis*)	[5]
**86**	IC_{50} = 15.1±0.4 μM (Epimastigote of *T. Cruzi*)<br>EC_{50} = 19.7±2.8 μM (Trypomastigote of *T. Cruzi*)<br>IC_{50} = 19.3±1.1 μM (Promastigote of *L. amazonesis*)	BENZNIDAZOLE<br>IC_{50} = 6.5±0.7 μM (Epimastigote of *T. Cruzi*)<br>EC_{50} = 34.5±7.6 μM (Trypomastigote of *T. Cruzi*)<br>AMPHOTERICIN B<br>IC_{50} = 0.06±0.00 μM (Promastigote of *L. amazonesis*)	[5]
**87**	IC_{50} = 15.2±1.8 μM (Epimastigote of *T. Cruzi*)<br>EC_{50} = 67.6±0.5 μM (Trypomastigote of *T. Cruzi*)<br>IC_{50} = 32.2±3.6 μM (Promastigote of *L. amazonesis*)	BENZNIDAZOLE<br>IC_{50} = 6.5±0.7 μM (Epimastigote of *T. Cruzi*)<br>EC_{50} = 34.5±7.6 μM (Trypomastigote of *T. Cruzi*)<br>AMPHOTERICIN B	[5]

		IC_{50} = 0.06±0.00 μM (Promastigote of *L. amazonesis*)	
**88**	IC_{50} = 16.8±0.4 μM (Epimastigote of *T. Cruzi*)<br>EC_{50} = 14.8±0.0 μM (Trypomastigote of *T. Cruzi*)<br>IC_{50} = 13.8±0.4 μM (Promastigote of *L. amazonesis*)	BENZNIDAZOLE<br>IC_{50} = 6.5±0.7 μM (Epimastigote of *T. Cruzi*)<br>EC_{50} = 34.5±7.6 μM (Trypomastigote of *T. Cruzi*)<br>AMPHOTERICIN B<br>IC_{50} = 0.06±0.00 μM (Promastigote of *L. amazonesis*)	[5]
**89**	IC_{50} = 17.2±0.9 μM (Epimastigote of *T. Cruzi*)<br>EC_{50} = 20.3±2.2 μM (Trypomastigote of *T. Cruzi*)<br>IC_{50} = 17.2±2.6 μM (Promastigote of *L. amazonesis*)	BENZNIDAZOLE<br>IC_{50} = 6.5±0.7 μM (Epimastigote of *T. Cruzi*)<br>EC_{50} = 34.5±7.6 μM (Trypomastigote of *T. Cruzi*)<br>AMPHOTERICIN B<br>IC_{50} = 0.06±0.00 μM (Promastigote of *L. amazonesis*)	[5]
**90**	IC_{50} > 100 μM (Epimastigote of *T. Cruzi*)<br>EC_{50}> 100 μM (Trypomastigote of *T. Cruzi*)<br>IC_{50} = 76.3±4.0 μM (Promastigote of *L. amazonesis*)	BENZNIDAZOLE<br>IC_{50} = 6.5±0.7 μM (Epimastigote of *T. Cruzi*)<br>EC_{50} = 34.5±7.6 μM (Trypomastigote of *T. Cruzi*)<br>AMPHOTERICIN B<br>IC_{50} = 0.06±0.00 μM (Promastigote of *L. amazonesis*)	[5]
**91**	IC_{50} = 10.2±2.7 μM (Epimastigote of *T. Cruzi*)<br>EC_{50}= 25.6±4.4 μM (Trypomastigote of *T. Cruzi*)<br>IC_{50}= 2.4±0.2 μM (Promastigote of *L. amazonesis*)	BENZNIDAZOLE<br>IC_{50} = 6.5±0.7 μM (Epimastigote of *T. Cruzi*)<br>EC_{50} = 34.5±7.6 μM (Trypomastigote of *T. Cruzi*)<br>AMPHOTERICIN B<br>IC_{50} = 0.06±0.00 μM (Promastigote of *L. amazonesis*)	[5]
**92**	IC_{50} = 5.5±0.9 μM (Epimastigote of *T. Cruzi*)<br>EC_{50}= 19.1±3.4 μM (Trypomastigote of *T. Cruzi*)<br>IC_{50}= 2.0±0.2 μM (Promastigote of *L. amazonesis*)	BENZNIDAZOLE<br>IC_{50} = 6.5±0.7 μM (Epimastigote of *T. Cruzi*)<br>EC_{50} = 34.5±7.6 μM (Trypomastigote of *T. Cruzi*)<br>AMPHOTERICIN B<br>IC_{50} = 0.06±0.00 μM (Promastigote of *L. amazonesis*)	[5]
**93**	IC_{50} = 65.2±4.4 μM (Epimastigote of *T. Cruzi*)<br>EC_{50} = 74.2±4.2 μM (Trypomastigote of *T. Cruzi*)<br>IC_{50} = 17.4±1.1 μM (Promastigote of *L. amazonesis*)	BENZNIDAZOLE<br>IC_{50} = 6.5±0.7 μM (Epimastigote of *T. Cruzi*)<br>EC_{50} = 34.5±7.6 μM (Trypomastigote of *T. Cruzi*)<br>AMPHOTERICIN B<br>IC_{50} = 0.06±0.00 μM (Promastigote of *L. amazonesis*)	[5]
**94**	IC_{50} = 2.8±0.9 μM (Epimastigote of *T. Cruzi*)	BENZNIDAZOLE	[5]

	EC_{50} = 7.6±0.4 μM (Trypomastigote of *T. Cruzi*)<br>IC_{50}= 2.9±0.0 μM (Promastigote of *L. amazonesis*)	IC_{50} = 6.5±0.7 μM (Epimastigote of *T. Cruzi*)<br>EC_{50} = 34.5±7.6 μM (Trypomastigote of *T. Cruzi*)<br>AMPHOTERICIN B<br>IC_{50} = 0.06±0.00 μM (Promastigote of *L. amazonesis*)	
**95**	IC_{50} = 1.8±0.4 μM (Epimastigote of *T. Cruzi*)<br>EC_{50}= 20.0±2.1 μM (Trypomastigote of *T. Cruzi*)<br>IC_{50}= 1.3±0.2 μM (Promastigote of *L. amazonesis*)	BENZNIDAZOLE<br>IC_{50} = 6.5±0.7 μM (Epimastigote of *T. Cruzi*)<br>EC_{50} = 34.5±7.6 μM (Trypomastigote of *T. Cruzi*)<br>AMPHOTERICIN B<br>IC_{50} = 0.06±0.00 μM (Promastigote of *L. amazonesis*)	[5]
**96**	IC_{50} = 5.4±0.1 μM (Epimastigote of *T. Cruzi*)<br>EC_{50}= 19.5±1.8 μM (Trypomastigote of *T. Cruzi*)<br>IC_{50}= 1.4±0.2 μM (Promastigote of *L. amazonesis*)	BENZNIDAZOLE<br>IC_{50} = 6.5±0.7 μM (Epimastigote of *T. Cruzi*)<br>EC_{50} = 34.5±7.6 μM (Trypomastigote of *T. Cruzi*)<br>AMPHOTERICIN B<br>IC_{50} = 0.06±0.00 μM (Promastigote of *L. amazonesis*)	[5]
**97**	IC_{50} = 2.5±0.1 μM (Epimastigote of *T.Cruzi*)<br>EC_{50}= 17.9±2.1 μM (Trypomastigote of *T. Cruzi*)<br>IC_{50}= 0.5±0.0 μM (Promastigote of *L. amazonesis*)	BENZNIDAZOLE<br>IC_{50} = 6.5±0.7 μM (Epimastigote of *T.Cruzi*)<br>EC_{50} = 34.5±7.6 μM (Trypomastigote of *T. Cruzi*)<br>AMPHOTERICIN B<br>IC_{50} = 0.06±0.00 μM (Promastigote of *L. amazonesis*)	[5]
**ANTIOXIDANT ACTIVITY**			
**Compound**		**Reference drug**	**Reference**
**8**	K= 1.41 ± 0.013 $min^{-1}x10^{-2}$<br>Inhibition = 23.81 ± 5.82%<br>Viability = 81%	CURCUMIN<br>K= 3.30 ± 0.11 $min^{-1}x10^{-2}$<br>Inhibition = 99.55 ± 0.01%<br>Viability = 92%	[6]
**98**	K= 2.15 ± 0.116 $min^{-1}x10^{-2}$<br>Inhibition = 70.76 ± 3.09%<br>Viability = 64%	CURCUMIN<br>K= 3.30 ± 0.11 $min^{-1}x10^{-2}$<br>Inhibition = 99.55 ± 0.01%<br>Viability = 92%	[6]
**99**	K= 7.30 ± 0.11 $min^{-1}x10^{-2}$<br>Inhibition = 79.01 ± 3.88%<br>Viability = 70%	CURCUMIN<br>K= 3.30 ± 0.11 $min^{-1}x10^{-2}$<br>Inhibition = 99.55 ± 0.01%<br>Viability = 92%	[6]
**100**	K= 10.25 ± 0.103$min^{-1}x10^{-2}$<br>Inhibition = 90.71 ± 1.22%<br>Viability = 81%	CURCUMIN<br>K= 3.30 ± 0.11 $min^{-1}x10^{-2}$<br>Inhibition = 99.55 ± 0.01%<br>Viability = 92%	[6]
**101**	K= 3.06 ± 0.057$min^{-1}x10^{-2}$<br>Inhibition = 73.01 ± 3.87%<br>Viability = 86%	CURCUMIN<br>K= 3.30 ± 0.11 $min^{-1}x10^{-2}$<br>Inhibition = 99.55 ± 0.01%<br>Viability = 92%	[6]

**102**	K= 12.15 ± 0.120min^{-1}x10^{-2}<br>Inhibition = 91.24 ± 2.35%<br>Viability = 68%	CURCUMIN<br>K= 3.30 ± 0.11 min^{-1}x10^{-2}<br>Inhibition = 99.55 ± 0.01%<br>Viability = 92%	[6]
**103**	K= 16.32 ± 0.035 mim^{-1}x10^{-2}<br>Inhibition = 96.91 ± 0.88%<br>Viability = 82%	CURCUMIN<br>K= 3.30 ± 0.11 min^{-1}x10^{-2}<br>Inhibition = 99.55 ± 0.01%<br>Viability = 92%	[6]
**104**	K= 12.41 ± 0.032 min^{-1}x10^{-2}<br>Inhibition = 94.26 ± 1.25%<br>Viability = 88%	CURCUMIN<br>K= 3.30 ± 0.11 min^{-1}x10^{-2}<br>Inhibition = 99.55 ± 0.01%<br>Viability = 92%	[6]
**105**	K= 6.91 ± 0.325 min^{-1}x10^{-2}<br>Inhibition = 78.27 ± 2.32%<br>Viability = 75%	CURCUMIN<br>K= 3.30 ± 0.11 min^{-1}x10^{-2}<br>Inhibition = 99.55 ± 0.01%<br>Viability = 92%	[6]
**106**	K= 14.80 ± 113 min^{-1}x10^{-2}	CURCUMIN<br>K= 3.30 ± 0.11 min^{-1}x10^{-2}<br>Inhibition = 99.55 ± 0.01%<br>Viability = 92%	[6]
**107**	K= 5.91 ± 0.130 min^{-1}x10^{-2}<br>Inhibition = 63.42 ± 3.52%<br>Viability = 85%	CURCUMIN<br>K= 3.30 ± 0.11 min^{-1}x10^{-2}<br>Inhibition = 99.55 ± 0.01%<br>Viability = 92%	[6]
**108**	K= 11.92 ± 111 min^{-1}x10^{-2}<br>Inhibition = 77.06 ± 12.88%<br>Viability = 79%	CURCUMIN<br>K= 3.30 ± 0.11 min^{-1}x10^{-2}<br>Inhibition = 99.55 ± 0.01%<br>Viability = 92%	[6]
**109**	K= 3.41 ± 0.113 min^{-1}x10^{-2}	CURCUMIN<br>K= 3.30 ± 0.11 min^{-1}x10^{-2}<br>Inhibition = 99.55 ± 0.01%<br>Viability = 92%	[6]
**110**	K= 1.82 ± 0.124 min^{-1}x10^{-2}<br>Inhibition = 41.49 ± 13.04%<br>Viability = 77%	CURCUMIN<br>K= 3.30 ± 0.11 min^{-1}x10^{-2}<br>Inhibition = 99.55 ± 0.01%<br>Viability = 92%	[6]
**8**	IC_{50} > 300 μM	CURCUMIN<br>IC_{50} = 21.62 ± 1.21 μM	[7]
**22**	IC_{50} > 300 μM	CURCUMIN<br>IC_{50} = 21.62 ± 1.21 μM	[7]
**23**	IC_{50} = 48.90 ± 1.69μM	CURCUMIN<br>IC_{50} = 21.62 ± 1.21 μM	[7]
**33**	IC_{50} > 300 μM	CURCUMIN<br>IC_{50} = 21.62 ± 1.21 μM	[7]
**34**	IC_{50} = 48.40±2.06μM	CURCUMIN<br>IC_{50} = 21.62 ± 1.21 μM	[7]
**109**	IC_{50} = 40.20 ± 2.03 μM	CURCUMIN<br>IC_{50} = 21.62 ± 1.21 μM	[7]
**110**	IC_{50} = 38.40 ± 1.65μM	CURCUMIN<br>IC_{50} = 21.62 ± 1.21 μM	[7]
**111**	IC_{50} = 5.79 ± 0.12 μM	CURCUMIN<br>IC_{50} = 21.62 ± 1.21 μM	[7]

**112**	IC_{50} = 59.20 ± 3.01 μM	CURCUMIN<br>IC_{50} = 21.62 ± 1.21 μM	[7]
**113**	No inhibition	CURCUMIN<br>IC_{50} = 21.62 ± 1.21 μM	[7]
**114**	IC_{50} = 17.90 ± 0.54 μM	CURCUMIN<br>IC_{50} = 21.62 ± 1.21 μM	[7]
**115**	IC_{50} = 78.06 ± 3.28 μM	CURCUMIN<br>IC_{50} = 21.62 ± 1.21 μM	[7]
**116**	IC_{50} > 300 μM	CURCUMIN<br>IC_{50} = 21.62 ± 1.21 μM	[7]
**117**	IC_{50} > 300 μM	CURCUMIN<br>IC_{50} = 21.62 ± 1.21 μM	[7]
**118**	IC_{50} = 124.62 ± 6.78 μM	CURCUMIN<br>IC_{50} = 21.62 ± 1.21 μM	[7]
**119**	IC_{50} = 47.10 ± 4.33 μM	CURCUMIN<br>IC_{50} = 21.62 ± 1.21 μM	[7]
**120**	IC_{50} = 6.73 ± 0.18 μM	CURCUMIN<br>IC_{50} = 21.62 ± 1.21 μM	[7]
**121**	IC_{50} = 65.72 ± 2.87 μM	CURCUMIN<br>IC_{50} = 21.62 ± 1.21 μM	[7]
**122**	No inhibition	CURCUMIN<br>IC_{50} = 21.62 ± 1.21 μM	[7]
**123**	IC_{50} = 96.4 ± 5.40 μM	CURCUMIN<br>IC_{50} = 21.62 ± 1.21 μM	[7]
**124**	IC_{50} > 300 μM	CURCUMIN<br>IC_{50} = 21.62 ± 1.21 μM	[7]
**125**	IC_{50} > 300 μM	CURCUMIN<br>IC_{50} = 21.62 ± 1.21 μM	[7]
**126**	IC_{50} = 186.42 ± 8.21 μM	CURCUMIN<br>IC_{50} = 21.62 ± 1.21 μM	[7]
**127**	IC_{50} = 48.24 ± 3.78 μM	CURCUMIN<br>IC_{50} = 21.62 ± 1.21 μM	[7]
**128**	IC_{50} = 16.82 ± 0.87μM	CURCUMIN<br>IC_{50} = 21.62 ± 1.21 μM	[7]
**129**	IC_{50} = 70.10 ± 3.65μM	CURCUMIN<br>IC_{50} = 21.62 ± 1.21 μM	[7]
**130**	No inhibition	CURCUMIN<br>IC_{50} = 21.62 ± 1.21 μM	[7]
**131**	IC_{50} =53.68 ± 1.68 μM	CURCUMIN<br>IC_{50} = 21.62 ± 1.21 μM	[7]
**132**	IC_{50} = 73.80 ± 3.98 μM	CURCUMIN<br>IC_{50} = 21.62 ± 1.21 μM	[7]
**133**	IC_{50} > 300 μM	CURCUMIN<br>IC_{50} = 21.62 ± 1.21 μM	[7]
**134**	IC_{50} > 300 μM	CURCUMIN<br>IC_{50} = 21.62 ± 1.21 μM	[7]
**135**	IC_{50} = 216.81 ± 11.36 μM	CURCUMIN<br>IC_{50} = 21.62 ± 1.21 μM	[7]
**136**	IC_{50} = 50.60 ± 3.23μM	CURCUMIN<br>IC_{50} = 21.62 ± 1.21 μM	[7]
**137**	IC_{50} > 300 μM	CURCUMIN<br>IC_{50} = 21.62 ± 1.21 μM	[7]

**138**	IC_{50} = 29.26 ± 1.42 μM	CURCUMIN<br>IC_{50} = 21.62 ± 1.21 μM	[7]
**139**	IC_{50} = 65.18 ± 3.72 μM	CURCUMIN<br>IC_{50} = 21.62 ± 1.21 μM	[7]
**140**	IC_{50} = 71.56 ± 3.83 μM	CURCUMIN<br>IC_{50} = 21.62 ± 1.21 μM	[7]
**141**	No inhibition	CURCUMIN<br>IC_{50} = 21.62 ± 1.21 μM	[7]
**142**	IC_{50} = 85.10 ± 4.32 μM	CURCUMIN<br>IC_{50} = 21.62 ± 1.21 μM	[7]
**143**	IC_{50} = 103.20 ± 5.12 μM	CURCUMIN<br>IC_{50} = 21.62 ± 1.21 μM	[7]
**144**	IC_{50} > 300 μM	CURCUMIN<br>IC_{50} = 21.62 ± 1.21 μM	[7]
**145**	IC_{50} = 18.80 ± 0.51 μM	CURCUMIN<br>IC_{50} = 21.62 ± 1.21 μM	[7]
**146**	IC_{50} = 56.90 ± 3.18 μM	CURCUMIN<br>IC_{50} = 21.62 ± 1.21 μM	[7]
**147**	IC_{50} = 53.70 ± 2.32 μM	CURCUMIN<br>IC_{50} = 21.62 ± 1.21 μM	[7]
**66**	No inhibition	CURCUMIN<br>IC_{50} = 21.62 ± 1.21 μM	[7]
**148**	IC_{50} = 49.06 ± 1.87 μM	CURCUMIN<br>IC_{50} = 21.62 ± 1.21 μM	[7]
**149**	IC_{50} = 82.78 ± 4.67 μM	CURCUMIN<br>IC_{50} = 21.62 ± 1.21 μM	[7]
**150**	IC_{50} > 300 μM	CURCUMIN<br>IC_{50} = 21.62 ± 1.21 μM	[7]
**151**	IC_{50} > 300 μM	CURCUMIN<br>IC_{50} = 21.62 ± 1.21 μM	[7]
**152**	IC_{50} > 300 μM	CURCUMIN<br>IC_{50} = 21.62 ± 1.21 μM	[7]
**153**	IC_{50} > 300 μM	CURCUMIN<br>IC_{50} = 21.62 ± 1.21 μM	[7]
**154**	IC_{50} = 21.10 ± 0.86 μM	CURCUMIN<br>IC_{50} = 21.62 ± 1.21 μM	[7]
**155**	IC_{50} = 53.90 ± 2.37 μM	CURCUMIN<br>IC_{50} = 21.62 ± 1.21 μM	[7]
**156**	IC_{50} = 52.20 ± 2.13 μM	CURCUMIN<br>IC_{50} = 21.62 ± 1.21 μM	[7]
**157**	No inhibition	CURCUMIN<br>IC_{50} = 21.62 ± 1.21 μM	[7]
**158**	IC_{50} = 58.70 ± 2.86 μM	CURCUMIN<br>IC_{50} = 21.62 ± 1.21 μM	[7]
**159**	IC_{50} = 83.64 ± 3.54 μM	CURCUMIN<br>IC_{50} = 21.62 ± 1.21 μM	[7]
**160**	IC_{50} > 300 μM	CURCUMIN<br>IC_{50} = 21.62 ± 1.21 μM	[7]
**161**	IC_{50} > 300 μM	CURCUMIN<br>IC_{50} = 21.62 ± 1.21 μM	[7]
**162**	IC_{50} > 300 μM	CURCUMIN<br>IC_{50} = 21.62 ± 1.21 μM	[7]

**22**	IC_{50} > 200 μg/ml	ASCORBIC ACID<br>IC_{50} = 51.5 μg/ml	[8]
**163**	IC_{50} = 49.1 μg/ml	ASCORBIC ACID<br>IC_{50} = 51.5 μg/ml	[8]
**164**	IC_{50} = 64.6 μg/ml	ASCORBIC ACID<br>IC_{50} = 51.5 μg/ml	[8]
**33**	Inhibition = 9.49 %	CURCUMIN<br>Inhibition = 87.64 %<br>ASCORBIC ACID<br>Inhibition = 80.81%	[9]
**34**	Inhibition = 74.22%	CURCUMIN<br>Inhibition = 87.64 %<br>ASCORBIC ACID<br>Inhibition = 80.81%	[9]
**165**	Inhibition = 1.93 %	CURCUMIN<br>Inhibition = 87.64 %<br>ASCORBIC ACID<br>Inhibition = 80.81%	[9]
**166**	Inhibition = 0.58 %	CURCUMIN<br>Inhibition = 87.64 %<br>ASCORBIC ACID<br>Inhibition = 80.81%	[9]
**167**	Inhibition = 1.35 %	CURCUMIN<br>Inhibition = 87.64 %<br>ASCORBIC ACID<br>Inhibition = 80.81%	[9]
**168**	Inhibition = 0 %	CURCUMIN<br>Inhibition = 87.64 %<br>ASCORBIC ACID<br>Inhibition = 80.81%	[9]
**169**	Inhibition = 1.16 %	CURCUMIN<br>Inhibition = 87.64 %<br>ASCORBIC ACID<br>Inhibition = 80.81%	[9]
**170**	Inhibition = 1.74%	CURCUMIN<br>Inhibition = 87.64 %<br>ASCORBIC ACID<br>Inhibition = 80.81%	[9]
**171**	Inhibition = 0.77%	CURCUMIN<br>Inhibition = 87.64 %<br>ASCORBIC ACID<br>Inhibition = 80.81%	[9]
**172**	Inhibition = 5.03%	CURCUMIN<br>Inhibition = 87.64 %<br>ASCORBIC ACID<br>Inhibition = 80.81%	[9]
**8**	IC_{50} > 300 μM	CURCUMIN<br>IC_{50} = 35.6 ± 0.8 μM<br>VITAMIN C<br>IC_{50} = 21.1 ± 1.5 μM	[10]
**9**	IC_{50} = 17.1 ± 1.5 μM	CURCUMIN<br>IC_{50} = 35.6 ± 0.8 μM	[10]

		VITAMIN C<br>IC_{50} = 21.1 ± 1.5 μM	
**22**	IC_{50} = 276 ± 2.6 μM	CURCUMIN<br>IC_{50} = 35.6 ± 0.8 μM<br>VITAMIN C<br>IC_{50} = 21.1 ± 1.5 μM	[10]
**23**	IC_{50} = 9.5 ± 1.7 μM	CURCUMIN<br>IC_{50} = 35.6 ± 0.8 μM<br>VITAMIN C<br>IC_{50} = 21.1 ± 1.5 μM	[10]
**33**	IC_{50} > 300 μM	CURCUMIN<br>IC_{50} = 35.6 ± 0.8 μM<br>VITAMIN C<br>IC_{50} = 21.1 ± 1.5 μM	[10]
**34**	IC_{50} = 17.2 ± 2.4 μM	CURCUMIN<br>IC_{50} = 35.6 ± 0.8 μM<br>VITAMIN C<br>IC_{50} = 21.1 ± 1.5 μM	[10]
**110**	IC_{50} = 32.6 ± 4.1 μM	CURCUMIN<br>IC_{50} = 35.6 ± 0.8 μM<br>VITAMIN C<br>IC_{50} = 21.1 ± 1.5 μM	[10]
**111**	IC_{50} = 1.9 ± 0.6 μM	CURCUMIN<br>IC_{50} = 35.6 ± 0.8 μM<br>VITAMIN C<br>IC_{50} = 21.1 ± 1.5 μM	[10]
**112**	IC_{50} > 800 μM	CURCUMIN<br>IC_{50} = 35.6 ± 0.8 μM<br>VITAMIN C<br>IC_{50} = 21.1 ± 1.5 μM	[10]
**1114**	IC_{50} = 6.6 ± 1.4 μM	CURCUMIN<br>IC_{50} = 35.6 ± 0.8 μM<br>VITAMIN C<br>IC_{50} = 21.1 ± 1.5 μM	[10]
**115**	IC_{50} = 81.1 ± 1.2 μM	CURCUMIN<br>IC_{50} = 35.6 ± 0.8 μM<br>VITAMIN C<br>IC_{50} = 21.1 ± 1.5 μM	[10]
**120**	IC_{50} = 2.9 ± 0.7 μM	CURCUMIN<br>IC_{50} = 35.6 ± 0.8 μM<br>VITAMIN C<br>IC_{50} = 21.1 ± 1.5 μM	[10]
**122**	IC_{50} > 800 μM	CURCUMIN<br>IC_{50} = 35.6 ± 0.8 μM<br>VITAMIN C<br>IC_{50} = 21.1 ± 1.5 μM	[10]
**123**	IC_{50} = 101.5 ± 0.5 μM	CURCUMIN<br>IC_{50} = 35.6 ± 0.8 μM<br>VITAMIN C<br>IC_{50} = 21.1 ± 1.5 μM	[10]
**128**	IC_{50} = 1.5 ± 0.4 μM	CURCUMIN<br>IC_{50} = 35.6 ± 0.8 μM	[10]

		VITAMIN C<br>IC_{50} = 21.1 ± 1.5 μM	
**130**	IC_{50} > 800 μM	CURCUMIN<br>IC_{50} = 35.6 ± 0.8 μM<br>VITAMIN C<br>IC_{50} = 21.1 ± 1.5 μM	[10]
**131**	IC_{50} = 28 ± 2.8 μM	CURCUMIN<br>IC_{50} = 35.6 ± 0.8 μM<br>VITAMIN C<br>IC_{50} = 21.1 ± 1.5 μM	[10]
**132**	IC_{50} = 8.4 ± 1.3 μM	CURCUMIN<br>IC_{50} = 35.6 ± 0.8 μM<br>VITAMIN C<br>IC_{50} = 21.1 ± 1.5 μM	[10]
**137**	IC_{50} = 290 ± 3.2 μM	CURCUMIN<br>IC_{50} = 35.6 ± 0.8 μM<br>VITAMIN C<br>IC_{50} = 21.1 ± 1.5 μM	[10]
**138**	IC_{50} = 1.7 ± 0.5 μM	CURCUMIN<br>IC_{50} = 35.6 ± 0.8 μM<br>VITAMIN C<br>IC_{50} = 21.1 ± 1.5 μM	[10]
**139**	IC_{50} = 9.8 ± 2.2 μM	CURCUMIN<br>IC_{50} = 35.6 ± 0.8 μM<br>VITAMIN C<br>IC_{50} = 21.1 ± 1.5 μM	[10]
**140**	IC_{50} = 27.2 ± 5.2 μM	CURCUMIN<br>IC_{50} = 35.6 ± 0.8 μM<br>VITAMIN C<br>IC_{50} = 21.1 ± 1.5 μM	[10]
**141**	IC_{50} = 14.6 ± 2.0 μM	CURCUMIN<br>IC_{50} = 35.6 ± 0.8 μM<br>VITAMIN C<br>IC_{50} = 21.1 ± 1.5 μM	[10]
**142**	IC_{50} = 62.8 ± 1.9 μM	CURCUMIN<br>IC_{50} = 35.6 ± 0.8 μM<br>VITAMIN C<br>IC_{50} = 21.1 ± 1.5 μM	[10]
**173**	IC_{50} = 22.3 ± 3.3 μM	CURCUMIN<br>IC_{50} = 35.6 ± 0.8 μM<br>VITAMIN C<br>IC_{50} = 21.1 ± 1.5 μM	[10]
**174**	IC_{50} = 14.6 ± 2.4 μM	CURCUMIN<br>IC_{50} = 35.6 ± 0.8 μM<br>VITAMIN C<br>IC_{50} = 21.1 ± 1.5 μM	[10]
**175**	IC_{50} = 33.6 ± 4.1 μM	CURCUMIN<br>IC_{50} = 35.6 ± 0.8 μM<br>VITAMIN C<br>IC_{50} = 21.1 ± 1.5 μM	[10]
**4**	IC_{50} > 4819 μM (DPPH)<br>IC_{50} > 4819μM (ABTS)	CURCUMIN<br>IC_{50} =13.53 ± 0.50 μM (DPPH)	[11]

	IC_{50} > 4819 (TRAP)<br>IC_{50} > 4819 (NET)	IC_{50} = 48.63 ± 2.13 μM (ABTS)<br>IC_{50} = 54.35 ± 1.95 (TRAP)<br>IC_{50} = 89.82 ± 1.76 (NET)<br>VITAMIN C<br>IC_{50} =45.41 ± 1.80 μM (DPPH)<br>IC_{50} = 105.47 ± 3.22 μM (ABTS)<br>IC_{50} = 102.22 ± 2.44 (TRAP)<br>IC_{50} = 167.31 ± 1.54 (NET)	
**9**	IC_{50} = 9.24 ± 1.84μM (DPPH)<br>IC_{50} = 30.25 ± 1.98μM (ABTS)<br>IC_{50} = 44.54 ± 1.42 (TRAP)<br>IC_{50} = 72.62 ± 1.44 (NET)	CURCUMIN<br>IC_{50} =13.53 ± 0.50 μM (DPPH)<br>IC_{50} = 48.63 ± 2.13 μM (ABTS)<br>IC_{50} = 54.35 ± 1.95 (TRAP)<br>IC_{50} = 89.82 ± 1.76 (NET)<br>VITAMIN C<br>IC_{50} =45.41 ± 1.80 μM (DPPH)<br>IC_{50} = 105.47 ± 3.22 μM (ABTS)<br>IC_{50} = 102.22 ± 2.44 (TRAP)<br>IC_{50} = 167.31 ± 1.54 (NET)	[11]
**122**	IC_{50} > 5633μM (DPPH)<br>IC_{50} > 5633 μM (ABTS)<br>IC_{50} > 5633 (TRAP)<br>IC_{50} > 5633 (NET)	CURCUMIN<br>IC_{50} =13.53 ± 0.50 μM (DPPH)<br>IC_{50} = 48.63 ± 2.13 μM (ABTS)<br>IC_{50} = 54.35 ± 1.95 (TRAP)<br>IC_{50} = 89.82 ± 1.76 (NET)<br>VITAMIN C<br>IC_{50} =45.41 ± 1.80 μM (DPPH)<br>IC_{50} = 105.47 ± 3.22 μM (ABTS)<br>IC_{50} = 102.22 ± 2.44 (TRAP)<br>IC_{50} = 167.31 ± 1.54 (NET)	[11]
**176**	IC_{50} = 92.02 ± 0.91 μM (DPPH)<br>IC_{50} = 176.67 ± 2.15 μM (ABTS)<br>IC_{50} = 193.43 ± 2.95 μM (TRAP)<br>IC_{50} = 204.00 ± 0.47 μM (NET)	CURCUMIN<br>IC_{50} =13.53 ± 0.50 μM (DPPH)<br>IC_{50} = 48.63 ± 2.13 μM (ABTS)<br>IC_{50} = 54.35 ± 1.95 (TRAP)<br>IC_{50} = 89.82 ± 1.76 (NET)<br>VITAMIN C<br>IC_{50} =45.41 ± 1.80 μM (DPPH)<br>IC_{50} = 105.47 ± 3.22 μM (ABTS)<br>IC_{50} = 102.22 ± 2.44 (TRAP)<br>IC_{50} = 167.31 ± 1.54 (NET)	[11]
**177**	IC_{50} = 50.09 ± 3.42 μM (DPPH)<br>IC_{50} = 128.35 ± 2.66 μM (ABTS)<br>IC_{50} = 128.05 ± 3.48 (TRAP)<br>IC_{50} = 193.33 ± 3.23 (NET)	CURCUMIN<br>IC_{50} =13.53 ± 0.50 μM (DPPH)<br>IC_{50} = 48.63 ± 2.13 μM (ABTS)<br>IC_{50} = 54.35 ± 1.95 (TRAP)<br>IC_{50} = 89.82 ± 1.76 (NET)<br>VITAMIN C<br>IC_{50} =45.41 ± 1.80 μM (DPPH)<br>IC_{50} = 105.47 ± 3.22 μM (ABTS)<br>IC_{50} = 102.22 ± 2.44 (TRAP)<br>IC_{50} = 167.31 ± 1.54 (NET)	[11]
**178**	IC_{50} = 41.46 ± 1.92μM (DPPH)<br>IC_{50} = 103.03 ± 1.67μM (ABTS)<br>IC_{50} = 136.24 ± 3.13 (TRAP)<br>IC_{50} = 178.22 ± 1.04 (NET)	CURCUMIN<br>IC_{50} =13.53 ± 0.50 μM (DPPH)<br>IC_{50} = 48.63 ± 2.13 μM (ABTS)<br>IC_{50} = 54.35 ± 1.95 (TRAP)	[11]

		IC_{50} = 89.82 ± 1.76 (NET)<br>VITAMIN C<br>IC_{50} =45.41 ± 1.80 μM (DPPH)<br>IC_{50} = 105.47 ± 3.22 μM (ABTS)<br>IC_{50} = 102.22 ± 2.44 (TRAP)<br>IC_{50} = 167.31 ± 1.54 (NET)	
**179**	IC_{50} = 19.71 ± 1.21μM (DPPH)<br>IC_{50} = 91.33 ± 1.84μM (ABTS)<br>IC_{50} = 82.00 ± 2.75 (TRAP)<br>IC_{50} = 119.43 ± 1.96 (NET)	CURCUMIN<br>IC_{50} =13.53 ± 0.50 μM (DPPH)<br>IC_{50} = 48.63 ± 2.13 μM (ABTS)<br>IC_{50} = 54.35 ± 1.95 (TRAP)<br>IC_{50} = 89.82 ± 1.76 (NET)<br>VITAMIN C<br>IC_{50} =45.41 ± 1.80 μM (DPPH)<br>IC_{50} = 105.47 ± 3.22 μM (ABTS)<br>IC_{50} = 102.22 ± 2.44 (TRAP)<br>IC_{50} = 167.31 ± 1.54 (NET)	[11]
**180**	IC_{50} = 34.38 ± 2.52μM (DPPH)<br>IC_{50} = 99.51 ± 2.62μM (ABTS)<br>IC_{50} =118.39 ± 1.88 (TRAP)<br>IC_{50} = 164.35 ± 1.28 (NET)	CURCUMIN<br>IC_{50} =13.53 ± 0.50 μM (DPPH)<br>IC_{50} = 48.63 ± 2.13 μM (ABTS)<br>IC_{50} = 54.35 ± 1.95 (TRAP)<br>IC_{50} = 89.82 ± 1.76 (NET)<br>VITAMIN C<br>IC_{50} =45.41 ± 1.80 μM (DPPH)<br>IC_{50} = 105.47 ± 3.22 μM (ABTS)<br>IC_{50} = 102.22 ± 2.44 (TRAP)<br>IC_{50} = 167.31 ± 1.54 (NET)	[11]
**181**	IC_{50} = 46.33 ± 2.22μM (DPPH)<br>IC_{50} = 105.22 ± 1.17μM (ABTS)<br>IC_{50} = 123.00 ± 3.79 (TRAP)<br>IC_{50} = 169.45 ± 2.49 (NET)	CURCUMIN<br>IC_{50} =13.53 ± 0.50 μM (DPPH)<br>IC_{50} = 48.63 ± 2.13 μM (ABTS)<br>IC_{50} = 54.35 ± 1.95 (TRAP)<br>IC_{50} = 89.82 ± 1.76 (NET)<br>VITAMIN C<br>IC_{50} =45.41 ± 1.80 μM (DPPH)<br>IC_{50} = 105.47 ± 3.22 μM (ABTS)<br>IC_{50} = 102.22 ± 2.44 (TRAP)<br>IC_{50} = 167.31 ± 1.54 (NET)	[11]
**182**	IC_{50} = 28.65 ± 1.91μM (DPPH)<br>IC_{50} = 85.69 ± 1.48μM (ABTS)<br>IC_{50} = 96.35 ± 2.50 (TRAP)<br>IC_{50} = 123.67 ± 1.60 (NET)	CURCUMIN<br>IC_{50} =13.53 ± 0.50 μM (DPPH)<br>IC_{50} = 48.63 ± 2.13 μM (ABTS)<br>IC_{50} = 54.35 ± 1.95 (TRAP)<br>IC_{50} = 89.82 ± 1.76 (NET)<br>VITAMIN C<br>IC_{50} =45.41 ± 1.80 μM (DPPH)<br>IC_{50} = 105.47 ± 3.22 μM (ABTS)<br>IC_{50} = 102.22 ± 2.44 (TRAP)<br>IC_{50} = 167.31 ± 1.54 (NET)	[11]
**183**	IC_{50} = 31.57 ± 2.01μM (DPPH)<br>IC_{50} =80.63 ± 2.26 μM (ABTS)<br>IC_{50} =97.15 ± 3.46 (TRAP)<br>IC_{50} =122.09 ± 1.79 (NET)	CURCUMIN<br>IC_{50} =13.53 ± 0.50 μM (DPPH)<br>IC_{50} = 48.63 ± 2.13 μM (ABTS)<br>IC_{50} = 54.35 ± 1.95 (TRAP)<br>IC_{50} = 89.82 ± 1.76 (NET)<br>VITAMIN C	[11]

		IC_{50} =45.41 ± 1.80 μM (DPPH)<br>IC_{50} = 105.47 ± 3.22 μM (ABTS)<br>IC_{50} = 102.22 ± 2.44 (TRAP)<br>IC_{50} = 167.31 ± 1.54 (NET)	
**184**	IC_{50} = 202.94 ± 1.62μM (DPPH)<br>IC_{50} =132.12 ± 0.83 μM (ABTS)<br>IC_{50} = 286.32 ± 3.44 (TRAP)<br>IC_{50} = 207.34 ± 1.34 (NET)	CURCUMIN<br>IC_{50} =13.53 ± 0.50 μM (DPPH)<br>IC_{50} = 48.63 ± 2.13 μM (ABTS)<br>IC_{50} = 54.35 ± 1.95 (TRAP)<br>IC_{50} = 89.82 ± 1.76 (NET)<br>VITAMIN C<br>IC_{50} =45.41 ± 1.80 μM (DPPH)<br>IC_{50} = 105.47 ± 3.22 μM (ABTS)<br>IC_{50} = 102.22 ± 2.44 (TRAP)<br>IC_{50} = 167.31 ± 1.54 (NET)	[11]
**185**	IC_{50} = 94.15 ± 2.62μM (DPPH)<br>IC_{50} =292.32 ± 1.31 μM (ABTS)<br>IC_{50} = 182.12 ± 1.43 (TRAP)<br>IC_{50} = 231.20 ± 3.42 (NET)	CURCUMIN<br>IC_{50} =13.53 ± 0.50 μM (DPPH)<br>IC_{50} = 48.63 ± 2.13 μM (ABTS)<br>IC_{50} = 54.35 ± 1.95 (TRAP)<br>IC_{50} = 89.82 ± 1.76 (NET)<br>VITAMIN C<br>IC_{50} =45.41 ± 1.80 μM (DPPH)<br>IC_{50} = 105.47 ± 3.22 μM (ABTS)<br>IC_{50} = 102.22 ± 2.44 (TRAP)<br>IC_{50} = 167.31 ± 1.54 (NET)	[11]
**186**	IC_{50} = 94.62 ± 3.26μM (DPPH)<br>IC_{50} = 114.00 ± 2.06μM (ABTS)<br>IC_{50} =184.03 ± 2.16 (TRAP)<br>IC_{50} =214.42 ± 0.95 (NET)	CURCUMIN<br>IC_{50} =13.53 ± 0.50 μM (DPPH)<br>IC_{50} = 48.63 ± 2.13 μM (ABTS)<br>IC_{50} = 54.35 ± 1.95 (TRAP)<br>IC_{50} = 89.82 ± 1.76 (NET)<br>VITAMIN C<br>IC_{50} =45.41 ± 1.80 μM (DPPH)<br>IC_{50} = 105.47 ± 3.22 μM (ABTS)<br>IC_{50} = 102.22 ± 2.44 (TRAP)<br>IC_{50} = 167.31 ± 1.54 (NET)	[11]
**187**	IC_{50} = 32.56 ± 2.47μM (DPPH)<br>IC_{50} = 113.05 ± 1.94μM (ABTS)<br>IC_{50} = 113.02 ± 4.28 (TRAP)<br>IC_{50} = 156.32 ± 3.17 (NET)	CURCUMIN<br>IC_{50} =13.53 ± 0.50 μM (DPPH)<br>IC_{50} = 48.63 ± 2.13 μM (ABTS)<br>IC_{50} = 54.35 ± 1.95 (TRAP)<br>IC_{50} = 89.82 ± 1.76 (NET)<br>VITAMIN C<br>IC_{50} =45.41 ± 1.80 μM (DPPH)<br>IC_{50} = 105.47 ± 3.22 μM (ABTS)<br>IC_{50} = 102.22 ± 2.44 (TRAP)<br>IC_{50} = 167.31 ± 1.54 (NET)	[11]
**188**	IC_{50} =13.51 ± 2.21 μM (DPPH)<br>IC_{50} = 36.34 ± 2.06μM (ABTS)<br>IC_{50} =47.80 ± 2.19 (TRAP)<br>IC_{50} = 80.93 ± 2.42 (NET)	CURCUMIN<br>IC_{50} =13.53 ± 0.50 μM (DPPH)<br>IC_{50} = 48.63 ± 2.13 μM (ABTS)<br>IC_{50} = 54.35 ± 1.95 (TRAP)<br>IC_{50} = 89.82 ± 1.76 (NET)<br>VITAMIN C<br>IC_{50} =45.41 ± 1.80 μM (DPPH)<br>IC_{50} = 105.47 ± 3.22 μM (ABTS)	[11]

		IC_{50} = 102.22 ± 2.44 (TRAP)<br>IC_{50} = 167.31 ± 1.54 (NET)	
**189**	IC_{50} = 28.09 ± 4.46μM (DPPH)<br>IC_{50} = 75.08 ± 1.84μM (ABTS)<br>IC_{50} = 70.00 ± 3.40 (TRAP)<br>IC_{50} =117.24 ± 3.47 (NET)	CURCUMIN<br>IC_{50} =13.53 ± 0.50 μM (DPPH)<br>IC_{50} = 48.63 ± 2.13 μM (ABTS)<br>IC_{50} = 54.35 ± 1.95 (TRAP)<br>IC_{50} = 89.82 ± 1.76 (NET)<br>VITAMIN C<br>IC_{50} =45.41 ± 1.80 μM (DPPH)<br>IC_{50} = 105.47 ± 3.22 μM (ABTS)<br>IC_{50} = 102.22 ± 2.44 (TRAP)<br>IC_{50} = 167.31 ± 1.54 (NET)	[11]
**190**	14.0 ± 2.4% (DPPH at 200 μM)<br>0.015 mmol AAE/L (FRAP)	CURCUMIN<br>84.2 ± 1.6% (DPPH at 200 μM)<br>0.803 mmol AAE/L (FRAP)	[12]
**191**	13.6 ± 2.3% (DPPH at 200 μM)<br>0.024 mmol AAE/L (FRAP)	CURCUMIN<br>84.2 ± 1.6% (DPPH at 200 μM)<br>0.803 mmol AAE/L (FRAP)	[12]
**192**	21.8 ± 1.5% (DPPH at 200 μM)<br>0.018 mmol AAE/L (FRAP)	CURCUMIN<br>84.2 ± 1.6% (DPPH at 200 μM)<br>0.803 mmol AAE/L (FRAP)	[12]
**193**	20.1 ± 1.7% (DPPH at 200 μM)<br>0.032 mmol AAE/L (FRAP)	CURCUMIN<br>84.2 ± 1.6% (DPPH at 200 μM)<br>0.803 mmol AAE/L (FRAP)	[12]
**194**	17.1 ± 3.2% (DPPH at 200 μM)<br>0.011 mmol AAE/L (FRAP)	CURCUMIN<br>84.2 ± 1.6% (DPPH at 200 μM)<br>0.803 mmol AAE/L (FRAP)	[12]
**195**	25.0 ± 1.2% (DPPH at 200 μM)<br>0.008 mmol AAE/L (FRAP)	CURCUMIN<br>84.2 ± 1.6% (DPPH at 200 μM)<br>0.803 mmol AAE/L (FRAP)	[12]
**196**	14.0 ± 1.7% (DPPH at 200 μM)<br>0.001 mmol AAE/L (FRAP)	CURCUMIN<br>84.2 ± 1.6% (DPPH at 200 μM)<br>0.803 mmol AAE/L (FRAP)	[12]
**197**	17.3 ± 1.8% (DPPH at 200 μM)<br>0.042 mmol AAE/L (FRAP)	CURCUMIN<br>84.2 ± 1.6% (DPPH at 200 μM)<br>0.803 mmol AAE/L (FRAP)	[12]
**198**	15.5 ± 3.2% (DPPH at 200 μM)<br>0.080 mmol AAE/L (FRAP)	CURCUMIN<br>84.2 ± 1.6% (DPPH at 200 μM)<br>0.803 mmol AAE/L (FRAP)	[12]
**199**	85.1 ± 1.3% (DPPH at 200 μM)<br>IC_{50} = 11.5 ± 0.4 μM (DPPH)<br>0.756 mmol AAE/L (FRAP)	CURCUMIN<br>84.2 ± 1.6% (DPPH at 200 μM)<br>IC_{50} = 23.6 ± 1.1 μM (DPPH)<br>0.803 mmol AAE/L (FRAP)	[12]
**200**	17.8 ± 1.8% (DPPH at 200 μM)<br>0.015 mmol AAE/L (FRAP)	CURCUMIN<br>84.2 ± 1.6% (DPPH at 200 μM)<br>0.803 mmol AAE/L (FRAP)	[12]
**201**	11.7 ± 1.4% (DPPH at 200 μM)<br>0.073 mmol AAE/L (FRAP)	CURCUMIN<br>84.2 ± 1.6% (DPPH at 200 μM)<br>0.803 mmol AAE/L (FRAP)	[12]
**202**	9.9 ± 1.2% (DPPH at 200 μM)<br>0.034 mmol AAE/L (FRAP)	CURCUMIN<br>84.2 ± 1.6% (DPPH at 200 μM)<br>0.803 mmol AAE/L (FRAP)	[12]

**203**	5.1 ± 4.2% (DPPH at 200 μM)<br>0.178 mmol AAE/L (FRAP)	CURCUMIN<br>84.2 ± 1.6% (DPPH at 200 μM)<br>0.803 mmol AAE/L (FRAP)	[12]
**204**	7.7 ± 1.2% (DPPH at 200 μM)<br>0.237 mmol AAE/L (FRAP)	CURCUMIN<br>84.2 ± 1.6% (DPPH at 200 μM)<br>0.803 mmol AAE/L (FRAP)	[12]
**205**	8.3 ± 2.9% (DPPH at 200 μM)<br>0.038 mmol AAE/L (FRAP)	CURCUMIN<br>84.2 ± 1.6% (DPPH at 200 μM)<br>0.803 mmol AAE/L (FRAP)	[12]
**206**	67.5 ± 1.1% (DPPH at 200 μM)<br>IC_{50} = 47.6 ± 2.1 μM (DPPH)<br>0.379 mmol AAE/L (FRAP)	CURCUMIN<br>84.2 ± 1.6% (DPPH at 200 μM)<br>IC_{50} = 23.6 ± 1.1 μM (DPPH)<br>0.803 mmol AAE/L (FRAP)	[12]
**207**	15.4 ± 2.3% (DPPH at 200 μM)<br>0.079 mmol AAE/L (FRAP)	CURCUMIN<br>84.2 ± 1.6% (DPPH at 200 μM)<br>0.803 mmol AAE/L (FRAP)	[12]
**208**	18.0 ± 2.0% (DPPH at 200 μM)<br>0.086 mmol AAE/L (FRAP)	CURCUMIN<br>84.2 ± 1.6% (DPPH at 200 μM)<br>0.803 mmol AAE/L (FRAP)	[12]
**209**	27.0 ± 1.9% (DPPH at 200 μM)<br>0.177 mmol AAE/L (FRAP)	CURCUMIN<br>84.2 ± 1.6% (DPPH at 200 μM)<br>0.803 mmol AAE/L (FRAP)	[12]
**210**	21.8 ± 2.1% (DPPH at 200 μM)<br>0.177 mmol AAE/L (FRAP)	CURCUMIN<br>84.2 ± 1.6% (DPPH at 200 μM)<br>0.803 mmol AAE/L (FRAP)	[12]
**211**	18.4 ± 2.0% (DPPH at 200 μM)<br>0.140 mmol AAE/L (FRAP)	CURCUMIN<br>84.2 ± 1.6% (DPPH at 200 μM)<br>0.803 mmol AAE/L (FRAP)	[12]
**212**	23.4 ± 2.3% (DPPH at 200 μM)<br>0.018 mmol AAE/L (FRAP)	CURCUMIN<br>84.2 ± 1.6% (DPPH at 200 μM)<br>0.803 mmol AAE/L (FRAP)	[12]
**213**	8.0 ± 3.4% (DPPH at 200 μM)<br>0.131 mmol AAE/L (FRAP)	CURCUMIN<br>84.2 ± 1.6% (DPPH at 200 μM)<br>0.803 mmol AAE/L (FRAP)	[12]
**ANTI-INFLAMMATORY ACTIVITY**			
**Effect of diarylpentanoids on NO production**			
**Compound**		**Reference Drug**	**Reference**
**28**	IC_{50} = 42.01 ± 1.19 μM	CURCUMIN<br>IC_{50} = 20.38 ± 0.28 μM<br>L-NAME<br>IC_{50} = 27.73 ± 5.58 μM	[13]
**125**	IC_{50} = 10.24 ± 1.05 μM	CURCUMIN<br>IC_{50} = 20.38 ± 0.28 μM<br>L-NAME<br>IC_{50} = 27.73 ± 5.58 μM	[13]
**214**	IC_{50} = 13.64 ± 0.41 μM	CURCUMIN<br>IC_{50} = 20.38 ± 0.28 μM<br>L-NAME<br>IC_{50} = 27.73 ± 5.58 μM	[13]
**215**	IC_{50} = 33.13 ± 9.28 μM	CURCUMIN<br>IC_{50} = 20.38 ± 0.28 μM<br>L-NAME	[13]

		IC_{50} = 27.73 ± 5.58 μM	
**216**	IC_{50} = 42.04 ± 4.72 μM	CURCUMIN<br>IC_{50} = 20.38 ± 0.28 μM<br>L-NAME<br>IC_{50} = 27.73 ± 5.58 μM	[13]
**217**	IC_{50} = 36.95 ± 2.87 μM	CURCUMIN<br>IC_{50} = 20.38 ± 0.28 μM<br>L-NAME<br>IC_{50} = 27.73 ± 5.58 μM	[13]
**218**	IC_{50} = 13.66 ± 0.61 μM	CURCUMIN<br>IC_{50} = 20.38 ± 0.28 μM<br>L-NAME<br>IC_{50} = 27.73 ± 5.58 μM	[13]
**22**	57.77% (inhibhiton of NO production at 200 μg/mL)	L-NAME (Nω- nitro-L-arginine methyl O esther hydrochloride<br>82.84% (inhibhiton of NO production at 200 μg/mL)	[8]
**163**	32.62% (inhibhiton of NO production at 200 μg/mL)	L-NAME<br>82.84% (inhibhiton of NO production at 200 μg/mL)	[8]
**164**	58.64% (inhibhiton of NO production at 200 μg/mL)	L-NAME<br>82.84% (inhibhiton of NO production at 200 μg/mL)	[8]
**219**	IC_{50} = 22.6 ± 0.5 μM (NO inhibition)	CURCUMIN<br>IC_{50} = 14.7 ± 0.2 μM (NO inhibition)	[14]
**220**	IC_{50} = 27.3 ± 0.2 μM (Inhibiton of NO production)	CURCUMIN<br>IC_{50} = 14.7 ± 0.2 μM (Inhibiton of NO production)	[14]
**221**	IC_{50} = 26.7 ± 0.7 μM (Inhibiton of NO production)	CURCUMIN<br>IC_{50} = 14.7 ± 0.2 μM (Inhibiton of NO production)	[14]
**222**	IC_{50} = 29.4 ± 0.7 μM (Inhibiton of NO production)	CURCUMIN<br>IC_{50} = 14.7 ± 0.2 μM (Inhibiton of NO production)	[14]
**223**	IC_{50} = 25.7 ± 0.6μM (Inhibiton of NO production)	CURCUMIN<br>IC_{50} = 14.7 ± 0.2 μM (Inhibiton of NO production)	[14]
**224**	IC_{50} = 31.6 ± 0.6 μM (Inhibiton of NO production)	CURCUMIN<br>IC_{50} = 14.7 ± 0.2 μM (Inhibiton of NO production)	[14]
**225**	IC_{50} = 24.6 ± 0.7 μM (Inhibiton of NO production)	CURCUMIN<br>IC_{50} = 14.7 ± 0.2 μM (Inhibiton of NO production)	[14]
**226**	IC_{50} = 16.6 ± 1.1 μM (Inhibiton of NO production)	CURCUMIN<br>IC_{50} = 14.7 ± 0.2 μM (Inhibiton of NO production)	[14]
**227**	IC_{50} = 49.0 ± 1.0 μM (Inhibiton of NO production)	CURCUMIN<br>IC_{50} = 14.7 ± 0.2 μM (Inhibiton of NO production)	[14]
**228**	IC_{50} = 28.4 ± 0.4 μM (Inhibiton of NO production)	CURCUMIN<br>IC_{50} = 14.7 ± 0.2 μM (Inhibiton of NO production)	[14]

**229**	IC_{50} = 42.6 ± 0.1 μM (Inhibiton of NO production)	CURCUMIN IC_{50} = 14.7 ± 0.2 μM (Inhibiton of NO production)	[14]
**230**	IC_{50} = 36.4 ± 1.8 μM (Inhibiton of NO production)	CURCUMIN IC_{50} = 14.7 ± 0.2 μM (Inhibiton of NO production)	[14]
**231**	IC_{50} = 39.6 ± 4.1 μM (Inhibiton of NO production)	CURCUMIN IC_{50} = 14.7 ± 0.2 μM (Inhibiton of NO production)	[14]
**232**	IC_{50} = 23.0 ± 1.7 μM (Inhibiton of NO production)	CURCUMIN IC_{50} = 14.7 ± 0.2 μM (Inhibiton of NO production)	[14]
**233**	IC_{50} = 47.6 ± 1.1 μM (Inhibiton of NO production)	CURCUMIN IC_{50} = 14.7 ± 0.2 μM (Inhibiton of NO production)	[14]
**234**	IC_{50} = 30.0 ± 2.1 μM (Inhibiton of NO production)	CURCUMIN IC_{50} = 14.7 ± 0.2 μM (Inhibiton of NO production)	[14]
**235**	IC_{50} = 36.2 ± 0.9 μM (Inhibiton of NO production)	CURCUMIN IC_{50} = 14.7 ± 0.2 μM (Inhibiton of NO production)	[14]
**236**	IC_{50} = 36.0 ± 0.5 μM (Inhibiton of NO production)	CURCUMIN IC_{50} = 14.7 ± 0.2 μM (Inhibiton of NO production)	[14]
**237**	IC_{50} = 17.4 ± 0.4 μM (Inhibiton of NO production)	CURCUMIN IC_{50} = 14.7 ± 0.2 μM (Inhibiton of NO production)	[14]
**238**	IC_{50} = 13.6 ± 0.5 μM (Inhibiton of NO production)	CURCUMIN IC_{50} = 14.7 ± 0.2 μM (Inhibiton of NO production)	[14]
**239**	IC_{50} = 32.2 ± 0.2 μM (Inhibiton of NO production)	CURCUMIN IC_{50} = 14.7 ± 0.2 μM (Inhibiton of NO production)	[14]
**240**	IC_{50} = 25.3 ± 1.7 μM (Inhibiton of NO production)	CURCUMIN IC_{50} = 14.7 ± 0.2 μM (Inhibiton of NO production)	[14]
**241**	IC_{50} = 62.4 ± 2.3 μM (Inhibiton of NO production)	CURCUMIN IC_{50} = 14.7 ± 0.2 μM (Inhibiton of NO production)	[14]
**242**	IC_{50} = 25.5 ± 1.0 μM (Inhibiton of NO production)	CURCUMIN IC_{50} = 14.7 ± 0.2 μM (Inhibiton of NO production)	[14]
**243**	IC_{50} = 20.8 ± 0.5 μM (Inhibiton of NO production)	CURCUMIN IC_{50} = 14.7 ± 0.2 μM (Inhibiton of NO production)	[14]
**244**	IC_{50} = 19.8 ± 0.9 μM (Inhibiton of NO production)	CURCUMIN IC_{50} = 14.7 ± 0.2 μM (Inhibiton of NO production)	[14]
**245**	IC_{50} = 29.5 ± 0.5 μM (Inhibiton of NO production)	CURCUMIN IC_{50} = 14.7 ± 0.2 μM (Inhibiton of NO production)	[14]

**246**	IC_{50} = 58.5 ± 2.5 μM (Inhibiton of NO production)	CURCUMIN<br>IC_{50} = 14.7 ± 0.2 μM (Inhibiton of NO production)	[14]
**247**	IC_{50} = 39.0 ± 1.2 μM (Inhibiton of NO production)	CURCUMIN<br>IC_{50} = 14.7 ± 0.2 μM (Inhibiton of NO production)	[14]
**248**	IC_{50} = 45.8 ± 0.8 μM (Inhibiton of NO production)	CURCUMIN<br>IC_{50} = 14.7 ± 0.2 μM (Inhibiton of NO production)	[14]
**249**	IC_{50} = 21.7 ± 0.5 μM (Inhibiton of NO production)	CURCUMIN<br>IC_{50} = 14.7 ± 0.2 μM (Inhibiton of NO production)	[14]
**250**	IC_{50} = 29.3 ± 0.3 μM (Inhibiton of NO production)	CURCUMIN<br>IC_{50} = 14.7 ± 0.2 μM (Inhibiton of NO production)	[14]
**251**	IC_{50} = 25.9 ± 0.3 μM (Inhibiton of NO production)	CURCUMIN<br>IC_{50} = 14.7 ± 0.2 μM (Inhibiton of NO production)	[14]
**252**	IC_{50} = 35.7 ± 0.8 μM (Inhibiton of NO production)	CURCUMIN<br>IC_{50} = 14.7 ± 0.2 μM (Inhibiton of NO production)	[14]
**253**	IC_{50} = 27.4 ± 0.2 μM (Inhibiton of NO production)	CURCUMIN<br>IC_{50} = 14.7 ± 0.2 μM (Inhibiton of NO production)	[14]
**254**	IC_{50} = 35.1 ± 0.2 μM (Inhibiton of NO production)	CURCUMIN<br>IC_{50} = 14.7 ± 0.2 μM (Inhibiton of NO production)	[14]
**255**	IC_{50} = 19.8 ± 0.4 μM (Inhibiton of NO production)	CURCUMIN<br>IC_{50} = 14.7 ± 0.2 μM (Inhibiton of NO production)	[14]
**256**	IC_{50} = 18.4 ± 0.2 μM (Inhibiton of NO production)	CURCUMIN<br>IC_{50} = 14.7 ± 0.2 μM (Inhibiton of NO production)	[14]
**257**	IC_{50} = 71.5 ± 2.5μM (Inhibiton of NO production)	CURCUMIN<br>IC_{50} = 14.7 ± 0.2 μM (Inhibiton of NO production)	[14]
**258**	IC_{50} = 33.4 ± 0.7 μM (Inhibiton of NO production)	CURCUMIN<br>IC_{50} = 14.7 ± 0.2 μM (Inhibiton of NO production)	[14]
**259**	IC_{50} = 35.6 ± 0.6 μM (Inhibiton of NO production)	CURCUMIN<br>IC_{50} = 14.7 ± 0.2 μM (Inhibiton of NO production)	[14]
**260**	IC_{50} = 28.9 ± 1.5μM (Inhibiton of NO production)	CURCUMIN<br>IC_{50} = 14.7 ± 0.2 μM (Inhibiton of NO production)	[14]
**261**	IC_{50} = 30.8 ± 0.7 μM (Inhibiton of NO production)	CURCUMIN<br>IC_{50} = 14.7 ± 0.2 μM (Inhibiton of NO production)	[14]
**262**	IC_{50} = 19.1 ± 0.6 μM (Inhibiton of NO production)	CURCUMIN<br>IC_{50} = 14.7 ± 0.2 μM (Inhibiton of NO production)	[14]

**263**	IC_{50} = 16.7 ± 0.6 μM (Inhibiton of NO production)	CURCUMIN<br>IC_{50} = 14.7 ± 0.2 μM (Inhibiton of NO production)	[14]
**264**	IC_{50} = 15.9 ± 0.9 μM (Inhibiton of NO production)	CURCUMIN<br>IC_{50} = 14.7 ± 0.2 μM (Inhibiton of NO production)	[14]
**265**	IC_{50} = 26.9 ± 0.8 μM (Inhibiton of NO production)	CURCUMIN<br>IC_{50} = 14.7 ± 0.2 μM (Inhibiton of NO production)	[14]
**266**	IC_{50} = 18.7 ± 0.8 μM (Inhibiton of NO production)	CURCUMIN<br>IC_{50} = 14.7 ± 0.2 μM (Inhibiton of NO production)	[14]
**267**	IC_{50} = 32.3 ± 0.9 μM (Inhibiton of NO production)	CURCUMIN<br>IC_{50} = 14.7 ± 0.2 μM (Inhibiton of NO production)	[14]
**268**	IC_{50} = 4.9 ± 0.3 μM (Inhibiton of NO production)	CURCUMIN<br>IC_{50} = 14.7 ± 0.2 μM (Inhibiton of NO production)	[14]
**269**	IC_{50} = 31.0 ± 2.0 μM (Inhibiton of NO production)	CURCUMIN<br>IC_{50} = 14.7 ± 0.2 μM (Inhibiton of NO production)	[14]
**270**	IC_{50} = 35.7 ± 0. 9 μM (Inhibiton of NO production)	CURCUMIN<br>IC_{50} = 14.7 ± 0.2 μM (Inhibiton of NO production)	[14]
**271**	IC_{50} = 75.0 ± 4.9 μM (Inhibiton of NO production)	CURCUMIN<br>IC_{50} = 14.7 ± 0.2 μM (Inhibiton of NO production)	[14]
**272**	IC_{50} = 29.9 ± 0.4 μM (Inhibiton of NO production)	CURCUMIN<br>IC_{50} = 14.7 ± 0.2 μM (Inhibiton of NO production)	[14]
**273**	IC_{50} = 24.8 ± 0.4 μM (Inhibiton of NO production)	CURCUMIN<br>IC_{50} = 14.7 ± 0.2 μM (Inhibiton of NO production)	[14]
**274**	IC_{50} =44.8 ± 1.6 μM (Inhibiton of NO production)	CURCUMIN<br>IC_{50} = 14.7 ± 0.2 μM (Inhibiton of NO production)	[14]
**275**	IC_{50} =9.6 ± 0.5 μM (Inhibiton of NO production)	CURCUMIN<br>IC_{50} = 14.7 ± 0.2 μM (Inhibiton of NO production)	[14]
**190**	5.3 ± 2.7% (Inhibiton of NO production at 50 μM)	CURCUMIN<br>99.3 ± 0.2% (Inhibiton of NO production at 50 μM)<br>IC_{50} = 14.7 ± 0.2 μM (Inhibiton of NO production)	[12]
**191**	6.9 ± 5.7% (Inhibiton of NO production at 50 μM)	CURCUMIN<br>99.3 ± 0.2% (Inhibiton of NO production at 50 μM)<br>IC_{50} = 14.7 ± 0.2 μM (Inhibiton of NO production)	[12]
**192**	71.6 ± 0.9% (Inhibiton of NO production at 50 μM)	CURCUMIN<br>99.3 ± 0.2% (Inhibiton of NO production at 50 μM)	[12]

	IC_{50} = 23.3 ± 0.6 μM (Inhibiton of NO production)	IC_{50} = 14.7 ± 0.2 μM (Inhibiton of NO production)	
**193**	30.8 ± 5.1% (Inhibiton of NO production at 50 μM)	CURCUMIN<br>99.3 ± 0.2% (Inhibiton of NO production at 50 μM)<br>IC_{50} = 14.7 ± 0.2 μM (Inhibiton of NO production)	[12]
**194**	21.8 ± 2.8% (Inhibiton of NO production at 50 μM)	CURCUMIN<br>99.3 ± 0.2% (Inhibiton of NO production at 50 μM)<br>IC_{50} = 14.7 ± 0.2 μM (Inhibiton of NO production)	[12]
**195**	34.5 ± 5.4% (Inhibiton of NO production at 50 μM)	CURCUMIN<br>99.3 ± 0.2% (Inhibiton of NO production at 50 μM)<br>IC_{50} = 14.7 ± 0.2 μM (Inhibiton of NO production)	[12]
**196**	2.0 ± 1.7% (Inhibiton of NO production at 50 μM)	CURCUMIN<br>99.3 ± 0.2% (Inhibiton of NO production at 50 μM)<br>IC_{50} = 14.7 ± 0.2 μM (Inhibiton of NO production)	[12]
**197**	80.0 ± 1.5% (Inhibiton of NO production at 50 μM)<br>IC_{50} = 4.2 ± 0.2 μM (Inhibiton of NO production)	CURCUMIN<br>99.3 ± 0.2% (Inhibiton of NO production at 50 μM)<br>IC_{50} = 14.7 ± 0.2 μM (Inhibiton of NO production)	[12]
**198**	25.6 ± 2.9% (Inhibiton of NO production at 50 μM)	CURCUMIN<br>99.3 ± 0.2% (Inhibiton of NO production at 50 μM)<br>IC_{50} = 14.7 ± 0.2 μM (Inhibiton of NO production)	[12]
**199**	86.0 ± 2.0% (Inhibiton of NO production at 50 μM)<br>IC_{50} = 35.3 ± 1.4 μM (Inhibiton of NO production)	CURCUMIN<br>99.3 ± 0.2% (Inhibiton of NO production at 50 μM)<br>IC_{50} = 14.7 ± 0.2 μM (Inhibiton of NO production)	[12]
**200**	15.0 ± 2.2% (Inhibiton of NO production at 50 μM)	CURCUMIN<br>99.3 ± 0.2% (Inhibiton of NO production at 50 μM)<br>IC_{50} = 14.7 ± 0.2 μM (Inhibiton of NO production)	[12]
**201**	9.3 ± 4.9% (Inhibiton of NO production at 50 μM)	CURCUMIN<br>99.3 ± 0.2% (Inhibiton of NO production at 50 μM)<br>IC_{50} = 14.7 ± 0.2 μM (Inhibiton of NO production)	[12]
**202**	29.8 ± 7.4% (Inhibiton of NO production at 50 μM)	CURCUMIN<br>99.3 ± 0.2% (Inhibiton of NO production at 50 μM)<br>IC_{50} = 14.7 ± 0.2 μM (Inhibiton of NO production)	[12]

**203**	3.7 ± 1.8% (Inhibiton of NO production at 50 μM)	CURCUMIN<br>99.3 ± 0.2% (Inhibiton of NO production at 50 μM)<br>IC_{50} = 14.7 ± 0.2 μM (Inhibiton of NO production)	[12]
**204**	8.2 ± 2.2% (Inhibiton of NO production at 50 μM)	CURCUMIN<br>99.3 ± 0.2% (Inhibiton of NO production at 50 μM)<br>IC_{50} = 14.7 ± 0.2 μM (Inhibiton of NO production)	[12]
**205**	15.8 ± 2.4% (Inhibiton of NO production at 50 μM)	CURCUMIN<br>99.3 ± 0.2% (Inhibiton of NO production at 50 μM)<br>IC_{50} = 14.7 ± 0.2 μM (Inhibiton of NO production)	[12]
**206**	69.0 ± 2.6% (Inhibiton of NO production at 50 μM)<br>IC_{50} = 22.7 ± 0.5 μM (Inhibiton of NO production)	CURCUMIN<br>99.3 ± 0.2% (Inhibiton of NO production at 50 μM)<br>IC_{50} = 14.7 ± 0.2 μM (Inhibiton of NO production)	[12]
**207**	73.4 ± 4.9% (Inhibiton of NO production at 50 μM)<br>IC_{50} = 15.2 ± 0.8 μM (Inhibiton of NO production)	CURCUMIN<br>99.3 ± 0.2% (Inhibiton of NO production at 50 μM)<br>IC_{50} = 14.7 ± 0.2 μM (Inhibiton of NO production)	[12]
**207**	83.5 ± 3.0% (Inhibiton of NO production at 50 μM)<br>IC_{50} = 44.6 ± 2.5 μM (Inhibiton of NO production)	CURCUMIN<br>99.3 ± 0.2% (Inhibiton of NO production at 50 μM)<br>IC_{50} = 14.7 ± 0.2 μM (Inhibiton of NO production)	[12]
**209**	15.6 ± 4.7% (Inhibiton of NO production at 50 μM)	CURCUMIN<br>99.3 ± 0.2% (Inhibiton of NO production at 50 μM)<br>IC_{50} = 14.7 ± 0.2 μM (Inhibiton of NO production)	[12]
**210**	8.2 ± 3.5% (Inhibiton of NO production at 50 μM)	CURCUMIN<br>99.3 ± 0.2% (Inhibiton of NO production at 50 μM)<br>IC_{50} = 14.7 ± 0.2 μM (Inhibiton of NO production)	[12]
**211**	7.6 ± 4.0% (Inhibiton of NO production at 50 μM)	CURCUMIN<br>99.3 ± 0.2% (Inhibiton of NO production at 50 μM)<br>IC_{50} = 14.7 ± 0.2 μM (Inhibiton of NO production)	[12]
**212**	8.8 ± 5.0% (Inhibiton of NO production at 50 μM)	CURCUMIN<br>99.3 ± 0.2% (Inhibiton of NO production at 50 μM)<br>IC_{50} = 14.7 ± 0.2 μM (Inhibiton of NO production)	[12]
**213**	9.1 ± 4.0% (Inhibiton of NO production at 50 μM)	CURCUMIN	[12]

		99.3 ± 0.2% (Inhibiton of NO production at 50 µM)<br>IC_{50} = 14.7 ± 0.2 µM (Inhibiton of NO production)	
**276**	66.8 ± 0.7% (Inhibiton of NO production at 50 µM)<br>IC_{50} = 44.6 ± 2.5 µM (Inhibiton of NO production)	CURCUMIN<br>99.3 ± 0.2% (Inhibiton of NO production at 50 µM)<br>IC_{50} = 14.7 ± 0.2 µM (Inhibiton of NO production)	[15]
**277**	< 10% (Inhibiton of NO production at 50 µM)	CURCUMIN<br>99.3 ± 0.2% (Inhibiton of NO production at 50 µM)	[15]
**278**	103.9 ± 0.6% (Inhibiton of NO production at 50 µM)<br>IC_{50} = 26.1 ± 0.1 µM (Inhibiton of NO production)	CURCUMIN<br>99.3 ± 0.2% (Inhibiton of NO production at 50 µM)<br>IC_{50} = 14.7 ± 0.2 µM (Inhibiton of NO production)	[15]
**279**	51.1 ± 4.8% (Inhibiton of NO production at 50 µM)<br>IC_{50} = 71.2 ± 5.1 µM (Inhibiton of NO production)	CURCUMIN<br>99.3 ± 0.2% (Inhibiton of NO production at 50 µM)<br>IC_{50} = 14.7 ± 0.2 µM (Inhibiton of NO production)	[15]
**280**	33.1 ± 4.2% (Inhibiton of NO production at 50 µM)	CURCUMIN<br>99.3 ± 0.2% (Inhibiton of NO production at 50 µM)	[15]
**281**	<10 % (Inhibiton of NO production at 50 µM)	CURCUMIN<br>99.3 ± 0.2% (Inhibiton of NO production at 50 µM)	[15]
**282**	<10% (Inhibiton of NO production at 50 µM)	CURCUMIN<br>99.3 ± 0.2% (Inhibiton of NO production at 50 µM)	[15]
**283**	<10% (Inhibiton of NO production at 50 µM)	CURCUMIN<br>99.3 ± 0.2% (Inhibiton of NO production at 50 µM)	[15]
**284**	19.6 ± 2.1% (Inhibiton of NO production at 50 µM)	CURCUMIN<br>99.3 ± 0.2% (Inhibiton of NO production at 50 µM)	[15]
**285**	52.6 ± 4.3% (Inhibiton of NO production at 50 µM)<br>IC_{50} = 68.6 ± 2.6 µM (Inhibiton of NO production)	CURCUMIN<br>99.3 ± 0.2% (Inhibiton of NO production at 50 µM)<br>IC_{50} = 14.7 ± 0.2 µM (Inhibiton of NO production)	[15]
**286**	84.6 ± 1.1% (Inhibiton of NO production at 50 µM)<br>IC_{50} = 59.0 ± 6.0 µM (Inhibiton of NO production)	CURCUMIN<br>99.3 ± 0.2% (Inhibiton of NO production at 50 µM)<br>IC_{50} = 14.7 ± 0.2 µM (Inhibiton of NO production)	[15]
**287**	73.7 ± 7.1% (Inhibiton of NO production at 50 µM)	CURCUMIN<br>99.3 ± 0.2% (Inhibiton of NO production at 50 µM)	[15]
**288**	77.4 ± 3.4% (Inhibiton of NO production at 50 µM)	CURCUMIN	[15]

	IC_{50} = 45.8 ± 1.5 μM (Inhibiton of NO production)	99.3 ± 0.2% (Inhibiton of NO production at 50 μM)<br>IC_{50} = 14.7 ± 0.2 μM (Inhibiton of NO production)	
**289**	41.6 ± 1.7% (Inhibiton of NO production at 50 μM	CURCUMIN<br>99.3 ± 0.2% (Inhibiton of NO production at 50 μM)	[15]
**290**	40.0 ± 3.3% (Inhibiton of NO production at 50 μM)	CURCUMIN<br>99.3 ± 0.2% (Inhibiton of NO production at 50 μM)	[15]
**291**	26.4 ± 1.7% (Inhibiton of NO production at 50 μM)	CURCUMIN<br>99.3 ± 0.2% (Inhibiton of NO production at 50 μM)	[15]
**292**	<10% (Inhibiton of NO production at 50 μM)	CURCUMIN<br>99.3 ± 0.2% (Inhibiton of NO production at 50 μM)	[15]
**293**	<10% (Inhibiton of NO production at 50 μM)	CURCUMIN<br>99.3 ± 0.2% (Inhibiton of NO production at 50 μM)	[15]
**294**	42.2 ± 2.5% (Inhibiton of NO production at 50 μM)	CURCUMIN<br>99.3 ± 0.2% (Inhibiton of NO production at 50 μM)	[15]
**295**	100.2 ± 2.3% (Inhibiton of NO production at 50 μM)<br>IC_{50} = 16.3 ± 2.0 μM (Inhibiton of NO production)	CURCUMIN<br>99.3 ± 0.2% (Inhibiton of NO production at 50 μM)<br>IC_{50} = 14.7 ± 0.2 μM (Inhibiton of NO production)	[15]
**296**	89.4 ± 3.9% (Inhibiton of NO production at 50 μM)<br>IC_{50} = 21.5 ± 2.3 μM (Inhibiton of NO production)	CURCUMIN<br>99.3 ± 0.2% (Inhibiton of NO production at 50 μM)<br>IC_{50} = 14.7 ± 0.2 μM (Inhibiton of NO production)	[15]
**297**	98.2 ± 0.9% (Inhibiton of NO production at 50 μM)<br>IC_{50} = 23.5 ± 2.4μM (Inhibiton of NO production)	CURCUMIN<br>99.3 ± 0.2% (Inhibiton of NO production at 50 μM)<br>IC_{50} = 14.7 ± 0.2 μM (Inhibiton of NO production)	[15]
**298**	102.3 ± 1.6% (Inhibiton of NO production at 50 μM)<br>IC_{50} = 21.8 ±2.0 μM (Inhibiton of NO production)	CURCUMIN<br>99.3 ± 0.2% (Inhibiton of NO production at 50 μM)<br>IC_{50} = 14.7 ± 0.2 μM (Inhibiton of NO production)	[15]
**299**	41.7 ± 4.6% (Inhibiton of NO production at 50 μM)	CURCUMIN<br>99.3 ± 0.2% (Inhibiton of NO production at 50 μM)	[15]
**300**	59.7 ± 1.1% (Inhibiton of NO production at 50 μM)<br>IC_{50} = 39.8 ± 2.3 μM (Inhibiton of NO production)	CURCUMIN<br>99.3 ± 0.2% (Inhibiton of NO production at 50 μM)<br>IC_{50} = 14.7 ± 0.2 μM (Inhibiton of NO production)	[15]
**301**	44.8 ± 5.2% (Inhibiton of NO production at 50 μM)	CURCUMIN	[15]

		99.3 ± 0.2% (Inhibiton of NO production at 50 μM)	
**302**	95.8 ± 1.7% (Inhibiton of NO production at 50 μM)<br>IC_{50} = 57.4 ± 1.2 μM (Inhibiton of NO production)	CURCUMIN<br>99.3 ± 0.2% (Inhibiton of NO production at 50 μM)<br>IC_{50} = 14.7 ± 0.2 μM (Inhibiton of NO production)	[15]
**303**	69.6 ± 1.1% (Inhibiton of NO production at 50 μM)<br>IC_{50} = 47.9 ± 5.5 μM (Inhibiton of NO production)	CURCUMIN<br>99.3 ± 0.2% (Inhibiton of NO production at 50 μM)<br>IC_{50} = 14.7 ± 0.2 μM (Inhibiton of NO production)	[15]
**304**	61.5 ± 2.4% (Inhibiton of NO production at 50 μM)<br>IC_{50} = 27.5 ± 0.1 μM (Inhibiton of NO production)	CURCUMIN<br>99.3 ± 0.2% (Inhibiton of NO production at 50 μM)<br>IC_{50} = 14.7 ± 0.2 μM (Inhibiton of NO production)	[15]
**305**	51.2 ± 4.2% (Inhibiton of NO production at 50 μM)<br>IC_{50} = 73.6 ± 7.5 μM (Inhibiton of NO production)	CURCUMIN<br>99.3 ± 0.2% (Inhibiton of NO production at 50 μM)<br>IC_{50} = 14.7 ± 0.2 μM (Inhibiton of NO production)	[15]
**306**	54.5 ± 1.3% (Inhibiton of NO production at 50 μM)<br>IC_{50} = 77.4 ± 6.2 μM (Inhibiton of NO production)	CURCUMIN<br>99.3 ± 0.2% (Inhibiton of NO production at 50 μM)<br>IC_{50} = 14.7 ± 0.2 μM (Inhibiton of NO production)	[15]
**307**	16.4 ± 3.1% (Inhibiton of NO production at 50 μM)	CURCUMIN<br>99.3 ± 0.2% (Inhibiton of NO production at 50 μM)	[15]
**308**	71.5 ± 4.8% (Inhibiton of NO production at 50 μM)<br>IC_{50} = 63.8 ± 4.5 μM (Inhibiton of NO production)	CURCUMIN<br>99.3 ± 0.2% (Inhibiton of NO production at 50 μM)<br>IC_{50} = 14.7 ± 0.2 μM (Inhibiton of NO production)	[15]
**309**	32.4 ± 2.8% (Inhibiton of NO production at 50 μM)	CURCUMIN<br>99.3 ± 0.2% (Inhibiton of NO production at 50 μM)	[15]
**310**	56.9 ± 3.5% (Inhibiton of NO production at 50 μM)<br>IC_{50} = 115.9 ± 4.8 μM (Inhibiton of NO production)	CURCUMIN<br>99.3 ± 0.2% (Inhibiton of NO production at 50 μM)<br>IC_{50} = 14.7 ± 0.2 μM (Inhibiton of NO production)	[15]
**31**	31.2 ± 4.0% (Inhibiton of NO production at 50 μM)	CURCUMIN<br>99.3 ± 0.2% (Inhibiton of NO production at 50 μM)	[15]
**312**	45.4 ± 3.9% (Inhibiton of NO production at 50 μM)<br>IC_{50} = 70.5 ± 2.1 μM (Inhibiton of NO production)	CURCUMIN<br>99.3 ± 0.2% (Inhibiton of NO production at 50 μM)<br>IC_{50} = 14.7 ± 0.2 μM (Inhibiton of NO production)	[15]

**313**	<10% (Inhibiton of NO production at 50 μM)	CURCUMIN<br>99.3 ± 0.2% (Inhibiton of NO production at 50 μM)	[15]
**314**	64.1 ± 5.0% (Inhibiton of NO production at 50 μM)<br>IC_{50} = 42.0 ± 1.6 μM (Inhibiton of NO production)	CURCUMIN<br>99.3 ± 0.2% (Inhibiton of NO production at 50 μM)<br>IC_{50} = 14.7 ± 0.2 μM (Inhibiton of NO production)	[15]
**315**	76.7 ± 1.1% (Inhibiton of NO production at 50 μM)<br>IC_{50} = 39.3 ± 0.1 μM (Inhibiton of NO production)	CURCUMIN<br>99.3 ± 0.2% (Inhibiton of NO production at 50 μM)<br>IC_{50} = 14.7 ± 0.2 μM (Inhibiton of NO production)	[15]
**316**	101.8 ± 1.1% (Inhibiton of NO production at 50 μM)<br>IC_{50} = 16.4 ± 0.3 μM (Inhibiton of NO production)	CURCUMIN<br>99.3 ± 0.2% (Inhibiton of NO production at 50 μM)<br>IC_{50} = 14.7 ± 0.2 μM (Inhibiton of NO production)	[15]
**317**	82.1 ± 2.7% (Inhibiton of NO production at 50 μM)<br>IC_{50} = 27.2 ± 1.1 μM (Inhibiton of NO production)	CURCUMIN<br>99.3 ± 0.2% (Inhibiton of NO production at 50 μM)<br>IC_{50} = 14.7 ± 0.2 μM (Inhibiton of NO production)	[15]
**318**	93.4 ± 0.1% (Inhibiton of NO production at 50 μM)<br>IC_{50} = 27.11 ± 0.7 μM (Inhibiton of NO production)	CURCUMIN<br>99.3 ± 0.2% (Inhibiton of NO production at 50 μM)<br>IC_{50} = 14.7 ± 0.2 μM (Inhibiton of NO production)	[15]
**319**	103.1 ± 1.6% (Inhibiton of NO production at 50 μM)<br>IC_{50} = 10.2 ± 0.6 μM (Inhibiton of NO production)	CURCUMIN<br>99.3 ± 0.2% (Inhibiton of NO production at 50 μM)<br>IC_{50} = 14.7 ± 0.2 μM (Inhibiton of NO production)	[15]
**320**	110.2 ± 1.1% (Inhibiton of NO production at 50 μM)<br>IC_{50} = 14.8 ± 1.9 μM (Inhibiton of NO production)	CURCUMIN<br>99.3 ± 0.2% (Inhibiton of NO production at 50 μM)<br>IC_{50} = 14.7 ± 0.2 μM (Inhibiton of NO production)	[15]
**Modulation of pro-inflammatory cytokines and transcription factors**			
**Compound**		**Reference rug**	**Reference**
**3**	Enhancer of the TPA-induced activation of AP-1 at 15 μM	CURCUMIN<br>~55% (Inhibition of the TPA-induced activation of AP-1 at 15 μM)<br>IC_{50} = 12.8 ± 0.5 μM	[16]
**8**	Enhancer of the TPA-induced activation of AP-1 at 15 μM	CURCUMIN<br>~55% (Inhibition of the TPA-induced activation of AP-1 at 15 μM)<br>IC_{50} = 12.8 ± 0.5 μM	[16]
**9**	Enhancer of the TPA-induced activation of AP-1 at 15 μM	CURCUMIN<br>~55% (Inhibition of the TPA-induced activation of AP-1 at 15 μM)<br>IC_{50} = 12.8 ± 0.5 μM	[16]

**34**	Inhibitor of the TPA-induced activation of AP-1 at 15 μM<br>IC_{50} = 7.3 ± 0.4 μM	CURCUMIN<br>~55% (Inhibition of the TPA-induced activation of AP-1 at 15 μM)<br>IC_{50} = 12.8 ± 0.5 μM	[16]
**120**	Enhancer of the TPA-induced activation of AP-1 at 15 μM	CURCUMIN<br>~55% (Inhibition of the TPA-induced activation of AP-1 at 15 μM)<br>IC_{50} = 12.8 ± 0.5 μM	[16]
**122**	Inhibitor of the TPA-induced activation of AP-1 at 15 μM	CURCUMIN<br>~55% (Inhibition of the TPA-induced activation of AP-1 at 15 μM)<br>IC_{50} = 12.8 ± 0.5 μM	[16]
**125**	Inhibitor of the TPA-induced activation of AP-1 at 15 μM<br>IC_{50} = 11.4 ± 1.0 μM	CURCUMIN<br>~55% (Inhibition of the TPA-induced activation of AP-1 at 15 μM)<br>IC_{50} = 12.8 ± 0.5 μM	[16]
**165**	Enhancer of the TPA-induced activation of AP-1 at 15 μM	CURCUMIN<br>~55% (Inhibition of the TPA-induced activation of AP-1 at 15 μM)<br>IC_{50} = 12.8 ± 0.5 μM	[16]
**214**	Inhibitor of the TPA-induced activation of AP-1 at 15 μM<br>IC_{50} = 1.4 ± 0.2 μM	CURCUMIN<br>~55% (Inhibition of the TPA-induced activation of AP-1 at 15 μM)<br>IC_{50} = 12.8 ± 0.5 μM	[16]
**321**	Inhibitor of the TPA-induced activation of AP-1 at 15 μM<br>IC_{50} = 8.3 ± 0.6 μM	CURCUMIN<br>~55% (Inhibition of the TPA-induced activation of AP-1 at 15 μM)<br>IC_{50} = 12.8 ± 0.5 μM	[16]
**322**	Inhibitor of the TPA-induced activation of AP-1 at 15 μM<br>IC_{50} = 4.1 ± 0.02 μM	CURCUMIN<br>~55% (Inhibition of the TPA-induced activation of AP-1 at 15 μM)<br>IC_{50} = 12.8 ± 0.5 μM	[16]
**323**	Inhibitor of the TPA-induced activation of AP-1 at 15 μM<br>IC_{50} = 6.6 ± 0.2 μM	CURCUMIN<br>~55% (Inhibition of the TPA-induced activation of AP-1 at 15 μM)<br>IC_{50} = 12.8 ± 0.5 μM	[16]
**324**	Inhibitor of the TPA-induced activation of AP-1 at 15 μM<br>IC_{50} = 7.1 ± 0.3 μM	CURCUMIN<br>~55% (Inhibition of the TPA-induced activation of AP-1 at 15 μM)<br>IC_{50} = 12.8 ± 0.5 μM	[16]
**325**	Inhibitor of the TPA-induced activation of AP-1 at 15 μM<br>IC_{50} = 8.2 ± 0.3 μM	CURCUMIN<br>~55% (Inhibition of the TPA-induced activation of AP-1 at 15 μM)<br>IC_{50} = 12.8 ± 0.5 μM	[16]
**326**	Inhibitor of the TPA-induced activation of AP-1 at 15 μM<br>IC_{50} = 4.1 ± 0.3 μM	CURCUMIN<br>~55% (Inhibition of the TPA-induced activation of AP-1 at 15 μM)<br>IC_{50} = 12.8 ± 0.5 μM	[16]
**327**	Inhibitor of the TPA-induced activation of AP-1 at 15 μM<br>IC_{50} = 4.8 ± 0.2 μM	CURCUMIN<br>~55% (Inhibition of the TPA-induced activation of AP-1 at 15 μM)<br>IC_{50} = 12.8 ± 0.5 μM	[16]

**328**	Inhibitor of the TPA-induced activation of AP-1 at 15 μM<br>IC_{50} = 6.0 ± 0.4 μM	CURCUMIN<br>~55% (Inhibition of the TPA-induced activation of AP-1 at 15 μM)<br>IC_{50} = 12.8 ± 0.5 μM	[16]
**329**	Inhibitor of the TPA-induced activation of AP-1 at 15 μM	CURCUMIN<br>~55% (Inhibition of the TPA-induced activation of AP-1 at 15 μM)<br>IC_{50} = 12.8 ± 0.5 μM	[16]
**330**	Inhibitor of the TPA-induced activation of AP-1 at 15 μM	CURCUMIN<br>~55% (Inhibition of the TPA-induced activation of AP-1 at 15 μM)<br>IC_{50} = 12.8 ± 0.5 μM	[16]
**331**	Inhibitor of the TPA-induced activation of AP-1 at 15 μM	CURCUMIN<br>~55% (Inhibition of the TPA-induced activation of AP-1 at 15 μM)<br>IC_{50} = 12.8 ± 0.5 μM	[16]
**332**	Inhibitor of the TPA-induced activation of AP-1 at 15 μM	CURCUMIN<br>~55% (Inhibition of the TPA-induced activation of AP-1 at 15 μM)<br>IC_{50} = 12.8 ± 0.5 μM	[16]
**333**	Inhibitor of the TPA-induced activation of AP-1 at 15 μM	CURCUMIN<br>~55% (Inhibition of the TPA-induced activation of AP-1 at 15 μM)<br>IC_{50} = 12.8 ± 0.5 μM	[16]
**334**	Enhancer of the TPA-induced activation of AP-1 at 15 μM	CURCUMIN<br>~55% (Inhibition of the TPA-induced activation of AP-1 at 15 μM)<br>IC_{50} = 12.8 ± 0.5 μM	[16]
**335**	Enhancer of the TPA-induced activation of AP-1 at 15 μM	CURCUMIN<br>~55% (Inhibition of the TPA-induced activation of AP-1 at 15 μM)<br>IC_{50} = 12.8 ± 0.5 μM	[16]
**336**	Enhancer of the TPA-induced activation of AP-1 at 15 μM	CURCUMIN<br>~55% (Inhibition of the TPA-induced activation of AP-1 at 15 μM)<br>IC_{50} = 12.8 ± 0.5 μM	[16]
**337**	Enhancer of the TPA-induced activation of AP-1 at 15 μM	CURCUMIN<br>~55% (Inhibition of the TPA-induced activation of AP-1 at 15 μM)<br>IC_{50} = 12.8 ± 0.5 μM	[16]
**338**	Enhancer of the TPA-induced activation of AP-1 at 15 μM	CURCUMIN<br>~55% (Inhibition of the TPA-induced activation of AP-1 at 15 μM)<br>IC_{50} = 12.8 ± 0.5 μM	[16]
**339**	Enhancer of the TPA-induced activation of AP-1 at 15 μM	CURCUMIN<br>~55% (Inhibition of the TPA-induced activation of AP-1 at 15 μM)<br>IC_{50} = 12.8 ± 0.5 μM	[16]
**340**	Enhancer of the TPA-induced activation of AP-1 at 15 μM	CURCUMIN<br>~55% (Inhibition of the TPA-induced activation of AP-1 at 15 μM)<br>IC_{50} = 12.8 ± 0.5 μM	[16]

**341**	Enhancer of the TPA-induced activation of AP-1 at 15 μM	CURCUMIN<br>~55% (Inhibition of the TPA-induced activation of AP-1 at 15 μM)<br>IC_{50} = 12.8 ± 0.5 μM	[16]
**342**	Enhancer of the TPA-induced activation of AP-1 at 15 μM	CURCUMIN<br>~55% (Inhibition of the TPA-induced activation of AP-1 at 15 μM)<br>IC_{50} = 12.8 ± 0.5 μM	[16]
**343**	Enhancer of the TPA-induced activation of AP-1 at 15 μM	CURCUMIN<br>~55% (Inhibition of the TPA-induced activation of AP-1 at 15 μM)<br>IC_{50} = 12.8 ± 0.5 μM	[16]
**344**	Enhancer of the TPA-induced activation of AP-1 at 15 μM	CURCUMIN<br>~55% (Inhibition of the TPA-induced activation of AP-1 at 15 μM)<br>IC_{50} = 12.8 ± 0.5 μM	[16]
**345**	Enhancer of the TPA-induced activation of AP-1 at 15 μM	CURCUMIN<br>~55% (Inhibition of the TPA-induced activation of AP-1 at 15 μM)<br>IC_{50} = 12.8 ± 0.5 μM	[16]
**346**	Enhancer of the TPA-induced activation of AP-1 at 15 μM	CURCUMIN<br>~55% (Inhibition of the TPA-induced activation of AP-1 at 15 μM)<br>IC_{50} = 12.8 ± 0.5 μM	[16]
**2**	~105% (relative amount of TNF-α (% of LPS))<br>~140% (relative amount of IL-6 (% of LPS))	CURCUMIN<br>~90% (relative amount of TNF-α (% of LPS))<br>~60% (relative amount of IL-6 (% of LPS))	[17]
**3**	~110% (relative amount of TNF-α (% of LPS))<br>~60% (relative amount of IL-6 (% of LPS))	CURCUMIN<br>~90% (relative amount of TNF-α (% of LPS))<br>~60% (relative amount of IL-6 (% of LPS))	[17]
**6**	~100% (relative amount of TNF-α (% of LPS))<br>~70% (relative amount of IL-6 (% of LPS))	CURCUMIN<br>~90% (relative amount of TNF-α (% of LPS))<br>~60% (relative amount of IL-6 (% of LPS))	[17]
**7**	~70% (relative amount of TNF-α (% of LPS))<br>~80% (relative amount of IL-6 (% of LPS))	CURCUMIN<br>~90% (relative amount of TNF-α (% of LPS))<br>~60% (relative amount of IL-6 (% of LPS))	[17]
**8**	~210% (relative amount of TNF-α (% of LPS))<br>~80% (relative amount of IL-6 (% of LPS))	CURCUMIN<br>~90% (relative amount of TNF-α (% of LPS))<br>~60% (relative amount of IL-6 (% of LPS))	[17]
**9**	~40% (relative amount of TNF-α (% of LPS))<br>~40% (relative amount of IL-6 (% of LPS))	CURCUMIN<br>~90% (relative amount of TNF-α (% of LPS))<br>~60% (relative amount of IL-6 (% of LPS))	[17]
**10**	~70% (relative amount of TNF-α (% of LPS))<br>~70% (relative amount of IL-6 (% of LPS))	CURCUMIN<br>~90% (relative amount of TNF-α (% of LPS))<br>~60% (relative amount of IL-6 (% of LPS))	[17]

**11**	~80% (relative amount of TNF-α (% of LPS))<br>~60% (relative amount of IL-6 (% of LPS))	CURCUMIN<br>~90% (relative amount of TNF-α (% of LPS))<br>~60% (relative amount of IL-6 (% of LPS))	[17]
**12**	~80% (relative amount of TNF-α (% of LPS))<br>~80% (relative amount of IL-6 (% of LPS))	CURCUMIN<br>~90% (relative amount of TNF-α (% of LPS))<br>~60% (relative amount of IL-6 (% of LPS))	[17]
**13**	~90% (relative amount of TNF-α (% of LPS))<br>~105% (relative amount of IL-6 (% of LPS))	CURCUMIN<br>~90% (relative amount of TNF-α (% of LPS))<br>~60% (relative amount of IL-6 (% of LPS))	[17]
**14**	~130% (relative amount of TNF-α (% of LPS))<br>~90% (relative amount of IL-6 (% of LPS))	CURCUMIN<br>~90% (relative amount of TNF-α (% of LPS))<br>~60% (relative amount of IL-6 (% of LPS))	[17]
**17**	~80% (relative amount of TNF-α (% of LPS))<br>~90% (relative amount of IL-6 (% of LPS))	CURCUMIN<br>~90% (relative amount of TNF-α (% of LPS))<br>~60% (relative amount of IL-6 (% of LPS))	[17]
**20**	~60% (relative amount of TNF-α (% of LPS))<br>~70% (relative amount of IL-6 (% of LPS))	CURCUMIN<br>~90% (relative amount of TNF-α (% of LPS))<br>~60% (relative amount of IL-6 (% of LPS))	[17]
**21**	~70% (relative amount of TNF-α (% of LPS))<br>~40% (relative amount of IL-6 (% of LPS))	CURCUMIN<br>~90% (relative amount of TNF-α (% of LPS))<br>~60% (relative amount of IL-6 (% of LPS))	[17]
**22**	~140% (relative amount of TNF-α (% of LPS))<br>~110% (relative amount of IL-6 (% of LPS))	CURCUMIN<br>~90% (relative amount of TNF-α (% of LPS))<br>~60% (relative amount of IL-6 (% of LPS))	[17]
**23**	~60% (relative amount of TNF-α (% of LPS))<br>~60% (relative amount of IL-6 (% of LPS))	CURCUMIN<br>~90% (relative amount of TNF-α (% of LPS))<br>~60% (relative amount of IL-6 (% of LPS))	[17]
**26**	~100% (relative amount of TNF-α (% of LPS))<br>~80% (relative amount of IL-6 (% of LPS))	CURCUMIN<br>~90% (relative amount of TNF-α (% of LPS))<br>~60% (relative amount of IL-6 (% of LPS))	[17]
**27**	~60% (relative amount of TNF-α (% of LPS))<br>~40% (relative amount of IL-6 (% of LPS))	CURCUMIN<br>~90% (relative amount of TNF-α (% of LPS))<br>~60% (relative amount of IL-6 (% of LPS))	[17]
**28**	~90% (relative amount of TNF-α (% of LPS))<br>~80% (relative amount of IL-6 (% of LPS))	CURCUMIN<br>~90% (relative amount of TNF-α (% of LPS))<br>~60% (relative amount of IL-6 (% of LPS))	[17]
**29**	~100% (relative amount of TNF-α (% of LPS))<br>~50% (relative amount of IL-6 (% of LPS))	CURCUMIN<br>~90% (relative amount of TNF-α (% of LPS))<br>~60% (relative amount of IL-6 (% of LPS))	[17]

**31**	~70% (relative amount of TNF-α (% of LPS))<br>~105% (relative amount of IL-6 (% of LPS))	CURCUMIN<br>~90% (relative amount of TNF-α (% of LPS))<br>~60% (relative amount of IL-6 (% of LPS))	[17]
**32**	~60% (relative amount of TNF-α (% of LPS))<br>~70% (relative amount of IL-6 (% of LPS))	CURCUMIN<br>~90% (relative amount of TNF-α (% of LPS))<br>~60% (relative amount of IL-6 (% of LPS))	[17]
**33**	~80% (relative amount of TNF-α (% of LPS))<br>~60% (relative amount of IL-6 (% of LPS))	CURCUMIN<br>~90% (relative amount of TNF-α (% of LPS))<br>~60% (relative amount of IL-6 (% of LPS))	[17]
**34**	~70% (relative amount of TNF-α (% of LPS))<br>~30% (relative amount of IL-6 (% of LPS))	CURCUMIN<br>~90% (relative amount of TNF-α (% of LPS))<br>~60% (relative amount of IL-6 (% of LPS))	[17]
**35**	~40% (relative amount of TNF-α (% of LPS))<br>~55% (relative amount of IL-6 (% of LPS))	CURCUMIN<br>~90% (relative amount of TNF-α (% of LPS))<br>~60% (relative amount of IL-6 (% of LPS))	[17]
**36**	~60% (relative amount of TNF-α (% of LPS))<br>~60% (relative amount of IL-6 (% of LPS))	CURCUMIN<br>~90% (relative amount of TNF-α (% of LPS))<br>~60% (relative amount of IL-6 (% of LPS))	[17]
**164**	~90% (relative amount of TNF-α (% of LPS))<br>~90% (relative amount of IL-6 (% of LPS))	CURCUMIN<br>~90% (relative amount of TNF-α (% of LPS))<br>~60% (relative amount of IL-6 (% of LPS))	[17]
**340**	~60% (relative amount of TNF-α (% of LPS))<br>~40% (relative amount of IL-6 (% of LPS))	CURCUMIN<br>~90% (relative amount of TNF-α (% of LPS))<br>~60% (relative amount of IL-6 (% of LPS))	[17]
**347**	~50% (relative amount of TNF-α (% of LPS))<br>~50% (relative amount of IL-6 (% of LPS))	CURCUMIN<br>~90% (relative amount of TNF-α (% of LPS))<br>~60% (relative amount of IL-6 (% of LPS))	[17]
**348**	~60% (relative amount of TNF-α (% of LPS))<br>~80% (relative amount of IL-6 (% of LPS))	CURCUMIN<br>~90% (relative amount of TNF-α (% of LPS))<br>~60% (relative amount of IL-6 (% of LPS))	[17]
**349**	~145% (relative amount of TNF-α (% of LPS))<br>~110% (relative amount of IL-6 (% of LPS))	CURCUMIN<br>~90% (relative amount of TNF-α (% of LPS))<br>~60% (relative amount of IL-6 (% of LPS))	[17]
**350**	~95% (relative amount of TNF-α (% of LPS))<br>~90% (relative amount of IL-6 (% of LPS))	CURCUMIN<br>~90% (relative amount of TNF-α (% of LPS))<br>~60% (relative amount of IL-6 (% of LPS))	[17]
**351**	~70% (relative amount of TNF-α (% of LPS))<br>~80% (relative amount of IL-6 (% of LPS))	CURCUMIN<br>~90% (relative amount of TNF-α (% of LPS))<br>~60% (relative amount of IL-6 (% of LPS))	[17]

**352**	~130% (relative amount of TNF-α (% of LPS))<br>~90% (relative amount of IL-6 (% of LPS))	CURCUMIN<br>~90% (relative amount of TNF-α (% of LPS))<br>~60% (relative amount of IL-6 (% of LPS))	[17]
**353**	~70% (relative amount of TNF-α (% of LPS))<br>~110% (relative amount of IL-6 (% of LPS))	CURCUMIN<br>~90% (relative amount of TNF-α (% of LPS))<br>~60% (relative amount of IL-6 (% of LPS))	[17]
**3**	~70% (relative amount of TNF-α (% of LPS))<br>~50% (relative amount of IL-6 (% of LPS))	CURCUMIN<br>~80% (relative amount of TNF-α (% of LPS))<br>~60% (relative amount of IL-6 (% of LPS))	[18]
**7**	~170% (relative amount of TNF-α (% of LPS))<br>~10% (relative amount of IL-6 (% of LPS))	CURCUMIN<br>~80% (relative amount of TNF-α (% of LPS))<br>~60% (relative amount of IL-6 (% of LPS))	[18]
**13**	~60% (relative amount of TNF-α (% of LPS))<br>~60% (relative amount of IL-6 (% of LPS))	CURCUMIN<br>~80% (relative amount of TNF-α (% of LPS))<br>~60% (relative amount of IL-6 (% of LPS))	[18]
**17**	~150% (relative amount of TNF-α (% of LPS))<br>~100% (relative amount of IL-6 (% of LPS))	CURCUMIN<br>~80% (relative amount of TNF-α (% of LPS))<br>~60% (relative amount of IL-6 (% of LPS))	[18]
**20**	~50% (relative amount of TNF-α (% of LPS))<br>~50% (relative amount of IL-6 (% of LPS))	CURCUMIN<br>~80% (relative amount of TNF-α (% of LPS))<br>~60% (relative amount of IL-6 (% of LPS))	[18]
**28**	~60% (relative amount of TNF-α (% of LPS))<br>~50% (relative amount of IL-6 (% of LPS))	CURCUMIN<br>~80% (relative amount of TNF-α (% of LPS))<br>~60% (relative amount of IL-6 (% of LPS))	[18]
**31**	~70% (relative amount of TNF-α (% of LPS))<br>~50% (relative amount of IL-6 (% of LPS))	CURCUMIN<br>~80% (relative amount of TNF-α (% of LPS))<br>~60% (relative amount of IL-6 (% of LPS))	[18]
**32**	~60% (relative amount of TNF-α (% of LPS))<br>~50% (relative amount of IL-6 (% of LPS))	CURCUMIN<br>~80% (relative amount of TNF-α (% of LPS))<br>~60% (relative amount of IL-6 (% of LPS))	[18]
**354**	~50% (relative amount of TNF-α (% of LPS))<br>~40% (relative amount of IL-6 (% of LPS))	CURCUMIN<br>~80% (relative amount of TNF-α (% of LPS))<br>~60% (relative amount of IL-6 (% of LPS))	[18]
**8**	~150% (relative amount of TNF-α (% of LPS))<br>~80% (relative amount of IL-6 (% of LPS))	CURCUMIN<br>~85% (relative amount of TNF-α (% of LPS))<br>~70% (relative amount of IL-6 (% of LPS))	[19]
**9**	~40% (relative amount of TNF-α (% of LPS))<br>~35% (relative amount of IL-6 (% of LPS))	CURCUMIN<br>~85% (relative amount of TNF-α (% of LPS))<br>~70% (relative amount of IL-6 (% of LPS))	[19]

**21**	~80% (relative amount of TNF-α (% of LPS))<br>~40% (relative amount of IL-6 (% of LPS))	CURCUMIN<br>~85% (relative amount of TNF-α (% of LPS))<br>~70% (relative amount of IL-6 (% of LPS))	[19]
**22**	~140% (relative amount of TNF-α (% of LPS))<br>~120% (relative amount of IL-6 (% of LPS))	CURCUMIN<br>~85% (relative amount of TNF-α (% of LPS))<br>~70% (relative amount of IL-6 (% of LPS))	[19]
**23**	~45% (relative amount of TNF-α (% of LPS))<br>~70% (relative amount of IL-6 (% of LPS))	CURCUMIN<br>~85% (relative amount of TNF-α (% of LPS))<br>~70% (relative amount of IL-6 (% of LPS))	[19]
**33**	~80% (relative amount of TNF-α (% of LPS))<br>~60% (relative amount of IL-6 (% of LPS))	CURCUMIN<br>~85% (relative amount of TNF-α (% of LPS))<br>~70% (relative amount of IL-6 (% of LPS))	[19]
**34**	~70% (relative amount of TNF-α (% of LPS))<br>~30% (relative amount of IL-6 (% of LPS))	CURCUMIN<br>~85% (relative amount of TNF-α (% of LPS))<br>~70% (relative amount of IL-6 (% of LPS))	[19]
**216**	~10% (relative amount of TNF-α (% of LPS))<br>~95% (relative amount of IL-6 (% of LPS))	CURCUMIN<br>~85% (relative amount of TNF-α (% of LPS))<br>~70% (relative amount of IL-6 (% of LPS))	[19]
**217**	~50% (relative amount of TNF-α (% of LPS))<br>~60% (relative amount of IL-6 (% of LPS))	CURCUMIN<br>~85% (relative amount of TNF-α (% of LPS))<br>~70% (relative amount of IL-6 (% of LPS))	[19]
**341**	~60% (relative amount of TNF-α (% of LPS))<br>~55% (relative amount of IL-6 (% of LPS))	CURCUMIN<br>~85% (relative amount of TNF-α (% of LPS))<br>~70% (relative amount of IL-6 (% of LPS))	[19]
**347**	~80% (relative amount of TNF-α (% of LPS))<br>~115% (relative amount of IL-6 (% of LPS))	CURCUMIN<br>~85% (relative amount of TNF-α (% of LPS))<br>~70% (relative amount of IL-6 (% of LPS))	[19]
**348**	~65% (relative amount of TNF-α (% of LPS))<br>~90% (relative amount of IL-6 (% of LPS))	CURCUMIN<br>~85% (relative amount of TNF-α (% of LPS))<br>~70% (relative amount of IL-6 (% of LPS))	[19]
**352**	~100% (relative amount of TNF-α (% of LPS))<br>~95% (relative amount of IL-6 (% of LPS))	CURCUMIN<br>~85% (relative amount of TNF-α (% of LPS))<br>~70% (relative amount of IL-6 (% of LPS))	[19]
**353**	~80% (relative amount of TNF-α (% of LPS))<br>~105% (relative amount of IL-6 (% of LPS))	CURCUMIN<br>~85% (relative amount of TNF-α (% of LPS))<br>~70% (relative amount of IL-6 (% of LPS))	[19]
**355**	~55% (relative amount of TNF-α (% of LPS))<br>~80% (relative amount of IL-6 (% of LPS))	CURCUMIN<br>~85% (relative amount of TNF-α (% of LPS))<br>~70% (relative amount of IL-6 (% of LPS))	[19]

**356**	~85% (relative amount of TNF-α (% of LPS))<br>~80% (relative amount of IL-6 (% of LPS))	CURCUMIN<br>~85% (relative amount of TNF-α (% of LPS))<br>~70% (relative amount of IL-6 (% of LPS))	[19]
**71**	~65% (relative amount of TNF-α (% of LPS))<br>~70% (relative amount of IL-6 (% of LPS))	CURCUMIN<br>~85% (relative amount of TNF-α (% of LPS))<br>~70% (relative amount of IL-6 (% of LPS))	[19]
**357**	~100% (relative amount of TNF-α (% of LPS))<br>~95% (relative amount of IL-6 (% of LPS))	CURCUMIN<br>~85% (relative amount of TNF-α (% of LPS))<br>~70% (relative amount of IL-6 (% of LPS))	[19]
**358**	~65% (relative amount of TNF-α (% of LPS))<br>~65% (relative amount of IL-6 (% of LPS))	CURCUMIN<br>~85% (relative amount of TNF-α (% of LPS))<br>~70% (relative amount of IL-6 (% of LPS))	[19]
**359**	~55% (relative amount of TNF-α (% of LPS))<br>~65% (relative amount of IL-6 (% of LPS))	CURCUMIN<br>~85% (relative amount of TNF-α (% of LPS))<br>~70% (relative amount of IL-6 (% of LPS))	[19]
**360**	~85% (relative amount of TNF-α (% of LPS))<br>~150% (relative amount of IL-6 (% of LPS))	CURCUMIN<br>~85% (relative amount of TNF-α (% of LPS))<br>~70% (relative amount of IL-6 (% of LPS))	[19]
**361**	~85% (relative amount of TNF-α (% of LPS))<br>~80% (relative amount of IL-6 (% of LPS))	CURCUMIN<br>~85% (relative amount of TNF-α (% of LPS))<br>~70% (relative amount of IL-6 (% of LPS))	[19]
**362**	~90% (relative amount of TNF-α (% of LPS))<br>~100% (relative amount of IL-6 (% of LPS))	CURCUMIN<br>~85% (relative amount of TNF-α (% of LPS))<br>~70% (relative amount of IL-6 (% of LPS))	[19]
**363**	~90% (relative amount of TNF-α (% of LPS))<br>~90% (relative amount of IL-6 (% of LPS))	CURCUMIN<br>~85% (relative amount of TNF-α (% of LPS))<br>~70% (relative amount of IL-6 (% of LPS))	[19]
**364**	~90% (relative amount of TNF-α (% of LPS))<br>~85% (relative amount of IL-6 (% of LPS))	CURCUMIN<br>~85% (relative amount of TNF-α (% of LPS))<br>~70% (relative amount of IL-6 (% of LPS))	[19]
**365**	~55% (relative amount of TNF-α (% of LPS))<br>~55% (relative amount of IL-6 (% of LPS))	CURCUMIN<br>~85% (relative amount of TNF-α (% of LPS))<br>~70% (relative amount of IL-6 (% of LPS))	[19]
**366**	~80% (relative amount of TNF-α (% of LPS))<br>~100% (relative amount of IL-6 (% of LPS))	CURCUMIN<br>~85% (relative amount of TNF-α (% of LPS))<br>~70% (relative amount of IL-6 (% of LPS))	[19]
**367**	~80% (relative amount of TNF-α (% of LPS))<br>~130% (relative amount of IL-6 (% of LPS))	CURCUMIN<br>~85% (relative amount of TNF-α (% of LPS))<br>~70% (relative amount of IL-6 (% of LPS))	[19]

**368**	~35% (relative amount of TNF-α (% of LPS))<br>~15% (relative amount of IL-6 (% of LPS))	CURCUMIN<br>~85% (relative amount of TNF-α (% of LPS))<br>~70% (relative amount of IL-6 (% of LPS))	[19]
**369**	~35% (relative amount of TNF-α (% of LPS))<br>~30% (relative amount of IL-6 (% of LPS))	CURCUMIN<br>~85% (relative amount of TNF-α (% of LPS))<br>~70% (relative amount of IL-6 (% of LPS))	[19]
**370**	~40% (relative amount of TNF-α (% of LPS))<br>~50% (relative amount of IL-6 (% of LPS))	CURCUMIN<br>~85% (relative amount of TNF-α (% of LPS))<br>~70% (relative amount of IL-6 (% of LPS))	[19]
**371**	~70% (relative amount of TNF-α (% of LPS))<br>~65% (relative amount of IL-6 (% of LPS))	CURCUMIN<br>~85% (relative amount of TNF-α (% of LPS))<br>~70% (relative amount of IL-6 (% of LPS))	[19]
**372**	~80% (relative amount of TNF-α (% of LPS))<br>~30% (relative amount of IL-6 (% of LPS))	CURCUMIN<br>~85% (relative amount of TNF-α (% of LPS))<br>~70% (relative amount of IL-6 (% of LPS))	[19]
**373**	~100% (relative amount of TNF-α (% of LPS))<br>~100% (relative amount of IL-6 (% of LPS))	CURCUMIN<br>~85% (relative amount of TNF-α (% of LPS))<br>~70% (relative amount of IL-6 (% of LPS))	[19]
**374**	~50% (relative amount of TNF-α (% of LPS))<br>~45% (relative amount of IL-6 (% of LPS))	CURCUMIN<br>~85% (relative amount of TNF-α (% of LPS))<br>~70% (relative amount of IL-6 (% of LPS))	[19]
**375**	~75% (relative amount of TNF-α (% of LPS))<br>~115% (relative amount of IL-6 (% of LPS))	CURCUMIN<br>~85% (relative amount of TNF-α (% of LPS))<br>~70% (relative amount of IL-6 (% of LPS))	[19]
**376**	~110% (relative amount of TNF-α (% of LPS))<br>~75% (relative amount of IL-6 (% of LPS))	CURCUMIN<br>~85% (relative amount of TNF-α (% of LPS))<br>~70% (relative amount of IL-6 (% of LPS))	[19]
**377**	~130% (relative amount of TNF-α (% of LPS))<br>~80% (relative amount of IL-6 (% of LPS))	CURCUMIN<br>~85% (relative amount of TNF-α (% of LPS))<br>~70% (relative amount of IL-6 (% of LPS))	[19]
**378**	~80% (relative amount of TNF-α (% of LPS))<br>~50% (relative amount of IL-6 (% of LPS))	CURCUMIN<br>~85% (relative amount of TNF-α (% of LPS))<br>~70% (relative amount of IL-6 (% of LPS))	[19]
**379**	~55% (relative amount of TNF-α (% of LPS))<br>~50% (relative amount of IL-6 (% of LPS))	CURCUMIN<br>~85% (relative amount of TNF-α (% of LPS))<br>~70% (relative amount of IL-6 (% of LPS))	[19]
**380**	~85% (relative amount of TNF-α (% of LPS))<br>~90% (relative amount of IL-6 (% of LPS))	CURCUMIN<br>~85% (relative amount of TNF-α (% of LPS))<br>~70% (relative amount of IL-6 (% of LPS))	[19]

**381**	~70% (relative amount of TNF-α (% of LPS))<br>~80% (relative amount of IL-6 (% of LPS))	CURCUMIN<br>~85% (relative amount of TNF-α (% of LPS))<br>~70% (relative amount of IL-6 (% of LPS))	[19]
**382**	~70% (relative amount of TNF-α (% of LPS))<br>~70% (relative amount of IL-6 (% of LPS))	CURCUMIN<br>~85% (relative amount of TNF-α (% of LPS))<br>~70% (relative amount of IL-6 (% of LPS))	[19]
**329**	~50 (relative amount of TNF-α (% of LPS))<br>~60 (relative amount of IL-6 (% of LPS))	CURCUMIN<br>~50 (relative amount of TNF-α (% of LPS))<br>~30 (relative amount of IL-6 (% of LPS))	[20]
**383**	~90 (relative amount of TNF-α (% of LPS))<br>~90 (relative amount of IL-6 (% of LPS))	CURCUMIN<br>~50 (relative amount of TNF-α (% of LPS))<br>~30 (relative amount of IL-6 (% of LPS))	[20]
**384**	~60 (relative amount of TNF-α (% of LPS))<br>~50 (relative amount of IL-6 (% of LPS))	CURCUMIN<br>~50 (relative amount of TNF-α (% of LPS))<br>~30 (relative amount of IL-6 (% of LPS))	[20]
**385**	~40 (relative amount of TNF-α (% of LPS))<br>~40 (relative amount of IL-6 (% of LPS))	CURCUMIN<br>~50 (relative amount of TNF-α (% of LPS))<br>~30 (relative amount of IL-6 (% of LPS))	[20]
**386**	~100 (relative amount of TNF-α (% of LPS))<br>~110 (relative amount of IL-6 (% of LPS))	CURCUMIN<br>~50 (relative amount of TNF-α (% of LPS))<br>~30 (relative amount of IL-6 (% of LPS))	[20]
**387**	~60 (relative amount of TNF-α (% of LPS))<br>~90 (relative amount of IL-6 (% of LPS))	CURCUMIN<br>~50 (relative amount of TNF-α (% of LPS))<br>~30 (relative amount of IL-6 (% of LPS))	[20]
**388**	~40 (relative amount of TNF-α (% of LPS))<br>~40 (relative amount of IL-6 (% of LPS))	CURCUMIN<br>~50 (relative amount of TNF-α (% of LPS))<br>~30 (relative amount of IL-6 (% of LPS))	[20]
**389**	~60 (relative amount of TNF-α (% of LPS))<br>~80 (relative amount of IL-6 (% of LPS))	CURCUMIN<br>~50 (relative amount of TNF-α (% of LPS))<br>~30 (relative amount of IL-6 (% of LPS))	[20]
**390**	~60 (relative amount of TNF-α (% of LPS))<br>~50 (relative amount of IL-6 (% of LPS))	CURCUMIN<br>~50 (relative amount of TNF-α (% of LPS))<br>~30 (relative amount of IL-6 (% of LPS))	[20]
**391**	~90 (relative amount of TNF-α (% of LPS))<br>~100 (relative amount of IL-6 (% of LPS))	CURCUMIN<br>~50 (relative amount of TNF-α (% of LPS))<br>~30 (relative amount of IL-6 (% of LPS))	[20]
**392**	~100 (relative amount of TNF-α (% of LPS))<br>~100 (relative amount of IL-6 (% of LPS))	CURCUMIN<br>~50 (relative amount of TNF-α (% of LPS))<br>~30 (relative amount of IL-6 (% of LPS))	[20]

**393**	~50 (relative amount of TNF-α (% of LPS))<br>~100 (relative amount of IL-6 (% of LPS))	CURCUMIN<br>~50 (relative amount of TNF-α (% of LPS))<br>~30 (relative amount of IL-6 (% of LPS))	[20]
**394**	~120 (relative amount of TNF-α (% of LPS))<br>~110 (relative amount of IL-6 (% of LPS))	CURCUMIN<br>~50 (relative amount of TNF-α (% of LPS))<br>~30 (relative amount of IL-6 (% of LPS))	[20]
**395**	~50 (relative amount of TNF-α (% of LPS))<br>~80 (relative amount of IL-6 (% of LPS))	CURCUMIN<br>~50 (relative amount of TNF-α (% of LPS))<br>~30 (relative amount of IL-6 (% of LPS))	[20]
**396**	~70 (relative amount of TNF-α (% of LPS))<br>~100 (relative amount of IL-6 (% of LPS))	CURCUMIN<br>~50 (relative amount of TNF-α (% of LPS))<br>~30 (relative amount of IL-6 (% of LPS))	[20]
**397**	~60 (relative amount of TNF-α (% of LPS))<br>~100 (relative amount of IL-6 (% of LPS))	CURCUMIN<br>~50 (relative amount of TNF-α (% of LPS))<br>~30 (relative amount of IL-6 (% of LPS))	[20]
**398**	~80 (relative amount of TNF-α (% of LPS))<br>~120 (relative amount of IL-6 (% of LPS))	CURCUMIN<br>~50 (relative amount of TNF-α (% of LPS))<br>~30 (relative amount of IL-6 (% of LPS))	[20]
**399**	~100 (relative amount of TNF-α (% of LPS))<br>~100 (relative amount of IL-6 (% of LPS))	CURCUMIN<br>~50 (relative amount of TNF-α (% of LPS))<br>~30 (relative amount of IL-6 (% of LPS))	[20]
**400**	~50 (relative amount of TNF-α (% of LPS))<br>~50 (relative amount of IL-6 (% of LPS))	CURCUMIN<br>~50 (relative amount of TNF-α (% of LPS))<br>~30 (relative amount of IL-6 (% of LPS))	[20]
**401**	~150 (relative amount of TNF-α (% of LPS))<br>~100 (relative amount of IL-6 (% of LPS))	CURCUMIN<br>~50 (relative amount of TNF-α (% of LPS))<br>~30 (relative amount of IL-6 (% of LPS))	[20]
**402**	~140 (relative amount of TNF-α (% of LPS))<br>~110 (relative amount of IL-6 (% of LPS))	CURCUMIN<br>~50 (relative amount of TNF-α (% of LPS))<br>~30 (relative amount of IL-6 (% of LPS))	[20]
**403**	~60 (relative amount of TNF-α (% of LPS))<br>~100 (relative amount of IL-6 (% of LPS))	CURCUMIN<br>~50 (relative amount of TNF-α (% of LPS))<br>~30 (relative amount of IL-6 (% of LPS))	[20]
**404**	~5 (relative amount of TNF-α (% of LPS))<br>~4 (relative amount of IL-6 (% of LPS))	CURCUMIN<br>~50 (relative amount of TNF-α (% of LPS))<br>~30 (relative amount of IL-6 (% of LPS))	[20]
**405**	~100 (relative amount of TNF-α (% of LPS))<br>~60 (relative amount of IL-6 (% of LPS))	CURCUMIN<br>~80 (relative amount of TNF-α (% of LPS))<br>~20 (relative amount of IL-6 (% of LPS))	[21]

**406**	~100 (relative amount of TNF-α (% of LPS))<br>~70 (relative amount of IL-6 (% of LPS))	CURCUMIN<br>~80 (relative amount of TNF-α (% of LPS))<br>~20 (relative amount of IL-6 (% of LPS))	[21]
**407**	~50 (relative amount of TNF-α (% of LPS))<br>~5 (relative amount of IL-6 (% of LPS))	CURCUMIN<br>~80 (relative amount of TNF-α (% of LPS))<br>~20 (relative amount of IL-6 (% of LPS))	[21]
**408**	~100 (relative amount of TNF-α (% of LPS))<br>~60 (relative amount of IL-6 (% of LPS))	CURCUMIN<br>~80 (relative amount of TNF-α (% of LPS))<br>~20 (relative amount of IL-6 (% of LPS))	[21]
**409**	~100 (relative amount of TNF-α (% of LPS))<br>~100 (relative amount of IL-6 (% of LPS))	CURCUMIN<br>~80 (relative amount of TNF-α (% of LPS))<br>~20 (relative amount of IL-6 (% of LPS))	[21]
**410**	~105 (relative amount of TNF-α (% of LPS))<br>~50 (relative amount of IL-6 (% of LPS))	CURCUMIN<br>~80 (relative amount of TNF-α (% of LPS))<br>~20 (relative amount of IL-6 (% of LPS))	[21]
**411**	~100 (relative amount of TNF-α (% of LPS))<br>~80 (relative amount of IL-6 (% of LPS))	CURCUMIN<br>~80 (relative amount of TNF-α (% of LPS))<br>~20 (relative amount of IL-6 (% of LPS))	[21]
**412**	~100 (relative amount of TNF-α (% of LPS))<br>~80 (relative amount of IL-6 (% of LPS))	CURCUMIN<br>~80 (relative amount of TNF-α (% of LPS))<br>~20 (relative amount of IL-6 (% of LPS))	[21]
**413**	~90 (relative amount of TNF-α (% of LPS))<br>~80 (relative amount of IL-6 (% of LPS))	CURCUMIN<br>~80 (relative amount of TNF-α (% of LPS))<br>~20 (relative amount of IL-6 (% of LPS))	[21]
**414**	~95 (relative amount of TNF-α (% of LPS))<br>~95 (relative amount of IL-6 (% of LPS))	CURCUMIN<br>~80 (relative amount of TNF-α (% of LPS))<br>~20 (relative amount of IL-6 (% of LPS))	[21]
**415**	~80 (relative amount of TNF-α (% of LPS))<br>~70 (relative amount of IL-6 (% of LPS))	CURCUMIN<br>~80 (relative amount of TNF-α (% of LPS))<br>~20 (relative amount of IL-6 (% of LPS))	[21]
**416**	~105 (relative amount of TNF-α (% of LPS))<br>~55 (relative amount of IL-6 (% of LPS))	CURCUMIN<br>~80 (relative amount of TNF-α (% of LPS))<br>~20 (relative amount of IL-6 (% of LPS))	[21]
**417**	~95 (relative amount of TNF-α (% of LPS))<br>~5 (relative amount of IL-6 (% of LPS))	CURCUMIN<br>~80 (relative amount of TNF-α (% of LPS))<br>~20 (relative amount of IL-6 (% of LPS))	[21]
**418**	~60 (relative amount of TNF-α (% of LPS))<br>~20 (relative amount of IL-6 (% of LPS))	CURCUMIN<br>~80 (relative amount of TNF-α (% of LPS))<br>~20 (relative amount of IL-6 (% of LPS))	[21]

**419**	~110 (relative amount of TNF-α (% of LPS))<br>~30 (relative amount of IL-6 (% of LPS))	CURCUMIN<br>~80 (relative amount of TNF-α (% of LPS))<br>~20 (relative amount of IL-6 (% of LPS))	[21]
**420**	~100 (relative amount of TNF-α (% of LPS))<br>~80 (relative amount of IL-6 (% of LPS))	CURCUMIN<br>~80 (relative amount of TNF-α (% of LPS))<br>~20 (relative amount of IL-6 (% of LPS))	[21]
**421**	~100 (relative amount of TNF-α (% of LPS))<br>~40 (relative amount of IL-6 (% of LPS))	CURCUMIN<br>~80 (relative amount of TNF-α (% of LPS))<br>~20 (relative amount of IL-6 (% of LPS))	[21]
**422**	~90 (relative amount of TNF-α (% of LPS))<br>~45 (relative amount of IL-6 (% of LPS))	CURCUMIN<br>~80 (relative amount of TNF-α (% of LPS))<br>~20 (relative amount of IL-6 (% of LPS))	[21]
**423**	~110 (relative amount of TNF-α (% of LPS))<br>~60 (relative amount of IL-6 (% of LPS))	CURCUMIN<br>~80 (relative amount of TNF-α (% of LPS))<br>~20 (relative amount of IL-6 (% of LPS))	[21]
**424**	~100 (relative amount of TNF-α (% of LPS))<br>~70 (relative amount of IL-6 (% of LPS))	CURCUMIN<br>~80 (relative amount of TNF-α (% of LPS))<br>~20 (relative amount of IL-6 (% of LPS))	[21]
**425**	~100 (relative amount of TNF-α (% of LPS))<br>~10 (relative amount of IL-6 (% of LPS))	CURCUMIN<br>~80 (relative amount of TNF-α (% of LPS))<br>~20 (relative amount of IL-6 (% of LPS))	[21]
**426**	~105 (relative amount of TNF-α (% of LPS))<br>~40 (relative amount of IL-6 (% of LPS))	CURCUMIN<br>~80 (relative amount of TNF-α (% of LPS))<br>~20 (relative amount of IL-6 (% of LPS))	[21]
**427**	~105 (relative amount of TNF-α (% of LPS))<br>~45 (relative amount of IL-6 (% of LPS))	CURCUMIN<br>~80 (relative amount of TNF-α (% of LPS))<br>~20 (relative amount of IL-6 (% of LPS))	[21]
**428**	~105 (relative amount of TNF-α (% of LPS))<br>~60 (relative amount of IL-6 (% of LPS))	CURCUMIN<br>~80 (relative amount of TNF-α (% of LPS))<br>~20 (relative amount of IL-6 (% of LPS))	[21]
**429**	~100 (relative amount of TNF-α (% of LPS))<br>~40 (relative amount of IL-6 (% of LPS))	CURCUMIN<br>~80 (relative amount of TNF-α (% of LPS))<br>~20 (relative amount of IL-6 (% of LPS))	[21]
**430**	~95 (relative amount of TNF-α (% of LPS))<br>~45 (relative amount of IL-6 (% of LPS))	CURCUMIN<br>~80 (relative amount of TNF-α (% of LPS))<br>~20 (relative amount of IL-6 (% of LPS))	[21]
**431**	~95 (relative amount of TNF-α (% of LPS))<br>~60 (relative amount of IL-6 (% of LPS))	CURCUMIN<br>~80 (relative amount of TNF-α (% of LPS))<br>~20 (relative amount of IL-6 (% of LPS))	[21]

**432**	~95 (relative amount of TNF-α (% of LPS))<br>~45 (relative amount of IL-6 (% of LPS))	CURCUMIN<br>~80 (relative amount of TNF-α (% of LPS))<br>~20 (relative amount of IL-6 (% of LPS))	[21]
**433**	~30 (relative amount of TNF-α (% of LPS))<br>~10 (relative amount of IL-6 (% of LPS))	CURCUMIN<br>~80 (relative amount of TNF-α (% of LPS))<br>~20 (relative amount of IL-6 (% of LPS))	[21]
**434**	~25 (relative amount of TNF-α (% of LPS))<br>~10 (relative amount of IL-6 (% of LPS))	CURCUMIN<br>~80 (relative amount of TNF-α (% of LPS))<br>~20 (relative amount of IL-6 (% of LPS))	[21]
**435**	~90 (relative amount of TNF-α (% of LPS))<br>~40 (relative amount of IL-6 (% of LPS))	CURCUMIN<br>~80 (relative amount of TNF-α (% of LPS))<br>~20 (relative amount of IL-6 (% of LPS))	[21]
**436**	~90 (relative amount of TNF-α (% of LPS))<br>~40 (relative amount of IL-6 (% of LPS))	CURCUMIN<br>~80 (relative amount of TNF-α (% of LPS))<br>~20 (relative amount of IL-6 (% of LPS))	[21]
**437**	~50 (relative amount of TNF-α (% of LPS))<br>~5 (relative amount of IL-6 (% of LPS))	CURCUMIN<br>~80 (relative amount of TNF-α (% of LPS))<br>~20 (relative amount of IL-6 (% of LPS))	[21]
**438**	~5 (relative amount of TNF-α (% of LPS))<br>~5 (relative amount of IL-6 (% of LPS))	CURCUMIN<br>~90 (relative amount of TNF-α (% of LPS))<br>~45 (relative amount of IL-6 (% of LPS))	[22]
**439**	~60 (relative amount of TNF-α (% of LPS))<br>~20(relative amount of IL-6 (% of LPS))	CURCUMIN<br>~90 (relative amount of TNF-α (% of LPS))<br>~45 (relative amount of IL-6 (% of LPS))	[22]
**440**	~10 (relative amount of TNF-α (% of LPS))<br>~10 (relative amount of IL-6 (% of LPS))	CURCUMIN<br>~90 (relative amount of TNF-α (% of LPS))<br>~45 (relative amount of IL-6 (% of LPS))	[22]
**441**	~40 (relative amount of TNF-α (% of LPS))<br>~10 (relative amount of IL-6 (% of LPS))	CURCUMIN<br>~90 (relative amount of TNF-α (% of LPS))<br>~45 (relative amount of IL-6 (% of LPS))	[22]
**442**	~70 (relative amount of TNF-α (% of LPS))<br>~20 (relative amount of IL-6 (% of LPS))	CURCUMIN<br>~90 (relative amount of TNF-α (% of LPS))<br>~45 (relative amount of IL-6 (% of LPS))	[22]
**443**	~60 (relative amount of TNF-α (% of LPS))<br>~40 (relative amount of IL-6 (% of LPS))	CURCUMIN<br>~90 (relative amount of TNF-α (% of LPS))<br>~45 (relative amount of IL-6 (% of LPS))	[22]
**444**	~60 (relative amount of TNF-α (% of LPS))<br>~30 (relative amount of IL-6 (% of LPS))	CURCUMIN<br>~90 (relative amount of TNF-α (% of LPS))<br>~45 (relative amount of IL-6 (% of LPS))	[22]

**445**	~60 (relative amount of TNF-α (% of LPS))<br>~10 (relative amount of IL-6 (% of LPS))	CURCUMIN<br>~90 (relative amount of TNF-α (% of LPS))<br>~45 (relative amount of IL-6 (% of LPS))	[22]
**446**	~70 (relative amount of TNF-α (% of LPS))<br>~20 (relative amount of IL-6 (% of LPS))	CURCUMIN<br>~90 (relative amount of TNF-α (% of LPS))<br>~45 (relative amount of IL-6 (% of LPS))	[22]
**447**	~30 (relative amount of TNF-α (% of LPS))<br>~20 (relative amount of IL-6 (% of LPS))	CURCUMIN<br>~90 (relative amount of TNF-α (% of LPS))<br>~45 (relative amount of IL-6 (% of LPS))	[22]
**448**	~70 (relative amount of TNF-α (% of LPS))<br>~30 (relative amount of IL-6 (% of LPS))	CURCUMIN<br>~90 (relative amount of TNF-α (% of LPS))<br>~45 (relative amount of IL-6 (% of LPS))	[22]
**449**	~10 (relative amount of TNF-α (% of LPS))<br>~10 (relative amount of IL-6 (% of LPS))	CURCUMIN<br>~90 (relative amount of TNF-α (% of LPS))<br>~45 (relative amount of IL-6 (% of LPS))	[22]
**450**	~20 (relative amount of TNF-α (% of LPS))<br>~20 (relative amount of IL-6 (% of LPS))	CURCUMIN<br>~90 (relative amount of TNF-α (% of LPS))<br>~45 (relative amount of IL-6 (% of LPS))	[22]
**451**	~50 (relative amount of TNF-α (% of LPS))<br>~70 (relative amount of IL-6 (% of LPS))	CURCUMIN<br>~90 (relative amount of TNF-α (% of LPS))<br>~45 (relative amount of IL-6 (% of LPS))	[22]
**452**	~50 (relative amount of TNF-α (% of LPS))<br>~120 (relative amount of IL-6 (% of LPS))	CURCUMIN<br>~90 (relative amount of TNF-α (% of LPS))<br>~45 (relative amount of IL-6 (% of LPS))	[22]
**453**	~80 (relative amount of TNF-α (% of LPS))<br>~80 (relative amount of IL-6 (% of LPS))	CURCUMIN<br>~90 (relative amount of TNF-α (% of LPS))<br>~45 (relative amount of IL-6 (% of LPS))	[22]
**454**	~70 (relative amount of TNF-α (% of LPS))<br>~50 (relative amount of IL-6 (% of LPS))	CURCUMIN<br>~90 (relative amount of TNF-α (% of LPS))<br>~45 (relative amount of IL-6 (% of LPS))	[22]
**455**	~90 (relative amount of TNF-α (% of LPS))<br>~80 (relative amount of IL-6 (% of LPS))	CURCUMIN<br>~90 (relative amount of TNF-α (% of LPS))<br>~45 (relative amount of IL-6 (% of LPS))	[22]
**456**	~80 (relative amount of TNF-α (% of LPS))<br>~80 (relative amount of IL-6 (% of LPS))	CURCUMIN<br>~90 (relative amount of TNF-α (% of LPS))<br>~45 (relative amount of IL-6 (% of LPS))	[22]
**457**	~50 (relative amount of TNF-α (% of LPS))<br>~80 (relative amount of IL-6 (% of LPS))	CURCUMIN<br>~90 (relative amount of TNF-α (% of LPS))<br>~45 (relative amount of IL-6 (% of LPS))	[22]

**458**	~70 (relative amount of TNF-α (% of LPS))<br>~70 (relative amount of IL-6 (% of LPS))	CURCUMIN<br>~90 (relative amount of TNF-α (% of LPS))<br>~45 (relative amount of IL-6 (% of LPS))	[22]
**459**	~70 (relative amount of TNF-α (% of LPS))<br>~50 (relative amount of IL-6 (% of LPS))	CURCUMIN<br>~90 (relative amount of TNF-α (% of LPS))<br>~45 (relative amount of IL-6 (% of LPS))	[22]
**460**	~70 (relative amount of TNF-α (% of LPS))<br>~70 (relative amount of IL-6 (% of LPS))	CURCUMIN<br>~90 (relative amount of TNF-α (% of LPS))<br>~45 (relative amount of IL-6 (% of LPS))	[22]
**461**	~80 (relative amount of TNF-α (% of LPS))<br>~80 (relative amount of IL-6 (% of LPS))	CURCUMIN<br>~90 (relative amount of TNF-α (% of LPS))<br>~45 (relative amount of IL-6 (% of LPS))	[22]
**462**	~50 (relative amount of TNF-α (% of LPS))<br>~70 (relative amount of IL-6 (% of LPS))	CURCUMIN<br>~90 (relative amount of TNF-α (% of LPS))<br>~45 (relative amount of IL-6 (% of LPS))	[22]
**463**	~50 (relative amount of TNF-α (% of LPS))<br>~80 (relative amount of IL-6 (% of LPS))	CURCUMIN<br>~90 (relative amount of TNF-α (% of LPS))<br>~45 (relative amount of IL-6 (% of LPS))	[22]
**464**	~110 (relative amount of TNF-α (% of LPS))<br>~70 (relative amount of IL-6 (% of LPS))	CURCUMIN<br>~70 (relative amount of TNF-α (% of LPS))<br>~60 (relative amount of IL-6 (% of LPS))	[23]
**465**	~10 (relative amount of TNF-α (% of LPS))<br>~100 (relative amount of IL-6 (% of LPS))	CURCUMIN<br>~70 (relative amount of TNF-α (% of LPS))<br>~60 (relative amount of IL-6 (% of LPS))	[23]
**466**	~100 (relative amount of TNF-α (% of LPS))<br>~80 (relative amount of IL-6 (% of LPS))	CURCUMIN<br>~70 (relative amount of TNF-α (% of LPS))<br>~60 (relative amount of IL-6 (% of LPS))	[23]
**467**	~105 (relative amount of TNF-α (% of LPS))<br>~80 (relative amount of IL-6 (% of LPS))	CURCUMIN<br>~70 (relative amount of TNF-α (% of LPS))<br>~60 (relative amount of IL-6 (% of LPS))	[23]
**468**	~70 (relative amount of TNF-α (% of LPS))<br>~60 (relative amount of IL-6 (% of LPS))	CURCUMIN<br>~70 (relative amount of TNF-α (% of LPS))<br>~60 (relative amount of IL-6 (% of LPS))	[23]
**469**	~105 (relative amount of TNF-α (% of LPS))<br>~70 (relative amount of IL-6 (% of LPS))	CURCUMIN<br>~70 (relative amount of TNF-α (% of LPS))<br>~60 (relative amount of IL-6 (% of LPS))	[23]
**470**	~80 (relative amount of TNF-α (% of LPS))<br>~70 (relative amount of IL-6 (% of LPS))	CURCUMIN<br>~70 (relative amount of TNF-α (% of LPS))<br>~60 (relative amount of IL-6 (% of LPS))	[23]

**471**	~80 (relative amount of TNF-α (% of LPS))<br>~55 (relative amount of IL-6 (% of LPS))	CURCUMIN<br>~70 (relative amount of TNF-α (% of LPS))<br>~60 (relative amount of IL-6 (% of LPS))	[23]
**472**	~95 (relative amount of TNF-α (% of LPS))<br>~70 (relative amount of IL-6 (% of LPS))	CURCUMIN<br>~70 (relative amount of TNF-α (% of LPS))<br>~60 (relative amount of IL-6 (% of LPS))	[23]
**473**	~95 (relative amount of TNF-α (% of LPS))<br>~80 (relative amount of IL-6 (% of LPS))	CURCUMIN<br>~70 (relative amount of TNF-α (% of LPS))<br>~60 (relative amount of IL-6 (% of LPS))	[23]
**474**	~70 (relative amount of TNF-α (% of LPS))<br>~70 (relative amount of IL-6 (% of LPS))	CURCUMIN<br>~70 (relative amount of TNF-α (% of LPS))<br>~60 (relative amount of IL-6 (% of LPS))	[23]
**475**	~80 (relative amount of TNF-α (% of LPS))<br>~40 (relative amount of IL-6 (% of LPS))	CURCUMIN<br>~70 (relative amount of TNF-α (% of LPS))<br>~60 (relative amount of IL-6 (% of LPS))	[23]
**476**	~80 (relative amount of TNF-α (% of LPS))<br>~55 (relative amount of IL-6 (% of LPS))	CURCUMIN<br>~70 (relative amount of TNF-α (% of LPS))<br>~60 (relative amount of IL-6 (% of LPS))	[23]
**477**	~90 (relative amount of TNF-α (% of LPS))<br>~60 (relative amount of IL-6 (% of LPS))	CURCUMIN<br>~70 (relative amount of TNF-α (% of LPS))<br>~60 (relative amount of IL-6 (% of LPS))	[23]
**478**	~150 (relative amount of TNF-α (% of LPS))<br>~110 (relative amount of IL-6 (% of LPS))	CURCUMIN<br>~70 (relative amount of TNF-α (% of LPS))<br>~60 (relative amount of IL-6 (% of LPS))	[23]
**479**	~95 (relative amount of TNF-α_% of LS)<br>~90 (relative amount of IL-6 (% of LPS))	CURCUMIN<br>~70 (relative amount of TNF-α (% of LPS))<br>~60 (relative amount of IL-6 (% of LPS))	[23]
**480**	~95 (relative amount of TNF-α (% of LPS))<br>~80 (relative amount of IL-6 (% of LPS))	CURCUMIN<br>~70 (relative amount of TNF-α (% of LPS))<br>~60 (relative amount of IL-6 (% of LPS))	[23]
**481**	~95 (relative amount of TNF-α (% of LPS))<br>~90 (relative amount of IL-6 (% of LPS))	CURCUMIN<br>~70 (relative amount of TNF-α (% of LPS))<br>~60 (relative amount of IL-6 (% of LPS))	[23]
**482**	~105 (relative amount of TNF-α (% of LPS))<br>~80 (relative amount of IL-6 _% of LS)	CURCUMIN<br>~70 (relative amount of TNF-α (% of LPS))<br>~60 (relative amount of IL-6 (% of LPS))	[23]
**483**	~95 (relative amount of TNF-α (% of LPS))<br>~80 (relative amount of IL-6 (% of LPS))	CURCUMIN<br>~70 (relative amount of TNF-α (% of LPS))<br>~60 (relative amount of IL-6 (% of LPS))	[23]

**484**	~95 (relative amount of TNF-α (% of LPS))<br>~90 (relative amount of IL-6 (% of LPS))	CURCUMIN<br>~70 (relative amount of TNF-α (% of LPS))<br>~60 (relative amount of IL-6 (% of LPS))	[23]
**485**	~70 (relative amount of TNF-α (% of LPS))<br>~70 (relative amount of IL-6 (% of LPS))	CURCUMIN<br>~70 (relative amount of TNF-α (% of LPS))<br>~60 (relative amount of IL-6 (% of LPS))	[23]
**486**	~80 (relative amount of TNF-α (% of LPS))<br>~70 (relative amount of IL-6 (% of LPS))	CURCUMIN<br>~70 (relative amount of TNF-α (% of LPS))<br>~60 (relative amount of IL-6 (% of LPS))	[23]
**487**	~95 (relative amount of TNF-α (% of LPS))<br>~90 (relative amount of IL-6 (% of LPS))	CURCUMIN<br>~70 (relative amount of TNF-α (% of LPS))<br>~60 (relative amount of IL-6 (% of LPS))	[23]
**488**	~95 (relative amount of TNF-α (% of LPS))<br>~80 (relative amount of IL-6 (% of LPS))	CURCUMIN<br>~70 (relative amount of TNF-α (% of LPS))<br>~60 (relative amount of IL-6 (% of LPS))	[23]
**489**	~95 (relative amount of TNF-α (% of LPS))<br>~80 (relative amount of IL-6 (% of LPS))	CURCUMIN<br>~70 (relative amount of TNF-α (% of LPS))<br>~60 (relative amount of IL-6 (% of LPS))	[23]
**490**	~100 (relative amount of TNF-α (% of LPS))<br>~70 (relative amount of IL-6 (% of LPS))	CURCUMIN<br>~70 (relative amount of TNF-α (% of LPS))<br>~60 (relative amount of IL-6 (% of LPS))	[23]
**491**	~40 (relative amount of TNF-α (% of LPS))<br>~55 (relative amount of IL-6 (% of LPS))	CURCUMIN<br>~70 (relative amount of TNF-α (% of LPS))<br>~60 (relative amount of IL-6 (% of LPS))	[23]
**492**	~40 (relative amount of TNF-α (% of LPS))<br>~10 (relative amount of IL-6 (% of LPS))	CURCUMIN<br>~70 (relative amount of TNF-α (% of LPS))<br>~60 (relative amount of IL-6 (% of LPS))	[23]
**493**	~20 (relative amount of TNF-α (% of LPS))<br>~10 (relative amount of IL-6 (% of LPS))	CURCUMIN<br>~70 (relative amount of TNF-α (% of LPS))<br>~60 (relative amount of IL-6 (% of LPS))	[23]
**494**	~40 (relative amount of TNF-α (% of LPS))<br>~5 (relative amount of IL-6 (% of LPS))	CURCUMIN<br>~70 (relative amount of TNF-α (% of LPS))<br>~60 (relative amount of IL-6 (% of LPS))	[23]
**495**	~20 (relative amount of TNF-α (% of LPS))<br>~5 (relative amount of IL-6 (% of LPS))	CURCUMIN<br>~70 (relative amount of TNF-α (% of LPS))<br>~60 (relative amount of IL-6 (% of LPS))	[23]
**496**	~40 (relative amount of TNF-α (% of LPS))<br>~10 (relative amount of IL-6 (% of LPS))	CURCUMIN<br>~70 (relative amount of TNF-α (% of LPS))<br>~60 (relative amount of IL-6 (% of LPS))	[23]

**497**	~30 (relative amount of TNF-α (% of LPS))<br>~2 (relative amount of IL-6 (% of LPS))	CURCUMIN<br>~70 (relative amount of TNF-α (% of LPS))<br>~60 (relative amount of IL-6 (% of LPS))	[23]
**498**	Inhibition = 42.46% (IL-6)<br>Inhibition = 27.47% (TNF-α)	CURCUMIN<br>Inhibition = 50.64% (IL-6)<br>Inhibition = 18.48% (TNF-α)	[24]
**499**	Inhibition = 44.67% (IL-6)<br>Inhibition = 23.35% (TNF-α)	CURCUMIN<br>Inhibition = 50.64% (IL-6)<br>Inhibition = 18.48% (TNF-α)	[24]
**500**	Inhibition = 27.83% (IL-6)<br>Inhibition = 9.91% (TNF-α)	CURCUMIN<br>Inhibition = 50.64% (IL-6)<br>Inhibition = 18.48% (TNF-α)	[24]
**501**	Inhibition = 53.28% (IL-6)<br>Inhibition = 38.81% (TNF-α)	CURCUMIN<br>Inhibition = 50.64% (IL-6)<br>Inhibition = 18.48% (TNF-α)	[24]
**502**	Inhibition = -8.79% (IL-6)<br>Inhibition = 47.17% (TNF-α)	CURCUMIN<br>Inhibition = 50.64% (IL-6)<br>Inhibition = 18.48% (TNF-α)	[24]
**503**	Inhibition = 54.53% (IL-6)<br>Inhibition = 91.20% (TNF-α)	CURCUMIN<br>Inhibition = 50.64% (IL-6)<br>Inhibition = 18.48% (TNF-α)	[24]
**504**	Inhibition = -5.86% (IL-6)<br>Inhibition = -2.98% (TNF-α)	CURCUMIN<br>Inhibition = 50.64% (IL-6)<br>Inhibition = 18.48% (TNF-α)	[24]
**505**	Inhibition = 22.87% (IL-6)<br>Inhibition = 42.95% (TNF-α)	CURCUMIN<br>Inhibition = 50.64% (IL-6)<br>Inhibition = 18.48% (TNF-α)	[24]
**506**	Inhibition = 1.74% (IL-6)<br>Inhibition = 83.23% (TNF-α)	CURCUMIN<br>Inhibition = 50.64% (IL-6)<br>Inhibition = 18.48% (TNF-α)	[24]
**507**	Inhibition = 55.25% (IL-6)<br>Inhibition = 34.52% (TNF-α)	CURCUMIN<br>Inhibition = 50.64% (IL-6)<br>Inhibition = 18.48% (TNF-α)	[24]
**508**	Inhibition = 50.12% (IL-6)<br>Inhibition = 38.91% (TNF-α)	CURCUMIN<br>Inhibition = 50.64% (IL-6)<br>Inhibition = 18.48% (TNF-α)	[24]
**509**	Inhibition = 34.36% (IL-6)<br>Inhibition = 42.17% (TNF-α)	CURCUMIN<br>Inhibition = 50.64% (IL-6)<br>Inhibition = 18.48% (TNF-α)	[24]
**510**	Inhibition = 64.80% (IL-6)<br>Inhibition = 42.21% (TNF-α)	CURCUMIN<br>Inhibition = 50.64% (IL-6)<br>Inhibition = 18.48% (TNF-α)	[24]
**511**	Inhibition = 28.98% (IL-6)<br>Inhibition = 18.91% (TNF-α)	CURCUMIN<br>Inhibition = 50.64% (IL-6)<br>Inhibition = 18.48% (TNF-α)	[24]
**512**	Inhibition = -2.30% (IL-6)<br>Inhibition = 62.30% (TNF-α)	CURCUMIN<br>Inhibition = 50.64% (IL-6)<br>Inhibition = 18.48% (TNF-α)	[24]
**513**	Inhibition = 36.70% (IL-6)<br>Inhibition = 40.58% (TNF-α)	CURCUMIN<br>Inhibition = 50.64% (IL-6)<br>Inhibition = 18.48% (TNF-α)	[24]

**514**	Inhibition = 30.63% (IL-6)<br>Inhibition = 10.32% (TNF-α)	CURCUMIN<br>Inhibition = 50.64% (IL-6)<br>Inhibition = 18.48% (TNF-α)	[24]
**515**	Inhibition = 27.22% (IL-6)<br>Inhibition = 26.57% (TNF-α)	CURCUMIN<br>Inhibition = 50.64% (IL-6)<br>Inhibition = 18.48% (TNF-α)	[24]
**516**	Inhibition = 33.37% (IL-6)<br>Inhibition = 32.83% (TNF-α)	CURCUMIN<br>Inhibition = 50.64% (IL-6)<br>Inhibition = 18.48% (TNF-α)	[24]
**517**	Inhibition = 37.61% (IL-6)<br>Inhibition = 9.92% (TNF-α)	CURCUMIN<br>Inhibition = 50.64% (IL-6)<br>Inhibition = 18.48% (TNF-α)	[24]
**518**	Inhibition = 52.56% (IL-6)<br>Inhibition = 36.37% (TNF-α)	CURCUMIN<br>Inhibition = 50.64% (IL-6)<br>Inhibition = 18.48% (TNF-α)	[24]
**519**	Inhibition = 54.40% (IL-6)<br>Inhibition = 60.25% (TNF-α)	CURCUMIN<br>Inhibition = 50.64% (IL-6)<br>Inhibition = 18.48% (TNF-α)	[24]
**520**	Inhibition = 59.59 ± 4.9% (IL-6)<br>Inhibition = 7.15 ± 9.3% (TNF-α)	----	[25]
**521**	Inhibition = 44.36 ± 9.4% (IL-6)	----	[25]
**522**	Inhibition = 57.14 ± 7.9% (IL-6)	----	[25]
**523**	Inhibition = 67.62 ± 3.2% (IL-6)	----	[25]
**524**	Inhibition = 76.14 ± 2.5% (IL-6)	----	[25]
**525**	Inhibition = 71.59 ± 4.3% (IL-6)	----	[25]
**526**	Inhibition = 77.54 ± 4.7% (IL-6)<br>Inhibition = 39.49 ± 8.1% (TNF-α)	----	[25]
**527**	Inhibition = 24.75 ± 18.9% (IL-6)	----	[25]
**528**	Inhibition = 45.84 ± 12.6% (IL-6)	----	[25]
**529**	Inhibition = 79.64 ± 6.1% (IL-6)	----	[25]
**530**	Inhibition = 86.85 ± 5.6% (IL-6)	----	[25]
**531**	Inhibition = 89.23 ± 6.7% (IL-6)	----	[25]
**532**	Inhibition = 81.48 ± 4.9% (IL-6)	----	[25]
**533**	Inhibition = 83.26 ± 4.0% (IL-6)<br>Inhibition = 43.81 ± 2.1% (TNF-α)	----	[25]
**534**	Inhibition = 59.83 ± 6.1% (IL-6)<br>Inhibition = 8.19 ± 6.6% (TNF-α)	----	[25]
**534**	Inhibition = 49.86 ± 4.0% (IL-6)<br>Inhibition = 17.56 ± 3.6% (TNF-α)	----	[25]
**536**	Inhibition = 71.94 ± 3.6% (IL-6)<br>Inhibition = 32.15 ± 3.1% (TNF-α)	----	[25]
**537**	Inhibition = 74.40 ± 5.1% (IL-6)<br>Inhibition = 34.99 ± 3.3% (TNF-α)	----	[25]
**538**	~ 70 (relative amount of TNF-α (% of LPS))<br>~ 30 (relative amount of IL-6 (% of LPS))	RESVERATROL<br>~ 70 (relative amount of TNF-α (% of LPS))<br>~ 70 (relative amount of IL-6 (% of LPS))	[26]
**539**	~ 85 (relative amount of TNF-α (% of LPS))<br>~ 60 (relative amount of IL-6 (% of LPS))	RESVERATROL<br>~ 70 (relative amount of TNF-α (% of LPS))<br>~ 70 (relative amount of IL-6 (% of LPS))	[26]

**540**	~ 95 (relative amount of TNF-α (% of LPS))<br>~ 70 (relative amount of IL-6 (% of LPS))	RESVERATROL<br>~ 70 (relative amount of TNF-α (% of LPS))<br>~ 70 (relative amount of IL-6 (% of LPS))	[26]
**541**	~ 90 (relative amount of TNF-α (% of LPS))<br>~ 85 (relative amount of IL-6 (% of LPS))	RESVERATROL<br>~ 70 (relative amount of TNF-α (% of LPS))<br>~ 70 (relative amount of IL-6 (% of LPS))	[26]
**542**	~ 105 (relative amount of TNF-α (% of LPS))<br>~ 95 (relative amount of IL-6 (% of LPS))	RESVERATROL<br>~ 70 (relative amount of TNF-α (% of LPS))<br>~ 70 (relative amount of IL-6 (% of LPS))	[26]
**543**	~ 110 (relative amount of TNF-α (% of LPS))<br>~ 60 (relative amount of IL-6 (% of LPS))	RESVERATROL<br>~ 70 (relative amount of TNF-α (% of LPS))<br>~ 70 (relative amount of IL-6 (% of LPS))	[26]
**344**	~ 40 (relative amount of TNF-α (% of LPS))<br>~ 10(relative amount of IL-6 (% of LPS))	RESVERATROL<br>~ 70 (relative amount of TNF-α (% of LPS))<br>~ 70 (relative amount of IL-6 (% of LPS))	[26]
**545**	~ 80 (relative amount of TNF-α (% of LPS))<br>~ 70 (relative amount of IL-6 (% of LPS))	RESVERATROL<br>~ 70 (relative amount of TNF-α (% of LPS))<br>~ 70 (relative amount of IL-6 (% of LPS))	[26]
**546**	~ 110 (relative amount of TNF-α (% of LPS))<br>~ 30 (relative amount of IL-6 (% of LPS))	RESVERATROL<br>~ 70 (relative amount of TNF-α (% of LPS))<br>~ 70 (relative amount of IL-6 (% of LPS))	[26]
**547**	~ 120 (relative amount of TNF-α (% of LPS))<br>~ 20 (relative amount of IL-6 (% of LPS))	RESVERATROL<br>~ 70 (relative amount of TNF-α (% of LPS))<br>~ 70 (relative amount of IL-6 (% of LPS))	[26]
**548**	~ 130 (relative amount of TNF-α (% of LPS))<br>~ 30 (relative amount of IL-6 (% of LPS))	RESVERATROL<br>~ 70 (relative amount of TNF-α (% of LPS))<br>~ 70 (relative amount of IL-6 (% of LPS))	[26]
**549**	~ 150 (relative amount of TNF-α (% of LPS))<br>~ 30 (relative amount of IL-6 (% of LPS))	RESVERATROL<br>~ 70 (relative amount of TNF-α (% of LPS))<br>~ 70 (relative amount of IL-6 (% of LPS))	[26]
**550**	~ 55 (relative amount of TNF-α (% of LPS))<br>~ 20 (relative amount of IL-6 (% of LPS))	RESVERATROL<br>~ 70 (relative amount of TNF-α (% of LPS))<br>~ 70 (relative amount of IL-6 (% of LPS))	[26]
**551**	~ 70 (relative amount of TNF-α (% of LPS))<br>~ 50 (relative amount of IL-6 (% of LPS))	RESVERATROL<br>~ 70 (relative amount of TNF-α (% of LPS))<br>~ 70 (relative amount of IL-6 (% of LPS))	[26]
**552**	~ 80 (relative amount of TNF-α (% of LPS))<br>~ 55 (relative amount of IL-6 (% of LPS))	RESVERATROL<br>~ 70 (relative amount of TNF-α (% of LPS))<br>~ 70 (relative amount of IL-6 (% of LPS))	[26]

**553**	~ 80 (relative amount of TNF-α (% of LPS))<br>~ 70 (relative amount of IL-6 (% of LPS))	RESVERATROL<br>~ 70 (relative amount of TNF-α (% of LPS))<br>~ 70 (relative amount of IL-6 (% of LPS))	[26]
**554**	~ 80 (relative amount of TNF-α (% of LPS))<br>~ 50 (relative amount of IL-6 (% of LPS))	RESVERATROL<br>~ 70 (relative amount of TNF-α (% of LPS))<br>~ 70 (relative amount of IL-6 (% of LPS))	[26]
**555**	~ 90 (relative amount of TNF-α (% of LPS))<br>~ 90 (relative amount of IL-6 (% of LPS))	RESVERATROL<br>~ 70 (relative amount of TNF-α (% of LPS))<br>~ 70 (relative amount of IL-6 (% of LPS))	[26]
**556**	~ 90 (relative amount of TNF-α (% of LPS))<br>~ 45 (relative amount of IL-6 (% of LPS))	RESVERATROL<br>~ 70 (relative amount of TNF-α (% of LPS))<br>~ 70 (relative amount of IL-6 (% of LPS))	[26]
**557**	~ 70 (relative amount of TNF-α (% of LPS))<br>~ 10 (relative amount of IL-6 (% of LPS))	RESVERATROL<br>~ 70 (relative amount of TNF-α (% of LPS))<br>~ 70 (relative amount of IL-6 (% of LPS))	[26]
**558**	~ 45 (relative amount of TNF-α (% of LPS))<br>~ 5 (relative amount of IL-6 (% of LPS))	RESVERATROL<br>~ 70 (relative amount of TNF-α (% of LPS))<br>~ 70 (relative amount of IL-6 (% of LPS))	[26]
**559**	~ 20 (relative amount of TNF-α (% of LPS))<br>~ 10 (relative amount of IL-6 (% of LPS))	RESVERATROL<br>~ 70 (relative amount of TNF-α (% of LPS))<br>~ 70 (relative amount of IL-6 (% of LPS))	[26]
**560**	~ 60 (relative amount of TNF-α (% of LPS))<br>~ 10 (relative amount of IL-6 (% of LPS))	RESVERATROL<br>~ 70 (relative amount of TNF-α (% of LPS))<br>~ 70 (relative amount of IL-6 (% of LPS))	[26]
**561**	~ 70 (relative amount of TNF-α (% of LPS))<br>~ 50 (relative amount of IL-6 (% of LPS))	RESVERATROL<br>~ 70 (relative amount of TNF-α (% of LPS))<br>~ 70 (relative amount of IL-6 (% of LPS))	[26]
**562**	~ 55 (relative amount of TNF-α (% of LPS))<br>~ 30 (relative amount of IL-6 (% of LPS))	RESVERATROL<br>~ 70 (relative amount of TNF-α (% of LPS))<br>~ 70 (relative amount of IL-6 (% of LPS))	[26]
**563**	~ 50 (relative amount of TNF-α (% of LPS))<br>~ 10 (relative amount of IL-6 (% of LPS))	RESVERATROL<br>~ 70 (relative amount of TNF-α (% of LPS))<br>~ 70 (relative amount of IL-6 (% of LPS))	[26]
**564**	~ 55 (relative amount of TNF-α (% of LPS))<br>~ 5 (relative amount of IL-6 (% of LPS))	RESVERATROL<br>~ 70 (relative amount of TNF-α (% of LPS))<br>~ 70 (relative amount of IL-6 (% of LPS))	[26]
**Regulation of COX and LOX pathways**			
**Compound**		**Reference Drug**	**Reference**
**23**	Inhibition < 50 % (COX-1 at 100 μg/ml, TMPD assay)	CURCUMIN	[27]

	Inhibition = 0 % (COX-2 at 50 μg/ml, HPLC assay)	Inhibition > 50 % (COX-1 at 100 μg/ml, TMPD assay)<br>Inhibition = 27% (COX-1 at 70 μg/ml, O_2-consumption assay<br>Inhibition = 71% (COX-2 at 50 μg/ml, HPLC assay)	
**29**	Inhibition < 50 % (COX-1 at 100 μg/ml, TMPD assay)<br>Inhibition = 60 % (COX-2 at 50 μg/ml, HPLC assay)	CURCUMIN<br>Inhibition > 50% (COX-1 at 100 μg/ml, TMPD assay)<br>Inhibition = 71% (COX-2 at 50 μg/ml, HPLC assay)	[27]
**33**	Inhibition < 50 % (COX-1 at 100 μg/ml, TMPD assay)<br>Inhibition = 39 % (COX-2 at 50 μg/ml, HPLC assay)	CURCUMIN<br>Inhibition > 50% (COX-1 at 100 μg/ml, TMPD assay)<br>Inhibition = 71% (COX-2 at 50 μg/ml, HPLC assay)	[27]
**34**	Inhibition > 50 % (COX-1 at 100 μg/ml, TMPD assay)<br>Inhibition = 27% (COX-1 at 70 μg/ml, O_2-consumption assay<br>Inhibition = 56 % (COX-2 at 50 μg/ml, HPLC assay)	CURCUMIN<br>Inhibition > 50 % (COX-1 at 100 μg/ml, TMPD assay)<br>Inhibition = 27% (COX-1 at 70 μg/ml, O_2-consumption assay<br>Inhibition = 71% (COX-2 at 50 μg/ml, HPLC assay)	[27]
**114**	Inhibition < 50 % (COX-1 at 100 μg/ml, TMPD assay)<br>Inhibition = 15 % (COX-2 at 50 μg/ml, HPLC assay)	CURCUMIN<br>Inhibition > 50% (COX-1 at 100 μg/ml, TMPD assay)<br>Inhibition = 71% (COX-2 at 50 μg/ml, HPLC assay)	[27]
**129**	Inhibition < 50 % (COX-1 at 100 μg/ml, TMPD assay)<br>Inhibition = 12 % (COX-2 at 50 μg/ml, HPLC assay)	CURCUMIN<br>Inhibition > 50% (COX-1 at 100 μg/ml, TMPD assay)<br>Inhibition = 71% (COX-2 at 50 μg/ml, HPLC assay)	[27]
**131**	Inhibition < 50 % (COX-1 at 100 μg/ml, TMPD assay)<br>Inhibition = 2 % (COX-2 at 50 μg/ml, HPLC assay)	CURCUMIN<br>Inhibition > 50% (COX-1 at 100 μg/ml, TMPD assay)<br>Inhibition = 71% (COX-2 at 50 μg/ml, HPLC assay)	[27]
**165**	Inhibition > 50 % (COX-1 at 100 μg/ml, TMPD assay)<br>Inhibition = 20% (COX-1 at 70 μg/ml, O_2-consumption assay<br>Inhibition = 53 % (COX-2 at 50 μg/ml, HPLC assay)	CURCUMIN<br>Inhibition > 50 % (COX-1 at 100 μg/ml, TMPD assay)<br>Inhibition = 27% (COX-1 at 70 μg/ml, O_2-consumption assay<br>Inhibition = 71% (COX-2 at 50 μg/ml, HPLC assay)	[27]
**379**	Inhibition < 50 % (COX-1 at 100 μg/ml, TMPD assay)<br>Inhibition = 4 % (COX-2 at 50 μg/ml, HPLC assay)	CURCUMIN<br>Inhibition > 50% (COX-1 at 100 μg/ml, TMPD assay)<br>Inhibition = 71% (COX-2 at 50 μg/ml, HPLC assay)	[27]
**565**	Inhibition < 50 % (COX-1 at 100 μg/ml, TMPD assay)	CURCUMIN	[27]

	Inhibition = 24% (COX-2 at 50 μg/ml, HPLC assay)	Inhibition > 50% (COX-1 at 100 μg/ml, TMPD assay)<br>Inhibition = 71% (COX-2 at 50 μg/ml, HPLC assay)	
**566**	Inhibition < 50 % (COX-1 at 100 μg/ml, TMPD assay)<br>Inhibition = 38% (COX-2 at 50 μg/ml, HPLC assay)	CURCUMIN<br>Inhibition > 50% (COX-1 at 100 μg/ml, TMPD assay)<br>Inhibition = 71% (COX-2 at 50 μg/ml, HPLC assay)	[27]
**567**	Inhibition > 50 % (COX-1 at 100 μg/ml, TMPD assay)<br>Inhibition = 56% (COX-1 at 70 μg/ml, O_2-consumption assay<br>Inhibition = 95% (COX-2 at 50 μg/ml, HPLC assay)<br>IC_{50} = 12.1 μM (COX-1)<br>IC_{50} = 5.5 ± 2.1 μM (COX-2)	CURCUMIN<br>Inhibition > 50 % (COX-1 at 100 μg/ml, TMPD assay)<br>Inhibition = 27% (COX-1 at 70 μg/ml, O_2-consumption assay<br>Inhibition = 71% (COX-2 at 50 μg/ml, HPLC assay)<br>IC_{50} = 18.8 μM (COX-1)<br>IC_{50} = 15.9 ± 7.9 μM (COX-2)	[27]
**568**	Inhibition = 8% (LOX at 100μM)	CURCUMIN<br>Inhibition = 38% (LOX at 100μM)	[28]
**569**	IC_{50} = 37 μM (LOX)	CURCUMIN<br>Inhibition = 38% (LOX at 100μM)	[28]
**570**	IC_{50} = 330 μM (LOX)	CURCUMIN<br>Inhibition = 38% (LOX at 100μM)	[28]
**571**	IC_{50} = 280 μM (LOX)	CURCUMIN<br>Inhibition = 38% (LOX at 100μM)	[28]
**572**	IC_{50} = 410 μM (LOX)	CURCUMIN<br>Inhibition = 38% (LOX at 100μM)	[28]
**573**	Inhibition = 18% (LOX at 100μM)	CURCUMIN<br>Inhibition = 38% (LOX at 100μM)	[28]
**574**	Inhibition = 36% (LOX at 100μM)	CURCUMIN<br>Inhibition = 38% (LOX at 100μM)	[28]
**575**	Not inhibition	CURCUMIN<br>Inhibition = 38% (LOX at 100μM)	[28]
**576**	Not inhibition	CURCUMIN<br>Inhibition = 38% (LOX at 100μM)	[28]
**577**	IC_{50} = 47 μM (LOX)	CURCUMIN<br>Inhibition = 38% (LOX at 100μM)	[28]
**217**	IC_{50} = 33.33 ± 0.17 μM (COX-1)<br>IC_{50} = 53.23 ± 1.27 μM (COX-2)<br>IC_{50} = 47.83 ± 0.51 μM (LOX)<br>IC_{50} = 7.34 ± 0.76 μM (mPGES-1)	CURCUMIN<br>IC_{50} = 31.31 ± 0.45 μM (COX-1)<br>IC_{50} = 96.36 ± 1.43 μM (COX-2)<br>IC_{50} = 57.77 ± 0.21 μM (LOX)<br>IC_{50} = 4.88 ± 0.54 μM (mPGES-1)<br>INDOMETRACIN<br>IC_{50} = 0.21 ± 0.03 μM (COX-1)<br>IC_{50} = 3.24 ± 0.01 μM (COX-2)	[29]
**328**	IC_{50} = 41.51 ± 0.49 μM (COX-1)<br>IC_{50} = 70.71 ± 1.36 μM (COX-2)<br>IC_{50} = 62.86 ± 1.69 μM (LOX)<br>IC_{50} = 14.75 ± 2.25 μM (mPGES-1)	CURCUMIN<br>IC_{50} = 31.31 ± 0.45 μM (COX-1)<br>IC_{50} = 96.36 ± 1.43 μM (COX-2)<br>IC_{50} = 57.77 ± 0.21 μM (LOX)<br>IC_{50} = 4.88 ± 0.54 μM (mPGES-1)<br>INDOMETRACIN	[29]

		IC_{50} = 0.21 ± 0.03 μM (COX-1)<br>IC_{50} = 3.24 ± 0.01 μM (COX-2)	
**362**	IC_{50} = 40.24 ± 0.36μM (COX-1)<br>IC_{50} = 63.76 ± 0.74 μM (LOX)<br>IC_{50} = 10.43 ± 1.84 μM (mPGES-1)	CURCUMIN<br>IC_{50} = 31.31 ± 0.45 μM (COX-1)<br>IC_{50} = 57.77 ± 0.21 μM (LOX)<br>IC_{50} = 4.88 ± 0.54 μM (mPGES-1)<br>INDOMETRACIN<br>IC_{50} = 0.21 ± 0.03 μM (COX-1)	[29]
**578**	IC_{50} = 22.08 ± 0.42 μM (COX-1)<br>IC_{50} = 46.55 ± 1.35 μM (COX-2)<br>IC_{50} = 82.44 ± 0.71 μM (LOX)<br>IC_{50} = 4.96 ± 0.47 μM (mPGES-1)	CURCUMIN<br>IC_{50} = 31.31 ± 0.45 μM (COX-1)<br>IC_{50} = 96.36 ± 1.43 μM (COX-2)<br>IC_{50} = 57.77 ± 0.21 μM (LOX)<br>IC_{50} = 4.88 ± 0.54 μM (mPGES-1)<br>INDOMETRACIN<br>IC_{50} = 0.21 ± 0.03 μM (COX-1)<br>IC_{50} = 3.24 ± 0.01 μM (COX-2)	[29]
**579**	IC_{50} = 43.22 ± 0.28 μM (COX-1)<br>IC_{50} = 8.95 ± 1.03 μM (mPGES-1)	CURCUMIN<br>IC_{50} = 31.31 ± 0.45 μM (COX-1)<br>IC_{50} = 4.88 ± 0.54 μM (mPGES-1)<br>INDOMETRACIN<br>IC_{50} = 0.21 ± 0.03 μM (COX-1)	[29]
**580**	IC_{50} = 38.52 ± 0.48 μM (COX-1)<br>IC_{50} = 42.41 ± 0.24 μM (mPGES-1)	CURCUMIN<br>IC_{50} = 31.31 ± 0.45 μM (COX-1)<br>IC_{50} = 4.88 ± 0.54 μM (mPGES-1)<br>INDOMETRACIN<br>IC_{50} = 0.21 ± 0.03 μM (COX-1)	[29]
**581**	IC_{50} = 16.65 ± 0.35 μM (COX-1)<br>IC_{50} = 57.57 ± 1.37 μM (COX-2)<br>IC_{50} = 30.01 ± 0.21 μM (LOX)<br>IC_{50} = 4.48 ± 0.80 μM (mPGES-1)	CURCUMIN<br>IC_{50} = 31.31 ± 0.45 μM (COX-1)<br>IC_{50} = 96.36 ± 1.43 μM (COX-2)<br>IC_{50} = 57.77 ± 0.21 μM (LOX)<br>IC_{50} = 4.88 ± 0.54 μM (mPGES-1)<br>INDOMETRACIN<br>IC_{50} = 0.21 ± 0.03 μM (COX-1)<br>IC_{50} = 3.24 ± 0.01 μM (COX-2)	[29]
**582**	IC_{50} = 23.62 ± 0.38 μM (COX-1)<br>IC_{50} = 58.57 ± 1.36 μM (COX-2)<br>IC_{50} = 56.42 ± 0.52 μM (LOX)<br>IC_{50} = 4.23 ± 0.26 μM (mPGES-1)	CURCUMIN<br>IC_{50} = 31.31 ± 0.45 μM (COX-1)<br>IC_{50} = 96.36 ± 1.43 μM (COX-2)<br>IC_{50} = 57.77 ± 0.21 μM (LOX)<br>IC_{50} = 4.88 ± 0.54 μM (mPGES-1)<br>INDOMETRACIN<br>IC_{50} = 0.21 ± 0.03 μM (COX-1)<br>IC_{50} = 3.24 ± 0.01 μM (COX-2)	[29]
**583**	IC_{50} = 53.24 ± 0.76 μM (COX-1)<br>IC_{50} = 36.99 ± 4.32 μM (mPGES-1)	CURCUMIN<br>IC_{50} = 31.31 ± 0.45 μM (COX-1)<br>IC_{50} = 4.88 ± 0.54 μM (mPGES-1)<br>INDOMETRACIN<br>IC_{50} = 0.21 ± 0.03 μM (COX-1)	[29]
**584**	IC_{50} = 37.70 ± 0.31 μM (COX-1)<br>IC_{50} = 95.07 ± 4.24 μM (COX-2)<br>IC_{50} = 6.98 ± 0.79 μM (mPGES-1)	CURCUMIN<br>IC_{50} = 31.31 ± 0.45 μM (COX-1)<br>IC_{50} = 96.36 ± 1.43 μM (COX-2)<br>IC_{50} = 4.88 ± 0.54 μM (mPGES-1)<br>INDOMETRACIN	[29]

		IC_{50} = 0.21 ± 0.03 μM (COX-1)<br>IC_{50} = 3.24 ± 0.01 μM (COX-2)	
**585**	IC_{50} = 17.40 ± 0.60 μM (COX-1)<br>IC_{50} = 46.11 ± 1.48 μM (COX-2)<br>IC_{50} = 41.13 ± 0.43 μM (LOX)<br>IC_{50} = 7.89 ± 0.21 μM (mPGES-1)	CURCUMIN<br>IC_{50} = 31.31 ± 0.45 μM (COX-1)<br>IC_{50} = 96.36 ± 1.43 μM (COX-2)<br>IC_{50} = 57.77 ± 0.21 μM (LOX)<br>IC_{50} = 4.88 ± 0.54 μM (mPGES-1)<br>INDOMETRACIN<br>IC_{50} = 0.21 ± 0.03 μM (COX-1)<br>IC_{50} = 3.24 ± 0.01 μM (COX-2)	[29]
**586**	IC_{50} = 35.86 ± 0.24 μM (COX-1)<br>IC_{50} = 89.51 ± 0.98 μM (LOX)<br>IC_{50} = 3.78 ± 0.24 μM (mPGES-1)	CURCUMIN<br>IC_{50} = 31.31 ± 0.45 μM (COX-1)<br>IC_{50} = 57.77 ± 0.21 μM (LOX)<br>IC_{50} = 4.88 ± 0.54 μM (mPGES-1)<br>INDOMETRACIN<br>IC_{50} = 0.21 ± 0.03 μM (COX-1)	[29]
**587**	IC_{50} = 60.01 ± 0.97 μM (COX-1)<br>IC_{50} = 24.48 ± 3.22 μM (mPGES-1)	CURCUMIN<br>IC_{50} = 31.31 ± 0.45 μM (COX-1)<br>IC_{50} = 4.88 ± 0.54 μM (mPGES-1)<br>INDOMETRACIN<br>IC_{50} = 0.21 ± 0.03 μM (COX-1)	[29]
**588**	IC_{50} = 31.95 ± 0.54 μM (COX-1)<br>IC_{50} = 56.76 ± 0.74 μM (LOX)<br>IC_{50} = 5.48 ± 0.74 μM (mPGES-1)	CURCUMIN<br>IC_{50} = 31.31 ± 0.45 μM (COX-1)<br>IC_{50} = 57.77 ± 0.21 μM (LOX)<br>IC_{50} = 4.88 ± 0.54 μM (mPGES-1)<br>INDOMETRACIN<br>IC_{50} = 0.21 ± 0.03 μM (COX-1)	[29]
**589**	IC_{50} = 194.0 ± 3.14 μM (COX-1)<br>IC_{50} = 20.27 ± 2.11 μM (mPGES-1)	CURCUMIN<br>IC_{50} = 31.31 ± 0.45 μM (COX-1)<br>IC_{50} = 4.88 ± 0.54 μM (mPGES-1)<br>INDOMETRACIN<br>IC_{50} = 0.21 ± 0.03 μM (COX-1)	[29]
**590**	IC_{50} = 194.6 ± 2.40 μM (COX-1)<br>IC_{50} = 60.71 ± 5.85 μM (mPGES-1)	CURCUMIN<br>IC_{50} = 31.31 ± 0.45 μM (COX-1)<br>IC_{50} = 4.88 ± 0.54 μM (mPGES-1)<br>INDOMETRACIN<br>IC_{50} = 0.21 ± 0.03 μM (COX-1)	[29]
**591**	IC_{50} = 60.01 ± 0.97 μM (COX-1)<br>IC_{50} = 7.48 ± 1.89 μM (mPGES-1)	CURCUMIN<br>IC_{50} = 31.31 ± 0.45 μM (COX-1)<br>IC_{50} = 4.88 ± 0.54 μM (mPGES-1)<br>INDOMETRACIN<br>IC_{50} = 0.21 ± 0.03 μM (COX-1)	[29]
**592**	Inhibition = 78% (PGE_2 at 25 μM in RAW 264.7)<br>Inhibition = 88% (PGE_2 at 25 μM in U937)	CURCUMIN<br>Inhibition = 78% (PGE_2 at 25 μM in RAW 264.7)<br>Inhibition = 94% (PGE_2 at 25 μM in U937)<br>NIMESULIDE<br>Inhibition = 66% (PGE_2 at 25 μM in RAW 264.7)<br>Inhibition = 89% (PGE_2 at 25 μM in U937)	[30]
**593**	Inhibition = 83% (PGE_2 at 25 μM in RAW 264.7)	CURCUMIN	[30]

	Inhibition = 83% (PGE_2 at 25 μM in U937)<br>IC_{50} = 12.01 μM (RAW 264.7)<br>IC_{50} = 3.44 μM (U937)	Inhibition = 78% (PGE_2 at 25 μM in RAW 264.7)<br>Inhibition = 94% (PGE_2 at 25 μM in U937)<br>IC_{50} = 15.95 μM (RAW 264.7)<br>IC_{50} = 1.88 μM (U937)<br>NIMESULIDE<br>Inhibition = 66% (PGE_2 at 25 μM in RAW 264.7)<br>Inhibition = 89% (PGE_2 at 25 μM in U937)<br>IC_{50} < 0.078 μM (RAW 264.7)<br>IC_{50} < 0.078 μM (U937)	
**594**	Inhibition = 92% (PGE_2 at 25 μM in RAW 264.7)<br>Inhibition = 84% (PGE_2 at 25 μM in U937)<br>IC_{50} = 4.86 μM (RAW 264.7)<br>IC_{50} = 1.65 μM (U937)	CURCUMIN<br>Inhibition = 78% (PGE_2 at 25 μM in RAW 264.7)<br>Inhibition = 94% (PGE_2 at 25 μM in U937)<br>IC_{50} = 15.95 μM (RAW 264.7)<br>IC_{50} = 1.88 μM (U937)<br>NIMESULIDE<br>Inhibition = 66% (PGE_2 at 25 μM in RAW 264.7)<br>Inhibition = 89% (PGE_2 at 25 μM in U937)<br>IC_{50} < 0.078 μM (RAW 264.7)<br>IC_{50} < 0.078 μM (U937)	[30]
**595**	Inhibition = 74% (PGE_2 at 25 μM in RAW 264.7)<br>Inhibition = 66% (PGE_2 at 25 μM in U937)	CURCUMIN<br>Inhibition = 78% (PGE_2 at 25 μM in RAW 264.7)<br>Inhibition = 94% (PGE_2 at 25 μM in U937)<br>NIMESULIDE<br>Inhibition = 66% (PGE_2 at 25 μM in RAW 264.7)<br>Inhibition = 89% (PGE_2 at 25 μM in U937)	[30]
**596**	Inhibition = 73% (PGE_2 at 25 μM in RAW 264.7)<br>Inhibition = 58% (PGE_2 at 25 μM in U937)	CURCUMIN<br>Inhibition = 78% (PGE_2 at 25 μM in RAW 264.7)<br>Inhibition = 94% (PGE_2 at 25 μM in U937)<br>NIMESULIDE<br>Inhibition = 66% (PGE_2 at 25 μM in RAW 264.7)<br>Inhibition = 89% (PGE_2 at 25 μM in U937)	[30]
**597**	Inhibition = 51% (PGE_2 at 25 μM in RAW 264.7)<br>Inhibition = 68% (PGE_2 at 25 μM in U937)	CURCUMIN<br>Inhibition = 78% (PGE_2 at 25 μM in RAW 264.7)<br>Inhibition = 94% (PGE_2 at 25 μM in U937)<br>NIMESULIDE<br>Inhibition = 66% (PGE_2 at 25 μM in RAW 264.7)<br>Inhibition = 89% (PGE_2 at 25 μM in U937)	[30]
**598**	Inhibition = 59% (PGE_2 at 25 μM in RAW 264.7)<br>Inhibition = 76% (PGE_2 at 25 μM in U937)	CURCUMIN<br>Inhibition = 78% (PGE_2 at 25 μM in RAW 264.7)<br>Inhibition = 94% (PGE_2 at 25 μM in U937)<br>NIMESULIDE	[30]

		Inhibition = 66% (PGE_2 at 25 μM in RAW 264.7)<br>Inhibition = 89% (PGE_2 at 25 μM in U937)	
**599**	Inhibition = 53% (PGE_2 at 25 μM in RAW 264.7)<br>Inhibition = 66% (PGE_2 at 25 μM in U937)	CURCUMIN<br>Inhibition = 78% (PGE_2 at 25 μM in RAW 264.7)<br>Inhibition = 94% (PGE_2 at 25 μM in U937)<br>NIMESULIDE<br>Inhibition = 66% (PGE_2 at 25 μM in RAW 264.7)<br>Inhibition = 89% (PGE_2 at 25 μM in U937)	[30]
**600**	Inhibition = 65% (PGE_2 at 25 μM in RAW 264.7)<br>Inhibition = 72% (PGE_2 at 25 μM in U937)	CURCUMIN<br>Inhibition = 78% (PGE_2 at 25 μM in RAW 264.7)<br>Inhibition = 94% (PGE_2 at 25 μM in U937)<br>NIMESULIDE<br>Inhibition = 66% (PGE_2 at 25 μM in RAW 264.7)<br>Inhibition = 89% (PGE_2 at 25 μM in U937)	[30]
**601**	Inhibition = 96% (PGE_2 at 25 μM in RAW 264.7)<br>Inhibition = 86% (PGE_2 at 25 μM in U937)<br>IC_{50} = 0.78 μM (RAW 264.7)<br>IC_{50} = 0.95 μM (U937)	CURCUMIN<br>Inhibition = 78% (PGE_2 at 25 μM in RAW 264.7)<br>Inhibition = 94% (PGE_2 at 25 μM in U937)<br>IC_{50} = 15.95 μM (RAW 264.7)<br>IC_{50} = 1.88 μM (U937)<br>NIMESULIDE<br>Inhibition = 66% (PGE_2 at 25 μM in RAW 264.7)<br>Inhibition = 89% (PGE_2 at 25 μM in U937)<br>IC_{50} < 0.078 μM (RAW 264.7)<br>IC_{50} < 0.078 μM (U937)	[30]
**602**	Inhibition = 96% (PGE_2 at 25 μM in RAW 264.7)<br>Inhibition = 88% (PGE_2 at 25 μM in U937)<br>IC_{50} = 1.9 μM (RAW 264.7)<br>IC_{50} = 0.92 μM (U937)	CURCUMIN<br>Inhibition = 78% (PGE_2 at 25 μM in RAW 264.7)<br>Inhibition = 94% (PGE_2 at 25 μM in U937)<br>IC_{50} = 15.95 μM (RAW 264.7)<br>IC_{50} = 1.88 μM (U937)<br>NIMESULIDE<br>Inhibition = 66% (PGE_2 at 25 μM in RAW 264.7)<br>Inhibition = 89% (PGE_2 at 25 μM in U937)<br>IC_{50} < 0.078 μM (RAW 264.7)<br>IC_{50} < 0.078 μM (U937)	[30]
**603**	Inhibition = 95% (PGE_2 at 25 μM in RAW 264.7)<br>Inhibition = 85% (PGE_2 at 25 μM in U937)	CURCUMIN<br>Inhibition = 78% (PGE_2 at 25 μM in RAW 264.7)<br>Inhibition = 94% (PGE_2 at 25 μM in U937)<br>NIMESULIDE<br>Inhibition = 66% (PGE_2 at 25 μM in RAW 264.7)<br>Inhibition = 89% (PGE_2 at 25 μM in U937)	[30]

**604**	Inhibition = 96% (PGE_2 at 25 μM in RAW 264.7)<br>Inhibition = 87% (PGE_2 at 25 μM in U937)	CURCUMIN<br>Inhibition = 78% (PGE_2 at 25 μM in RAW 264.7)<br>Inhibition = 94% (PGE_2 at 25 μM in U937)<br>NIMESULIDE<br>Inhibition = 66% (PGE_2 at 25 μM in RAW 264.7)<br>Inhibition = 89% (PGE_2 at 25 μM in U937)	[30]
**605**	Inhibition = 95% (PGE_2 at 25 μM in RAW 264.7)<br>Inhibition = 85% (PGE_2 at 25 μM in U937)	CURCUMIN<br>Inhibition = 78% (PGE_2 at 25 μM in RAW 264.7)<br>Inhibition = 94% (PGE_2 at 25 μM in U937)<br>NIMESULIDE<br>Inhibition = 66% (PGE_2 at 25 μM in RAW 264.7)<br>Inhibition = 89% (PGE_2 at 25 μM in U937)	[30]
**ANTI-DIABETIC ACTIVITY**			
**Compound**		**Reference Drug**	**Reference**
**8**	IC_{50} = 21.8 μM (α-glucosidase)	CURCUMIN<br>IC_{50} = 37.2 μM (α-glucosidase)<br>ACARBOSE<br>IC_{50} = 56.2 μM (α-glucosidase)	[31]
**9**	IC_{50} = 37.0 μM (α-glucosidase)	CURCUMIN<br>IC_{50} = 37.2 μM (α-glucosidase)<br>ACARBOSE<br>IC_{50} = 56.2 μM (α-glucosidase)	[31]
**22**	IC_{50} = 32.5 μM (α-glucosidase)	CURCUMIN<br>IC_{50} = 37.2 μM (α-glucosidase)<br>ACARBOSE<br>IC_{50} = 56.2 μM (α-glucosidase)	[31]
**23**	IC_{50} = 52.8 μM (α-glucosidase)	CURCUMIN<br>IC_{50} = 37.2 μM (α-glucosidase)<br>ACARBOSE<br>IC_{50} = 56.2 μM (α-glucosidase)	[31]
**33**	IC_{50} = 37.6 μM (α-glucosidase)	CURCUMIN<br>IC_{50} = 37.2 μM (α-glucosidase)<br>ACARBOSE<br>IC_{50} = 56.2 μM (α-glucosidase)	[31]
**34**	IC_{50} = 47.0 μM (α-glucosidase)	CURCUMIN<br>IC_{50} = 37.2 μM (α-glucosidase)<br>ACARBOSE<br>IC_{50} = 56.2 μM (α-glucosidase)	[31]
**110**	IC_{50} >100 μM (α-glucosidase)	CURCUMIN<br>IC_{50} = 37.2 μM (α-glucosidase)<br>ACARBOSE<br>IC_{50} = 56.2 μM (α-glucosidase)	[31]
**111**	IC_{50} = 2.6 μM (α-glucosidase)	CURCUMIN<br>IC_{50} = 37.2 μM (α-glucosidase)<br>ACARBOSE<br>IC_{50} = 56.2 μM (α-glucosidase)	[31]
**112**	IC_{50} > 100 μM (α-glucosidase)	CURCUMIN<br>IC_{50} = 37.2 μM (α-glucosidase)	[31]

		ACARBOSE<br>IC_{50} = 56.2 μM (α-glucosidase)	
**113**	IC_{50} > 100 μM (α-glucosidase)	CURCUMIN<br>IC_{50} = 37.2 μM (α-glucosidase)<br>ACARBOSE<br>IC_{50} = 56.2 μM (α-glucosidase)	[31]
**114**	IC_{50} > 100 μM (α-glucosidase)	CURCUMIN<br>IC_{50} = 37.2 μM (α-glucosidase)<br>ACARBOSE<br>IC_{50} = 56.2 μM (α-glucosidase)	[31]
**115**	IC_{50} = 33.9 μM (α-glucosidase)	CURCUMIN<br>IC_{50} = 37.2 μM (α-glucosidase)<br>ACARBOSE<br>IC_{50} = 56.2 μM (α-glucosidase)	[31]
**120**	IC_{50} = 1.6 μM (α-glucosidase)	CURCUMIN<br>IC_{50} = 37.2 μM (α-glucosidase)<br>ACARBOSE<br>IC_{50} = 56.2 μM (α-glucosidase)	[31]
**121**	IC_{50} > 100 μM (α-glucosidase)	CURCUMIN<br>IC_{50} = 37.2 μM (α-glucosidase)<br>ACARBOSE<br>IC_{50} = 56.2 μM (α-glucosidase)	[31]
**122**	IC_{50} > 100 μM (α-glucosidase)	CURCUMIN<br>IC_{50} = 37.2 μM (α-glucosidase)<br>ACARBOSE<br>IC_{50} = 56.2 μM (α-glucosidase)	[31]
**128**	IC_{50} = 2.8 μM (α-glucosidase)	CURCUMIN<br>IC_{50} = 37.2 μM (α-glucosidase)<br>ACARBOSE<br>IC_{50} = 56.2 μM (α-glucosidase)	[31]
**129**	IC_{50} > 100 μM (α-glucosidase)	CURCUMIN<br>IC_{50} = 37.2 μM (α-glucosidase)<br>ACARBOSE<br>IC_{50} = 56.2 μM (α-glucosidase)	[31]
**130**	IC_{50} > 100 μM (α-glucosidase)	CURCUMIN<br>IC_{50} = 37.2 μM (α-glucosidase)<br>ACARBOSE<br>IC_{50} = 56.2 μM (α-glucosidase)	[31]
**131**	IC_{50} > 100 μM (α-glucosidase)	CURCUMIN<br>IC_{50} = 37.2 μM (α-glucosidase)<br>ACARBOSE<br>IC_{50} = 56.2 μM (α-glucosidase)	[31]
**137**	IC_{50} = 61.3 μM (α-glucosidase)	CURCUMIN<br>IC_{50} = 37.2 μM (α-glucosidase)<br>ACARBOSE<br>IC_{50} = 56.2 μM (α-glucosidase)	[31]
**138**	IC_{50} = 8.2 μM (α-glucosidase)	CURCUMIN<br>IC_{50} = 37.2 μM (α-glucosidase)<br>ACARBOSE<br>IC_{50} = 56.2 μM (α-glucosidase)	[31]
**139**	IC_{50} > 100 μM (α-glucosidase)	CURCUMIN<br>IC_{50} = 37.2 μM (α-glucosidase)	[31]

		ACARBOSE<br>IC_{50} = 56.2 μM (α-glucosidase)	
**140**	IC_{50} > 100 μM (α-glucosidase)	CURCUMIN<br>IC_{50} = 37.2 μM (α-glucosidase)<br>ACARBOSE<br>IC_{50} = 56.2 μM (α-glucosidase)	[31]
**141**	IC_{50} > 100 μM (α-glucosidase)	CURCUMIN<br>IC_{50} = 37.2 μM (α-glucosidase)<br>ACARBOSE<br>IC_{50} = 56.2 μM (α-glucosidase)	[31]
**142**	IC_{50} > 100 μM (α-glucosidase)	CURCUMIN<br>IC_{50} = 37.2 μM (α-glucosidase)<br>ACARBOSE<br>IC_{50} = 56.2 μM (α-glucosidase)	[31]
**143**	IC_{50} = 38.1 μM (α-glucosidase)	CURCUMIN<br>IC_{50} = 37.2 μM (α-glucosidase)<br>ACARBOSE<br>IC_{50} = 56.2 μM (α-glucosidase)	[31]
**23**	improved the glucose tolerance (at 25 mg/kg)<br>reduced the AUC glucose (at 25 mg/kg)<br>reduced SG leve (at 25 mg/kg)<br>exhibited significant reduction in all tested lipid parameters (at 25 mg/kg)		[32]
**6**	IC_{50} = 422.5 ± 25.6 (11β-HSD1 in rat testis)<br>IC_{50} = 1400.2 ± 369.4 (11β-HSD1 in human liver)<br>IC_{50} > 100000 (11β-HSD2 in rat testis)<br>IC_{50} > 100000 (11β-HSD2 in human liver)	CURCUMIN<br>IC_{50} = 11,360.8 ± 1807.9 (11β-HSD1 in rat testis)<br>IC_{50} = 5236.4 ± 98.6 (11β-HSD1 in human liver)<br>IC_{50} 12,231 ± 874.2 (11β-HSD2 in rat testis)<br>IC_{50} 14,672 ± 1136.1 (11β-HSD2 in human liver)	[33]
**606**	IC_{50} = 1638.1 ± 384.7 (11β-HSD1 in rat testis)<br>IC_{50} = 11,560.3 ± 2108.8 (11β-HSD1 in human liver)<br>IC_{50} > 100000 (11β-HSD2 in rat testis)<br>IC_{50} > 100000 (11β-HSD2 in human liver)	CURCUMIN<br>IC_{50} = 11,360.8 ± 1807.9 (11β-HSD1 in rat testis)<br>IC_{50} = 5236.4 ± 98.6 (11β-HSD1 in human liver)<br>IC_{50} 12,231 ± 874.2 (11β-HSD2 in rat testis)<br>IC_{50} 14,672 ± 1136.1 (11β-HSD2 in human liver)	[33]
**607**	IC_{50} = 1793.8 ± 110.2 (11β-HSD1 in rat testis)<br>IC_{50} > 100000 (11β-HSD1 in human liver)<br>IC_{50} > 100000 (11β-HSD2 in rat testis)<br>IC_{50} > 100000 (11β-HSD2 in human liver)	CURCUMIN<br>IC_{50} = 11,360.8 ± 1807.9 (11β-HSD1 in rat testis)<br>IC_{50} = 5236.4 ± 98.6 (11β-HSD1 in human liver)<br>IC_{50} 12,231 ± 874.2 (11β-HSD2 in rat testis)<br>IC_{50} 14,672 ± 1136.1 (11β-HSD2 in human liver)	[33]
**608**	IC_{50} = 531.6 ± 29.4 (11β-HSD1 in rat testis)	CURCUMIN	[33]

	IC_{50} > 100000 (11β-HSD1 in human liver)<br>IC_{50} > 100000 (11β-HSD2 in rat testis)<br>IC_{50} > 100000 (11β-HSD2 in human liver)	IC_{50} = 11,360.8 ± 1807.9 (11β-HSD1 in rat testis)<br>IC_{50} = 5236.4 ± 98.6 (11β-HSD1 in human liver)<br>IC_{50} 12,231 ± 874.2 (11β-HSD2 in rat testis)<br>IC_{50} 14,672 ± 1136.1 (11β-HSD2 in human liver)	
**609**	IC_{50} = 407.4 ± 68.3 (11β-HSD1 in rat testis)<br>IC_{50} > 100000 (11β-HSD1 in human liver)<br>IC_{50} > 100000 (11β-HSD2 in rat testis)<br>IC_{50} > 100000 (11β-HSD2 in human liver)	CURCUMIN<br>IC_{50} = 11,360.8 ± 1807.9 (11β-HSD1 in rat testis)<br>IC_{50} = 5236.4 ± 98.6 (11β-HSD1 in human liver)<br>IC_{50} 12,231 ± 874.2 (11β-HSD2 in rat testis)<br>IC_{50} 14,672 ± 1136.1 (11β-HSD2 in human liver)	[33]
**610**	IC_{50} = 2240.7 ± 186.5 (11β-HSD1 in rat testis)<br>IC_{50} = 650.5 ± 101.6 (11β-HSD1 in human liver)<br>IC_{50} > 100000 (11β-HSD2 in rat testis)<br>IC_{50} > 100000 (11β-HSD2 in human liver)	CURCUMIN<br>IC_{50} = 11,360.8 ± 1807.9 (11β-HSD1 in rat testis)<br>IC_{50} = 5236.4 ± 98.6 (11β-HSD1 in human liver)<br>IC_{50} 12,231 ± 874.2 (11β-HSD2 in rat testis)<br>IC_{50} 14,672 ± 1136.1 (11β-HSD2 in human liver)	[33]
**611**	IC_{50} = 3443.0 ± 169.2 (11β-HSD1 in rat testis)<br>IC_{50} = 1100.8 ± 123.6 (11β-HSD1 in human liver)<br>IC_{50} > 100000 (11β-HSD2 in rat testis)<br>IC_{50} > 100000 (11β-HSD2 in human liver)	CURCUMIN<br>IC_{50} = 11,360.8 ± 1807.9 (11β-HSD1 in rat testis)<br>IC_{50} = 5236.4 ± 98.6 (11β-HSD1 in human liver)<br>IC_{50} 12,231 ± 874.2 (11β-HSD2 in rat testis)<br>IC_{50} 14,672 ± 1136.1 (11β-HSD2 in human liver)	[33]
**612**	IC_{50} = 673.4 ± 86.3 (11β-HSD1 in rat testis)<br>IC_{50} = 1064.3 ± 220.2 (11β-HSD1 in human liver)<br>IC_{50} > 100000 (11β-HSD2 in rat testis)<br>IC_{50} > 100000 (11β-HSD2 in human liver)	CURCUMIN<br>IC_{50} = 11,360.8 ± 1807.9 (11β-HSD1 in rat testis)<br>IC_{50} = 5236.4 ± 98.6 (11β-HSD1 in human liver)<br>IC_{50} 12,231 ± 874.2 (11β-HSD2 in rat testis)<br>IC_{50} 14,672 ± 1136.1 (11β-HSD2 in human liver)	[33]
**613**	IC_{50} = 4767.9 ± 328.9 (11β-HSD1 in rat testis)<br>IC_{50} > 100000 (11β-HSD1 in human liver)<br>IC_{50} > 100000 (11β-HSD2 in rat testis)<br>IC_{50} > 100000 (11β-HSD2 in human liver)	CURCUMIN<br>IC_{50} = 11,360.8 ± 1807.9 (11β-HSD1 in rat testis)<br>IC_{50} = 5236.4 ± 98.6 (11β-HSD1 in human liver)<br>IC_{50} 12,231 ± 874.2 (11β-HSD2 in rat testis)<br>IC_{50} 14,672 ± 1136.1 (11β-HSD2 in human liver)	[33]
**614**	Reduced blood glucose (at 10 mg/kg)<br>Inhibited effects on hyperglycemia-induced inflammation and fibrosis	------------	[34]

	Inhibited the P38 and AKT signal pathway		
**615**	43.0% (Inhibition at 50 μM)	QUERCETIN<br>83.6% (Inhibition at 50 μM)	[35]
**616**	39.8% (Inhibition at 50 μM)	QUERCETIN<br>83.6% (Inhibition at 50 μM)	[35]
**617**	34.0% (Inhibition at 50 μM)	QUERCETIN<br>83.6% (Inhibition at 50 μM)	[35]
**618**	37.8% (Inhibition at 50 μM)	QUERCETIN<br>83.6% (Inhibition at 50 μM)	[35]
**619**	43.0% (Inhibition at 50 μM)	QUERCETIN<br>83.6% (Inhibition at 50 μM)	[35]
**620**	27.6% (Inhibition at 50 μM)	QUERCETIN<br>83.6% (Inhibition at 50 μM)	[35]
**621**	44.0% (Inhibition at 50 μM)	QUERCETIN<br>83.6% (Inhibition at 50 μM)	[35]
**622**	48.4% (Inhibition at 50 μM)	QUERCETIN<br>83.6% (Inhibition at 50 μM)	[35]
**623**	46.8% (Inhibition at 50 μM)	QUERCETIN<br>83.6% (Inhibition at 50 μM)	[35]
**624**	27.2% (Inhibition at 50 μM)	QUERCETIN<br>83.6% (Inhibition at 50 μM)	[35]
**625**	29.1% (Inhibition at 50 μM)	QUERCETIN<br>83.6% (Inhibition at 50 μM)	[35]
**626**	47.9% (Inhibition at 50 μM)	QUERCETIN<br>83.6% (Inhibition at 50 μM)	[35]
**627**	34.1% (Inhibition at 50 μM)	QUERCETIN<br>83.6% (Inhibition at 50 μM)	[35]
**628**	31.4% (Inhibition at 50 μM)	QUERCETIN<br>83.6% (Inhibition at 50 μM)	[35]
**629**	31.8% (Inhibition at 50 μM)	QUERCETIN<br>83.6% (Inhibition at 50 μM)	[35]
**630**	41.0% (Inhibition at 50 μM)	QUERCETIN<br>83.6% (Inhibition at 50 μM)	[35]
**631**	43.8% (Inhibition at 50 μM)	QUERCETIN<br>83.6% (Inhibition at 50 μM)	[35]
**632**	16.3% (Inhibition at 50 μM)	QUERCETIN<br>83.6% (Inhibition at 50 μM)	[35]
**633**	65.1% (Inhibition at 50 μM)<br>IC_{50} = 28.4 μM	QUERCETIN<br>83.6% (Inhibition at 50 μM)<br>IC_{50} = 7.8 μM	[35]
**634**	53.0% (Inhibition at 50 μM)<br>IC_{50} = 42.3 μM	QUERCETIN<br>83.6% (Inhibition at 50 μM)<br>IC_{50} = 7.8 μM	[35]
**635**	90.7% (Inhibition at 50 μM)<br>IC_{50} = 20.3 μM	QUERCETIN<br>83.6% (Inhibition at 50 μM)<br>IC_{50} = 7.8 μM	[35]
**636**	61.2% (Inhibition at 50 μM)<br>IC_{50} = 40.7 μM	QUERCETIN<br>83.6% (Inhibition at 50 μM)<br>IC_{50} = 7.8 μM	[35]
**637**	98.4% (Inhibition at 50 μM)<br>IC_{50} = 14.1 μM	QUERCETIN<br>83.6% (Inhibition at 50 μM)	[35]

		IC50 = 7.8 μM	
**638**	96.9% (Inhibition at 50 μM)<br>IC50 = 19.6 μM	QUERCETIN<br>83.6% (Inhibition at 50 μM)<br>IC50 = 7.8 μM	[35]
**639**	86.6% (Inhibition at 50 μM)<br>IC50 = 20.0 μM	QUERCETIN<br>83.6% (Inhibition at 50 μM)<br>IC50 = 7.8 μM	[35]
**Antihyperuricemic activity**			
**Compound**		**Reference Drug**	**Reference**
**631**	Inhibition = 32.15% (serum uric acid level)	ALLOPURINOL<br>Inhibition = 118.32% (serum uric acid level)<br>BENZBROMARONE<br>Inhibition = 99.74% (serum uric acid level)	[36]
**641**	Inhibition = 36.52% (serum uric acid level)	ALLOPURINOL<br>Inhibition = 118.32% (serum uric acid level)<br>BENZBROMARONE<br>Inhibition = 99.74% (serum uric acid level)	[36]
**642**	Inhibition = 47.33% (serum uric acid level)	ALLOPURINOL<br>Inhibition = 118.32% (serum uric acid level)<br>BENZBROMARONE<br>Inhibition = 99.74% (serum uric acid level)	[36]
**643**	Inhibition = 92.45% (serum uric acid level)	ALLOPURINOL<br>Inhibition = 118.32% (serum uric acid level)<br>BENZBROMARONE<br>Inhibition = 99.74% (serum uric acid level)	[36]
**644**	Inhibition = 15.82% (serum uric acid level)	ALLOPURINOL<br>Inhibition = 118.32% (serum uric acid level)<br>BENZBROMARONE<br>Inhibition = 99.74% (serum uric acid level)	[36]
**645**	Inhibition = 7.74% (serum uric acid level)	ALLOPURINOL<br>Inhibition = 118.32% (serum uric acid level)<br>BENZBROMARONE<br>Inhibition = 99.74% (serum uric acid level)	[36]
**646**	Inhibition = 11.88% (serum uric acid level)	ALLOPURINOL<br>Inhibition = 118.32% (serum uric acid level)<br>BENZBROMARONE<br>Inhibition = 99.74% (serum uric acid level)	[36]
**647**	Inhibition = 20.06% (serum uric acid level)	ALLOPURINOL<br>Inhibition = 118.32% (serum uric acid level)<br>BENZBROMARONE<br>Inhibition = 99.74% (serum uric acid level)	[36]
**648**	Inhibition = 25.21% (serum uric acid level)	ALLOPURINOL<br>Inhibition = 118.32% (serum uric acid level)	[36]

		BENZBROMARONE<br>Inhibition = 99.74% (serum uric acid level)	
**649**	Inhibition = 50.78% (serum uric acid level)	ALLOPURINOL<br>Inhibition = 118.32% (serum uric acid level)<br>BENZBROMARONE<br>Inhibition = 99.74% (serum uric acid level)	[36]
**650**	Inhibition = 40.17% (serum uric acid level)	ALLOPURINOL<br>Inhibition = 118.32% (serum uric acid level)<br>BENZBROMARONE<br>Inhibition = 99.74% (serum uric acid level)	[36]
**651**	Inhibition = 16.82% (serum uric acid level)	ALLOPURINOL<br>Inhibition = 118.32% (serum uric acid level)<br>BENZBROMARONE<br>Inhibition = 99.74% (serum uric acid level)	[36]
**652**	Inhibition = 25.20% (serum uric acid level)	ALLOPURINOL<br>Inhibition = 118.32% (serum uric acid level)<br>BENZBROMARONE<br>Inhibition = 99.74% (serum uric acid level)	[36]
**653**	Inhibition = 44.52% (serum uric acid level)	ALLOPURINOL<br>Inhibition = 118.32% (serum uric acid level)<br>BENZBROMARONE<br>Inhibition = 99.74% (serum uric acid level)	[36]
**654**	Inhibition = 12.95% (serum uric acid level)	ALLOPURINOL<br>Inhibition = 118.32% (serum uric acid level)<br>BENZBROMARONE<br>Inhibition = 99.74% (serum uric acid level)	[36]
**655**	Inhibition = 11.78% (serum uric acid level)	ALLOPURINOL<br>Inhibition = 118.32% (serum uric acid level)<br>BENZBROMARONE<br>Inhibition = 99.74% (serum uric acid level)	[36]
**656**	Inhibition = 9.36% (serum uric acid level)	ALLOPURINOL<br>Inhibition = 118.32% (serum uric acid level)<br>BENZBROMARONE<br>Inhibition = 99.74% (serum uric acid level)	[36]
**657**	Inhibition = 15.10% (serum uric acid level)	ALLOPURINOL<br>Inhibition = 118.32% (serum uric acid level)<br>BENZBROMARONE<br>Inhibition = 99.74% (serum uric acid level)	[36]
**658**	Inhibition = 18.82% (serum uric acid level)	ALLOPURINOL<br>Inhibition = 118.32% (serum uric acid level)<br>BENZBROMARONE<br>Inhibition = 99.74% (serum uric acid level)	[36]

**659**	Inhibition = 54.39% (serum uric acid level)	ALLOPURINOL<br>Inhibition = 118.32% (serum uric acid level)<br>BENZBROMARONE<br>Inhibition = 99.74% (serum uric acid level)	[36]
**660**	Inhibition = 14.09% (serum uric acid level)	ALLOPURINOL<br>Inhibition = 118.32% (serum uric acid level)<br>BENZBROMARONE<br>Inhibition = 99.74% (serum uric acid level)	[36]
**661**	Inhibition = 30.62% (serum uric acid level)	ALLOPURINOL<br>Inhibition = 118.32% (serum uric acid level)<br>BENZBROMARONE<br>Inhibition = 99.74% (serum uric acid level)	[36]
**Neuroprotective activity**			
**Compound**		**Reference Drug**	**Reference**
**212**	IC_{50} = 9.7 ± 1.1 μM (AChE)	TACRINE<br>IC_{50} = 0.095 ± 0.011 μM (AChE)<br>IC_{50} = 0.010 ± 0.004 μM (BChE)	[37]
**192**	IC_{50} = 7.4 ± 0.5 μM (AChE)	TACRINE<br>IC_{50} = 0.095 ± 0.011 μM (AChE)<br>IC_{50} = 0.010 ± 0.004 μM (BChE)	[37]
**194**	IC_{50} = 8.2 ± 0.9 μM (AChE)	TACRINE<br>IC_{50} = 0.095 ± 0.011 μM (AChE)<br>IC_{50} = 0.010 ± 0.004 μM (BChE)	[37]
**196**	IC_{50} = 7.6 ± 0.4 μM (AChE)	TACRINE<br>IC_{50} = 0.095 ± 0.011 μM (AChE)<br>IC_{50} = 0.010 ± 0.004 μM (BChE)	[37]
**199**	IC_{50} = 6.5 ± 0.6 μM (BChE)	TACRINE<br>IC_{50} = 0.095 ± 0.011 μM (AChE)<br>IC_{50} = 0.010 ± 0.004 μM (BChE)	[37]
**200**	IC_{50} = 7.6 ± 0.1 μM (AChE)	TACRINE<br>IC_{50} = 0.095 ± 0.011 μM (AChE)<br>IC_{50} = 0.010 ± 0.004 μM (BChE)	[37]
**201**	IC_{50} = 8.3 ± 1.5 μM (AChE)	TACRINE<br>IC_{50} = 0.095 ± 0.011 μM (AChE)<br>IC_{50} = 0.010 ± 0.004 μM (BChE)	[37]
**207**	IC_{50} = 10.6 ± 0.3 μM (BChE)	TACRINE<br>IC_{50} = 0.095 ± 0.011 μM (AChE)<br>IC_{50} = 0.010 ± 0.004 μM (BChE)	[37]
**208**	IC_{50} = 4.7 ± 0.2 μM (BChE)	TACRINE<br>IC_{50} = 0.095 ± 0.011 μM (AChE)<br>IC_{50} = 0.010 ± 0.004 μM (BChE)	[37]
**210**	IC_{50} = 5.1 ± 0.3 μM (AChE)	TACRINE<br>IC_{50} = 0.095 ± 0.011 μM (AChE)<br>IC_{50} = 0.010 ± 0.004 μM (BChE)	[37]
**211**	IC_{50} = 5.9 ± 0.5 μM (AChE)	TACRINE<br>IC_{50} = 0.095 ± 0.011 μM (AChE)<br>IC_{50} = 0.010 ± 0.004 μM (BChE)	[37]
**662**	IC_{50} =6.6 ± 0.5 μM (AChE)	TACRINE<br>IC_{50} = 0.095 ± 0.011 μM (AChE)	[37]

		IC_{50} = 0.010 ± 0.004 μM (BChE)	
**663**	IC_{50} = 6.6 ± 0.3 μM (AChE)	TACRINE<br>IC_{50} = 0.095 ± 0.011 μM (AChE)<br>IC_{50} = 0.010 ± 0.004 μM (BChE)	[37]
**664**	IC_{50} = 8.7 ± 0.8 μM (AChE)	TACRINE<br>IC_{50} = 0.095 ± 0.011 μM (AChE)<br>IC_{50} = 0.010 ± 0.004 μM (BChE)	[37]
**665**	IC_{50} = 8.4 ± 0.6 μM (AChE)	TACRINE<br>IC_{50} = 0.095 ± 0.011 μM (AChE)<br>IC_{50} = 0.010 ± 0.004 μM (BChE)	[37]
**666**	IC_{50} = 8.7 ± 0.7 μM (AChE)	TACRINE<br>IC_{50} = 0.095 ± 0.011 μM (AChE)<br>IC_{50} = 0.010 ± 0.004 μM (BChE)	[37]
**667**	IC_{50} = 9.0 ± 0.4 μM (AChE)	TACRINE<br>IC_{50} = 0.095 ± 0.011 μM (AChE)<br>IC_{50} = 0.010 ± 0.004 μM (BChE)	[37]
**668**	IC_{50} = 5.9 ± 0.6 μM (AChE)	TACRINE<br>IC_{50} = 0.095 ± 0.011 μM (AChE)<br>IC_{50} = 0.010 ± 0.004 μM (BChE)	[37]
**669**	IC_{50} = 5.4 ± 0.2 μM (AChE)	TACRINE<br>IC_{50} = 0.095 ± 0.011 μM (AChE)<br>IC_{50} = 0.010 ± 0.004 μM (BChE)	[37]
**670**	IC_{50} = 9.8 ± 0.2 μM (AChE)	TACRINE<br>IC_{50} = 0.095 ± 0.011 μM (AChE)<br>IC_{50} = 0.010 ± 0.004 μM (BChE)	[37]
**671**	IC_{50} = 8.6 ± 0.6 μM (AChE)	TACRINE<br>IC_{50} = 0.095 ± 0.011 μM (AChE)<br>IC_{50} = 0.010 ± 0.004 μM (BChE)	[37]
**672**	IC_{50} = 8.5 ± 0.2 μM (AChE)	TACRINE<br>IC_{50} = 0.095 ± 0.011 μM (AChE)<br>IC_{50} = 0.010 ± 0.004 μM (BChE)	[37]
**673**	IC_{50} = 7.8 ± 0.7 (AChE)	TACRINE<br>IC_{50} = 0.095 ± 0.011 μM (AChE)<br>IC_{50} = 0.010 ± 0.004 μM (BChE)	[37]
**674**	IC_{50} = 8.4 ± 0.7 μM (AChE)	TACRINE<br>IC_{50} = 0.095 ± 0.011 μM (AChE)<br>IC_{50} = 0.010 ± 0.004 μM (BChE)	[37]
**675**	IC_{50} = 3.1 ± 0.4 μM (AChE)<br>IC_{50} = 1.4 ± 0.4 μM (BChE)	TACRINE<br>IC_{50} = 0.095 ± 0.011 μM (AChE)<br>IC_{50} = 0.010 ± 0.004 μM (BChE)	[37]
**676**	IC_{50} = 1.6 ± 0.2 μM (AChE)<br>IC_{50} = 2.7 ± 0.6 μM (BChE)	TACRINE<br>IC_{50} = 0.095 ± 0.011 μM (AChE)<br>IC_{50} = 0.010 ± 0.004 μM (BChE)	[37]
**677**	IC_{50} = 3.5 ± 0.3 μM (AChE)<br>IC_{50} = 0.6 ± 0.1 μM (BChE)	TACRINE<br>IC_{50} = 0.095 ± 0.011 μM (AChE)<br>IC_{50} = 0.010 ± 0.004 μM (BChE)	[37]
**678**	IC_{50} = 2.0 ± 0.3 μM (AChE)<br>IC_{50} = 2.3 ± 0.2 μM (BChE)	TACRINE<br>IC_{50} = 0.095 ± 0.011 μM (AChE)<br>IC_{50} = 0.010 ± 0.004 μM (BChE)	[37]
**Diverse Biochemical targets**			
**Compound**		**Drug reference**	**Reference**

**679**	IC_{50}=189.42 μM (tyrosinase)	4-BUTYLRESORCINOL<br>IC_{50} = 11.27 μM (tyrosinase)<br>KOJIC ACID<br>IC_{50} = 28.59 μM (tyrosinase)	[38]
**680**	IC_{50}=56.64 μM (tyrosinase)	4-BUTYLRESORCINOL<br>IC_{50} = 11.27 μM (tyrosinase)<br>KOJIC ACID<br>IC_{50} = 28.59 μM (tyrosinase)	[38]
**681**	IC_{50} = 7.78 μM (tyrosinase)	4-BUTYLRESORCINOL<br>IC_{50} = 11.27 μM (tyrosinase)<br>KOJIC ACID<br>IC_{50} = 28.59 μM (tyrosinase)	[38]
**682**	IC_{50} = 1.74 μM (tyrosinase)	4-BUTYLRESORCINOL<br>IC_{50} = 11.27 μM (tyrosinase)<br>KOJIC ACID<br>IC_{50} = 28.59 μM (tyrosinase)	[38]
**683**	IC_{50} = 9.66 μM (tyrosinase)	4-BUTYLRESORCINOL<br>IC_{50} = 11.27 μM (tyrosinase)<br>KOJIC ACID<br>IC_{50} = 28.59 μM (tyrosinase)	[38]
**684**	IC_{50} = 168.36 μM (tyrosinase)	4-BUTYLRESORCINOL<br>IC_{50}=11.27 μM (tyrosinase)<br>KOJIC ACID<br>IC_{50}=28.59 μM (tyrosinase)	[38]
**685**	IC_{50} = 173.48 μM (tyrosinase)	4-BUTYLRESORCINOL<br>IC_{50} = 11.27 μM (tyrosinase)<br>KOJIC ACID<br>IC_{50} = 28.59 μM (tyrosinase)	[38]
**686**	IC_{50} > 200 μM (tyrosinase)	4-BUTYLRESORCINOL<br>IC_{50} = 11.27 μM (tyrosinase)<br>KOJIC ACID<br>IC_{50} = 28.59 μM (tyrosinase)	[38]
**687**	IC_{50}=16.74 μM (tyrosinase)	4-BUTYLRESORCINOL<br>IC_{50} = 11.27 μM (tyrosinase)<br>KOJIC ACID<br>IC_{50} = 28.59 μM (tyrosinase)	[38]
**688**	IC_{50} = 2.78 μM (tyrosinase)	4-BUTYLRESORCINOL<br>IC_{50} = 11.27 μM (tyrosinase)<br>KOJIC ACID<br>IC_{50} = 28.59 μM (tyrosinase)	[38]
**689**	IC_{50} > 200 μM (tyrosinase)	4-BUTYLRESORCINOL<br>IC_{50} = 11.27 μM (tyrosinase)<br>KOJIC ACID<br>IC_{50} = 28.59 μM (tyrosinase)	[38]
**690**	IC_{50} = 182.86 μM (tyrosinase)	4-BUTYLRESORCINOL<br>IC_{50} = 11.27 μM (tyrosinase)<br>KOJIC ACID<br>IC_{50} = 28.59 μM (tyrosinase)	[38]
**691**	IC_{50} = 46.24 μM (tyrosinase)	4-BUTYLRESORCINOL<br>IC_{50} = 11.27 μM (tyrosinase)<br>KOJIC ACID<br>IC_{50} = 28.59 μM (tyrosinase)	[38]

**492**	IC_{50} = 4.64 µM (tyrosinase)	4-BUTYLRESORCINOL<br>IC_{50}=11.27 µM (tyrosinase)<br>KOJIC ACID<br>IC_{50}=28.59 µM (tyrosinase)	[38]
**693**	IC_{50} = 7.20 µM (tyrosinase)	4-BUTYLRESORCINOL<br>IC_{50} = 11.27 µM (tyrosinase)<br>KOJIC ACID<br>IC_{50} = 28.59 µM (tyrosinase)	[38]
**694**	IC_{50} > 200 µM (tyrosinase)	4-BUTYLRESORCINOL<br>IC_{50} = 11.27 µM (tyrosinase)<br>KOJIC ACID<br>IC_{50} = 28.59 µM (tyrosinase)	[38]
**695**	IC_{50} = 16.34 µM (tyrosinase)	4-BUTYLRESORCINOL<br>IC_{50} = 11.27 µM (tyrosinase)<br>KOJIC ACID<br>IC_{50} = 28.59 µM (tyrosinase)	[38]
**696**	IC_{50} > 200 µM (tyrosinase)	4-BUTYLRESORCINOL<br>IC_{50} = 11.27 µM (tyrosinase)<br>KOJIC ACID<br>IC_{50} = 28.59 µM (tyrosinase)	[38]
**697**	IC_{50} = 86.92 µM (tyrosinase)	4-BUTYLRESORCINOL<br>IC_{50} = 11.27 µM (tyrosinase)<br>KOJIC ACID<br>IC_{50} = 28.59 µM (tyrosinase)	[38]
**698**	IC_{50} > 200 µM (tyrosinase)	4-BUTYLRESORCINOL<br>IC_{50} = 11.27 µM (tyrosinase)<br>KOJIC ACID<br>IC_{50} = 28.59 µM (tyrosinase)	[38]
**28**	140.39 ± 2.38% (residual activity of acid phosphatase)<br>57.83 ± 6.50% (residual activity of alkaline phosphatase)	CONTROL<br>100% (residual activity of acid phosphatase)<br>100% (residual activity of alkaline phosphatase)<br>LEVAMISOLE<br>88.00 ± 1.25% (residual activity of alkaline phosphatase)	[39]
**165**	120.52 ± 1.69% (residual activity of acid phosphatase)<br>41.17 ± 1.15% (residual activity of alkaline phosphatase)	CONTROL<br>100% (residual activity of acid phosphatase)<br>100% (residual activity of alkaline phosphatase)<br>LEVAMISOLE<br>88.00 ± 1.25% (residual activity of alkaline phosphatase)	[39]
**166**	128.73 ± 1.39% (residual activity of acid phosphatase)<br>46.16 ± 1.03% (residual activity of alkaline phosphatase)	CONTROL<br>100% (residual activity of acid phosphatase)<br>100% (residual activity of alkaline phosphatase)<br>LEVAMISOLE<br>88.00 ± 1.25% (residual activity of alkaline phosphatase)	[39]

**167**	158.13 ± 2.20% (residual activity of acid phosphatase)<br>31.67 ± 0.53% (residual activity of alkaline phosphatase)	CONTROL<br>100% (residual activity of acid phosphatase)<br>100% (residual activity of alkaline phosphatase)<br>LEVAMISOLE<br>88.00 ± 1.25% (residual activity of alkaline phosphatase)	[39]
**351**	112.97 ± 1.44% (residual activity of acid phosphatase)<br>40.17 ± 1.20% (residual activity of alkaline phosphatase)	CONTROL<br>100% (residual activity of acid phosphatase)<br>100% (residual activity of alkaline phosphatase)<br>LEVAMISOLE<br>88.00 ± 1.25% (residual activity of alkaline phosphatase)	[39]
**699**	126.60 ± 1.71% (residual activity of acid phosphatase)<br>43.50 ± 0.38% (residual activity of alkaline phosphatase)	CONTROL<br>100% (residual activity of acid phosphatase)<br>100% (residual activity of alkaline phosphatase)<br>LEVAMISOLE<br>88.00 ± 1.25% (residual activity of alkaline phosphatase)	[39]
**700**	148.60 ± 1.97 % (residual activity of acid phosphatase)<br>46.00 ± 1.77% (residual activity of alkaline phosphatase)	CONTROL<br>100% (residual activity of acid phosphatase)<br>100% (residual activity of alkaline phosphatase)<br>LEVAMISOLE<br>88.00 ± 1.25% (residual activity of alkaline phosphatase)	[39]
**701**	132.84 ± 1.94% (residual activity of acid phosphatase)<br>26.00 ± 0.20% (residual activity of alkaline phosphatase)	CONTROL<br>100% (residual activity of acid phosphatase)<br>100% (residual activity of alkaline phosphatase)<br>LEVAMISOLE<br>88.00 ± 1.25% (residual activity of alkaline phosphatase)	[39]
**98**	3.79% (CA-II Inhibition)	------	[40]
**702**	81.44% (CA-II Inhibition)	------	[40]
**703**	8.89% (CA-II Inhibition)	------	[40]
**704**	49.37% (CA-II Inhibition)	------	[40]
**705**	11.43% (CA-II Inhibition)	------	[40]
**706**	14.90% (CA-II Inhibition)	------	[40]
**707**	0.00% (CA-II Inhibition)	------	[40]
**708**	7.56% (CA-II Inhibition)	------	[40]
**709**	0.68% (CA-II Inhibition)	------	[40]
**710**	0.00% (CA-II Inhibition)	------	[40]
**711**	0.00% (CA-II Inhibition)	------	[40]
**712**	0.00% (CA-II Inhibition)	------	[40]

**713**	0.00% (CA-II Inhibition)	------	[40]
**714**	8.02% (CA-II Inhibition)	------	[40]
**715**	0.00% (CA-II Inhibition)	------	[40]
**716**	17.71% (CA-II Inhibition)	------	[40]
**717**	18.42% (CA-II Inhibition)	------	[40]
**718**	40.60% (CA-II Inhibition)	------	[40]
**719**	0.00% (CA-II Inhibition)	------	[40]
**720**	0.00% (CA-II Inhibition)	------	[40]
**721**	15.62% (CA-II Inhibition)	------	[40]
**722**	12.41% (CA-II Inhibition)	------	[40]
**723**	0.00% (CA-II Inhibition)	------	[40]
**723**	14.85% (CA-II Inhibition)	------	[40]
**725**	12.30% (CA-II Inhibition)	------	[40]
**726**	27.09% (CA-II Inhibition)	------	[40]
**727**	22.20% (CA-II Inhibition)	------	[40]
**728**	0.00% (CA-II Inhibition)	------	[40]
**729**	19.09% (CA-II Inhibition)	------	[40]
**730**	40.65% (CA-II Inhibition)	------	[40]
**731**	48.66% (CA-II Inhibition)	------	[40]
**732**	19.09% (CA-II Inhibition)	------	[40]
**733**	41.32% (CA-II Inhibition)	------	[40]
**734**	58.50% (CA-II Inhibition)	------	[40]
**735**	0.00% (CA-II Inhibition)	------	[40]
**736**	53.45% (CA-II Inhibition)	------	[40]
**737**	43.25% (CA-II Inhibition)	------	[40]
**738**	0.00% (CA-II Inhibition)	------	[40]
**3**	IC_{50} = 1.6 μM (TRPA1)<br>IC_{50} > 50 μM (TRPM8)<br>IC_{50} = 4.1 μM (TRPV1)	CURCUMIN<br>IC_{50} = 3.3 μM (TRPA1)<br>IC_{50} = 3.0 μM (TRPM8)<br>IC_{50} > 50 μM (TRPV1)	[41]
**9**	IC_{50} = 3.6 μM (TRPA1)<br>IC_{50} = 25.0 μM (TRPM8)<br>IC_{50} > 50 μM (TRPV1)	CURCUMIN<br>IC_{50} = 3.3 μM (TRPA1)<br>IC_{50} = 3.0 μM (TRPM8)<br>IC_{50} > 50 μM (TRPV1)	[41]
**13**	IC_{50} = 10.5 μM (TRPA1)<br>IC_{50} > 50 μM (TRPM8)<br>IC_{50} > 50 μM (TRPV1)	CURCUMIN<br>IC_{50} = 3.3 μM (TRPA1)<br>IC_{50} = 3.0 μM (TRPM8)<br>IC_{50} > 50 μM (TRPV1)	[41]
**23**	IC_{50} = 9.2 μM (TRPA1)<br>IC_{50} > 50 μM (TRPM8)<br>IC_{50} > 50 μM (TRPV1)	CURCUMIN<br>IC_{50} = 3.3 μM (TRPA1)<br>IC_{50} = 3.0 μM (TRPM8)<br>IC_{50} > 50 μM (TRPV1)	[41]
**28**	IC_{50} = 0.70 μM (TRPA1)<br>IC_{50} > 50 μM (TRPM8)<br>IC_{50} > 50 μM (TRPV1)	CURCUMIN<br>IC_{50} = 3.3 μM (TRPA1)<br>IC_{50} = 3.0 μM (TRPM8)<br>IC_{50} > 50 μM (TRPV1)	[41]
**163**	IC_{50} = 11.3 μM (TRPA1)<br>IC_{50} > 50 μM (TRPM8)<br>IC_{50} > 50 μM (TRPV1)	CURCUMIN<br>IC_{50} = 3.3 μM (TRPA1)<br>IC_{50} = 3.0 μM (TRPM8)<br>IC_{50} > 50 μM (TRPV1)	[41]
**165**	IC_{50} = 1.1 μM (TRPA1)	CURCUMIN	[41]

	$IC_{50} > 50$ μM (TRPM8)<br>$IC_{50} > 50$ μM (TRPV1)	$IC_{50} = 3.3$ μM (TRPA1)<br>$IC_{50} = 3.0$ μM (TRPM8)<br>$IC_{50} > 50$ μM (TRPV1)	
**327**	$IC_{50} = 0.60$ μM (TRPA1)<br>$IC_{50} > 50$ μM (TRPM8)<br>$IC_{50} > 50$ μM (TRPV1)	CURCUMIN<br>$IC_{50} = 3.3$ μM (TRPA1)<br>$IC_{50} = 3.0$ μM (TRPM8)<br>$IC_{50} > 50$ μM (TRPV1)	[41]
**334**	$IC_{50} = 1.8$ μM (TRPA1)<br>$IC_{50} = 3.3$ μM (TRPM8)<br>$IC_{50} = 5.2$ μM (TRPV1)	CURCUMIN<br>$IC_{50} = 3.3$ μM (TRPA1)<br>$IC_{50} = 3.0$ μM (TRPM8)<br>$IC_{50} > 50$ μM (TRPV1)	[41]
**346**	$IC_{50} = 0.27$ μM (TRPA1)<br>$IC_{50} > 50$ μM (TRPM8)<br>$IC_{50} > 50$ μM (TRPV1)	CURCUMIN<br>$IC_{50} = 3.3$ μM (TRPA1)<br>$IC_{50} = 3.0$ μM (TRPM8)<br>$IC_{50} > 50$ μM (TRPV1)	[41]
**739**	$IC_{50} = 17.7$ μM (TRPA1)<br>$IC_{50} > 50$ μM (TRPM8)<br>$IC_{50} > 50$ μM (TRPV1)	CURCUMIN<br>$IC_{50} = 3.3$ μM (TRPA1)<br>$IC_{50} = 3.0$ μM (TRPM8)<br>$IC_{50} > 50$ μM (TRPV1)	[41]
**740**	$IC_{50} = 24.4$ μM (TRPA1)<br>$IC_{50} > 50$ μM (TRPM8)<br>$IC_{50} > 50$ μM (TRPV1)	CURCUMIN<br>$IC_{50} = 3.3$ μM (TRPA1)<br>$IC_{50} = 3.0$ μM (TRPM8)<br>$IC_{50} > 50$ μM (TRPV1)	[41]
**741**	$IC_{50} = 14.5$ μM (TRPA1)<br>$IC_{50} > 50$ μM (TRPM8)<br>$IC_{50} > 50$ μM (TRPV1)	CURCUMIN<br>$IC_{50} = 3.3$ μM (TRPA1)<br>$IC_{50} = 3.0$ μM (TRPM8)<br>$IC_{50} > 50$ μM (TRPV1)	[41]
**742**	$IC_{50} > 50$ μM (TRPA1)<br>$IC_{50} > 50$ μM (TRPM8)<br>$IC_{50} > 50$ μM (TRPV1)	CURCUMIN<br>$IC_{50} = 3.3$ μM (TRPA1)<br>$IC_{50} = 3.0$ μM (TRPM8)<br>$IC_{50} > 50$ μM (TRPV1)	[41]
**743**	$IC_{50} = 11.5$ μM (TRPA1)<br>$IC_{50} > 50$ μM (TRPM8)<br>$IC_{50} > 50$ μM (TRPV1)	CURCUMIN<br>$IC_{50} = 3.3$ μM (TRPA1)<br>$IC_{50} = 3.0$ μM (TRPM8)<br>$IC_{50} > 50$ μM (TRPV1)	[41]
**744**	$IC_{50} = 38.3$ μM (TRPA1)<br>$IC_{50} > 50$ μM (TRPM8)<br>$IC_{50} > 50$ μM (TRPV1)	CURCUMIN<br>$IC_{50} = 3.3$ μM (TRPA1)<br>$IC_{50} = 3.0$ μM (TRPM8)<br>$IC_{50} > 50$ μM (TRPV1)	[41]
**745**	$IC_{50} = 4.2$ μM (TRPA1)<br>$IC_{50} > 50$ μM (TRPM8)<br>$IC_{50} > 50$ μM (TRPV1)	CURCUMIN<br>$IC_{50} = 3.3$ μM (TRPA1)<br>$IC_{50} = 3.0$ μM (TRPM8)<br>$IC_{50} > 50$ μM (TRPV1)	[41]

ABTS = 2,2′-azinobis(3-ethylbenzothiazoline-6-sulfonicacid) radical cation; AChE = Acetylcholinesterase; AP-1 = Activator protein 1; BChE = Butyrylcholinesterase; CA-II = Carbonic anhydrase II; COX-1/2= Cyclooxygenase 1/2; DPPH = Diphenylpicrylhydrazyl assay ; FRAP = Antioxidant power assay ; HPLC = ; IC_{50} = Half-maximal inhibitory concentration ; IL-6 = Interleukin-6; IZ = Inhibition zone; LOX = Lipoxygenase; LPS = Lipopolysaccharide; L-NAME = Nω- nitro-L-arginine methyl esther hydrochloride; mPGES-1 = microsomal prostaglandin E synthase-1; NET = O^{2-}• radical scavenging assay; NO = nitric oxide; PGE2 = Prostaglandin 2 ; TNF-α = Tumor necrosis factor-alpha; TRAP = ROO• radical scavenging assay; TRPA1 = Transient receptor potential channels of ankyrin type-1; TRPM8 = Transient receptor potential cation channel subfamily M (melastatin) member 8; TRPV1 = Transient receptor potential channels of vanilloid type-1; 11β-HSD = 11β-hydroxysteroid dehydrogenase.
